# The impact of nitrogen oxides on electrochemical carbon dioxide reduction

**DOI:** 10.1038/s41467-020-19731-8

**Published:** 2020-11-17

**Authors:** Byung Hee Ko, Bjorn Hasa, Haeun Shin, Emily Jeng, Sean Overa, Wilson Chen, Feng Jiao

**Affiliations:** Center for Catalytic Science and Technology, Department of Chemical and Biomolecular Engineering, University of Delaware, Newark, DE 19716 USA

**Keywords:** Electrocatalysis, Chemical engineering

## Abstract

The electroreduction of carbon dioxide offers a promising avenue to produce valuable fuels and chemicals using greenhouse gas carbon dioxide as the carbon feedstock. Because industrial carbon dioxide point sources often contain numerous contaminants, such as nitrogen oxides, understanding the potential impact of contaminants on carbon dioxide electrolysis is crucial for practical applications. Herein, we investigate the impact of various nitrogen oxides, including nitric oxide, nitrogen dioxide, and nitrous oxide, on carbon dioxide electroreduction on three model electrocatalysts (i.e., copper, silver, and tin). We demonstrate that the presence of nitrogen oxides (up to 0.83%) in the carbon dioxide feed leads to a considerable Faradaic efficiency loss in carbon dioxide electroreduction, which is caused by the preferential electroreduction of nitrogen oxides over carbon dioxide. The primary products of nitrogen oxides electroreduction include nitrous oxide, nitrogen, hydroxylamine, and ammonia. Despite the loss in Faradaic efficiency, the electrocatalysts exhibit similar carbon dioxide reduction performances once a pure carbon dioxide feed is restored, indicating a negligible long-term impact of nitrogen oxides on the catalytic properties of the model catalysts.

## Abstract (web summary)

Understanding the impact of nitrogen oxides, common contaminants in CO_2_ emission from industries is a key to commercializing CO_2_ electrolysis technologies. Here, the authors conduct an investigation of CO_2_ electroreduction on copper, silver, and tin catalysts in the presence of nitric oxide, nitrogen dioxide, or nitrous oxide.

## Introduction

**Fig. 1:**
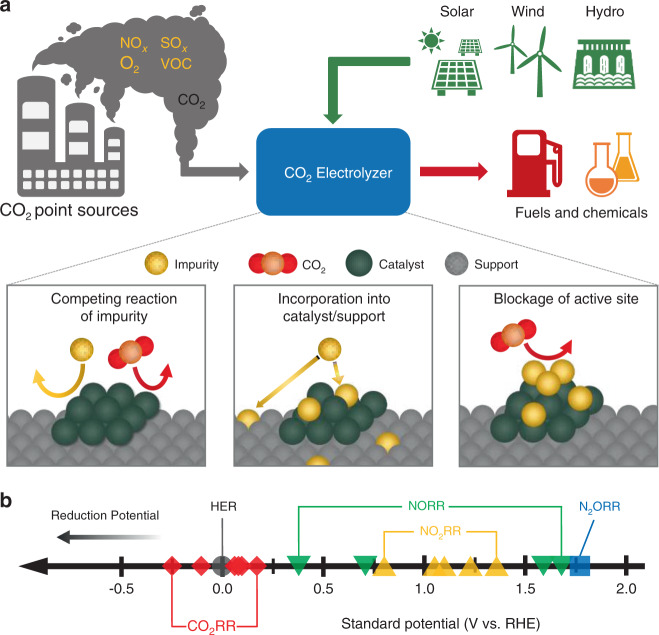
CO_2_ electrolysis technology using industrial CO_2_ point sources. (**a**) Schematics of CO_2_ electrolysis with CO_2_ stream obtained from point sources containing impurities such as nitrogen oxides (NO_x_), sulfur oxides (SO_x_), O_2_, and volatile organic compounds (VOC) and potential influence of impurities in CO_2_ electroreduction (CO_2_RR). (**b**) Standard potential vs. reversible hydrogen electrode (RHE) for CO_2_RR, hydrogen evolution reaction (HER), NO_2_ reduction (NO_2_RR), NO reduction (NORR), and N_2_O reduction (N_2_ORR). Detailed reactions are provided in Supplementary Table 1.

The electrochemical CO_2_ reduction (CO_2_RR) provides a promising, sustainable avenue to generate value-added fuels and chemicals from greenhouse gas CO_2_^1,2^. Depending on the choice of electrocatalyst, CO_2_ can be converted into a variety of single-carbon (C_1_; e.g., carbon monoxide, formic acid, methanol, and methane) and multi-carbon (C_2+_; e.g., ethylene, ethanol, acetate, and n-propanol) products with tremendous market potentials^3–8^. While CO_2_RR is being actively studied, most studies are conducted using highly pure CO_2_ feed^9,10^. For commercial applications, the most commonly available CO_2_ sources are industrial point sources, such as chemical and power plants;^11^ however, CO_2_ gas emitted from these sources often contain a variety of contaminants, such as sulfur oxides (SO_x_), nitrogen oxides (NO_x_), O_2_, and volatile organic compounds (VOC) (Fig. 1a)^12–14^. Therefore, there is an urgent need to understand the potential impact of common contaminants in industrial CO_2_ sources on the catalyst properties in CO_2_RR.

Gas impurity in CO_2_ can affect the performance of CO_2_RR electrocatalysts as we demonstrated in the case of SO_2_^15^_,_ where a trace amount of SO_2_ in the feed is sufficient to alter the product selectivity of Cu catalyst substantially. The potential impacts of impurity include lowering Faradaic efficiency (FE; i.e., number of electrons transferred to desired products divided by the total number of electrons passed in the system) due to competing reactions of impurity over CO_2_, altering the property of the catalyst by incorporating into the catalyst and/or support, and adsorbing on the catalyst surface to physically block the active sites (Fig. 1a). To date, there are only a few studies focusing on understanding how the presence of contaminants influences the behavior of electrocatalysts under CO_2_RR conditions^15–21^. For example, NO_x_ is one of the major contaminants present in industrial CO_2_ point sources with a typical concentration of 1000 ppm^12–14^. The NO_x_ contaminants typically consist of 90–95% nitric oxide (NO) and 5–10% nitrogen dioxide (NO_2_)^22^. Additionally, nitrous oxide (N_2_O) is also a common byproduct formed in the NO_x_ removal process^23^, which has a relatively low reactivity in comparison to other NO_x_. A previous study has shown that 200 ppm of NO has a negligible influence on Cu catalysts in CO_2_RR in a conventional batch cell^18^. Furthermore, less than or equal to 1667 ppm of NO_2_ has shown to be either beneficial or neutral, and greater than 1667 ppm of NO_2_ has shown to be detrimental in CO_2_RR, mainly due to a reduction in pH of the electrolyte, also on Cu catalysts in a conventional batch cell^19^. However, the behavior of various NO_x_ impurities in CO_2_RR at industrially relevant high current densities (>100 mA cm^−2^) has not been explored yet.

In this work, we investigate the influence of NO_x_ (i.e., NO, NO_2_, and N_2_O) in CO_2_RR using a three-compartment flow cell. Three model electrocatalysts, including copper (Cu), silver (Ag), and tin (Sn), are selected to represent the most studied catalysts for C_2+_ products, carbon monoxide (CO), and formate, respectively. Most NO_x_ contaminants in the CO_2_ feed significantly reduce the CO_2_RR FE because the electrochemical reduction of NO_x_ occurs at much more positive potentials than CO_2_RR (Fig. 1b). NO and NO_2_ impurities have more severe impacts on CO_2_RR FE than N_2_O, likely due to the greater number of electrons required in the NO_x_ reactions. Despite the loss of CO_2_RR FE, none of the three catalysts exhibits a significant change of product selectivity after removing the NO_x_ impurity from the CO_2_ feed. Moreover, we employ gas chromatography (GC), spectrophotometry, and flow electrochemical mass spectrometry (FEMS) to analyze the products of electroreduction of NO, the dominant component of NO_x_ in industrial point sources, in which the major products are ammonia (NH_3_), hydroxylamine (NH_2_OH), N_2_, and N_2_O. Investigation of the effect of different concentrations of NO in CO_2_RR shows that NO_x_ at typical concentrations in flue gases is compatible with CO_2_RR.

## Results and Discussion

Electrodes were prepared by loading commercial Cu, Ag, and Sn particles on a gas diffusion layer (GDL), a microporous carbon paper which provides mechanical support, electrical conductivity, and hydrophobicity. Scanning electron microscopy (SEM) images of the as-prepared electrodes confirm a uniform deposition of metal nanoparticles on GDL, covering the majority of the GDL surface (Supplementary Fig. 1). Electrochemical experiment was performed in a three-compartment flow cell, in which CO_2_ gas is directly fed to the electrode-electrolyte interface, enabling CO_2_RR at high current densities (Supplementary Fig. 2). NO_x_ impurities were mixed with CO_2_ gas feed prior to entering the flow cell. As the concentration of NO_x_ in typical exhaust streams may be as high as ~3,000 ppm (i.e., 0.3 vol. %)^13^, conservative streams of 83.3% CO_2_, 15.87% Ar, and 0.83% NO_x_ were used for most studies. To keep the CO_2_ partial pressure constant during the introduction of NO_x_, which contains Ar, CO_2_ partial pressure was maintained at 0.833 bar throughout the study by using a mixture of 83.3% CO_2_ and 16.7% Ar when NO_x_ was not introduced.

### Impact of NO_x_ impurities on CO_2_ electroreduction

**Fig. 2:**
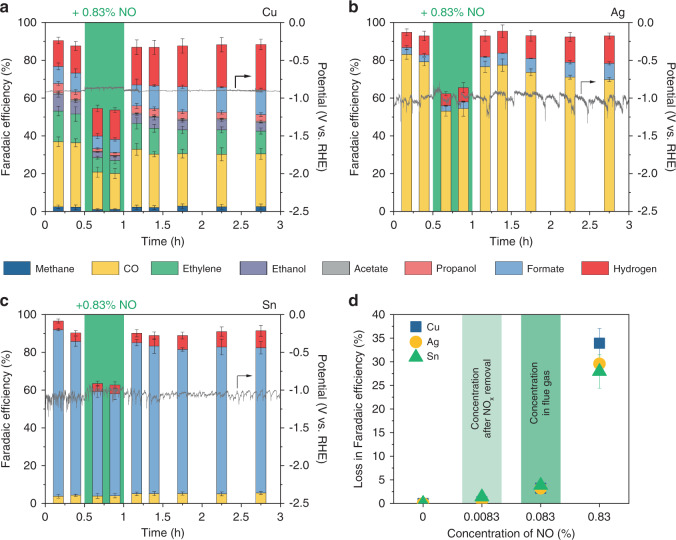
CO_2_ electroreduction performance in the presence of NO. Faradaic efficiency and applied potential vs. time on (**a**) Cu, (**b**) Ag, and (**c**) Sn catalysts at a constant current density of 100 mA cm^−2^ in 1 M KHCO_3_ for 3 h. Gas feeds were 83.3% CO_2_ and 16.7% Ar, and 83.3% CO_2_, 15.87% Ar, and 0.83% NO (green). 0.83% NO was introduced at 0.5 h for 0.5 h. Corresponding Faradaic efficiencies are provided in Supplementary Tables 2–4. (**d**) Effect of different concentrations of NO in CO_2_ electroreduction on Cu, Ag, and Sn catalysts. 0.083% and 0.0083% represent the typical NO_x_ concentrations in flue gases and flue gases after NO_x_ removal processes, respectively. Corresponding Faradaic efficiencies are provided in Supplementary Table 6. Error bars represent the standard deviation of three independent measurements.

The influence of NO in CO_2_RR on Cu, Ag, and Sn catalysts was first evaluated at a constant current density of 100 mA cm^−2^ (Fig. 2a–c). The CO_2_RR experiment was performed by switching the gas feed from 83.3% CO_2_ and 16.7% Ar (0–0.5 h) to 83.3% CO_2,_ 15.87% Ar, and 0.83% NO (green region; 0.5–1 h) and back to 83.3% CO_2_ and 16.7% Ar (1–3 h). With 83.3% CO_2_ and 16.7% Ar, before exposure to NO, Cu catalyst produced a wide range of C_1_ (i.e., methane, CO, and formate) and C_2+_ (i.e., ethylene, ethanol, acetate, and propanol) products. In the cases of Ag and Sn catalysts, the major products were CO and formate, respectively. The observed CO_2_RR selectivity of the Cu, Ag, and Sn catalysts was consistent with the previous reports^6,7^.

When 0.83% NO was introduced at *t* = 0.5 h, the total CO_2_RR FE decreased noticeably on all three catalysts (Fig. 2a–c). On average, the losses in CO_2_RR FE accounted for 33.9, 29.6, and 27.9% on Cu, Ag, and Sn, respectively (Fig. 2d), which is likely due to the preferential reduction of NO over CO_2_. Assuming NO is fully converted to NH_3_, conversions of NO during CO_2_RR are between 48% and 60% (Supplementary Table 5). As shown in Fig. 1b, the standard potentials of NORR are much more positive than those of CO_2_RR. For instance, the standard potential of NORR to N_2_ is 1.68 V vs. RHE, while the standard potentials of CO_2_RR are between −0.250 and 0.169 V vs. RHE. Cyclic voltammetry (CV) measurements under CO_2_ with 0.83% NO also confirmed that NORR is more favorable than CO_2_RR on Cu, Ag, and Sn catalysts (Supplementary Fig. 3). On all three catalysts, onset potentials and cathodic currents shifted to more positive potentials when 0.83% NO was introduced to the CO_2_ stream. CV measurements under different concentrations of NO in Ar also confirmed more positive onset potentials of NORR than CO_2_RR and showed that NORR at 0.83% NO is mass transport limited (Supplementary Fig. 4). After restoring 83.3% CO_2_ and 16.7% Ar, the CO_2_RR performance and the total CO_2_RR FE on all three catalysts quickly recovered and were stable for additional 2 h of electrolysis. No obvious change in selectivity was observed for any of the three catalysts, suggesting that the exposure to NO did not alter the catalyst property in any significant way. There is a slight increase in H_2_ FE over time (Fig. 2a–c), but it is likely due to the slow flooding of the electrode (Supplementary Fig. 5)^24^.

To obtain insight on the influence of NO_x_ in CO_2_RR at typical concentrations of NO_x_ in point sources, we evaluated the effect of 0.083% and 0.0083% NO, representing the typical NO_x_ concentrations in flue gases and flue gases after NO_x_ removal processes^22^, respectively, in CO_2_RR (Fig. 2d). Although the losses in FE at 0.83% NO were detrimental, the effect of NO was less severe at 0.083%, with less than 5% losses in FE, and negligible at 0.0083% NO. Therefore, NO at typical concentrations of NO_x_ in flue gases is compatible with CO_2_RR, although complete removal of NO_x_ is desired to maximize CO_2_RR FE.

**Fig. 3:**
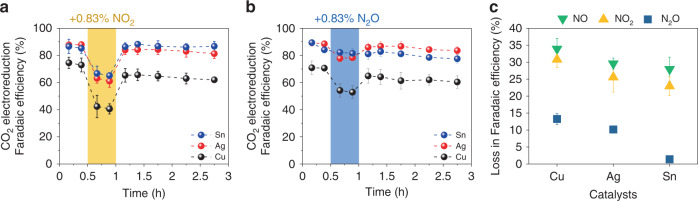
CO_2_ electroreduction performance in the presence of NO_2_ and N_2_O. CO_2_ electroreduction Faradaic efficiency, excluding hydrogen Faradaic efficiency, vs. time with the introduction of (**a**) 0.83% NO_2_ (yellow) and (**b**) 0.83% N_2_O (blue) on Cu, Ag, and Sn catalysts at a constant current density of 100 mA cm^−2^ in 1 M KHCO_3_ for 3 h. Gas feeds were 83.3% CO_2_ and 16.7% Ar, and 83.3% CO_2_ and 15.87% Ar with 0.83% NO_2_ or 0.83% N_2_O. NO_2_ and N_2_O were introduced at *t* = 0.5 h for 0.5 h. Corresponding Faradaic efficiencies are provided in Supplementary Figs. 6 and 7, and Supplementary Tables 7–12. (**c**) Loss in Faradaic efficiency during CO_2_ electroreduction from the introduction of 0.83% NO, 0.83% NO_2_, and 0.83% N_2_O on Cu, Ag, and Sn catalysts. Corresponding Faradaic efficiencies are provided in Supplementary Table 13. Error bars represent the standard deviation of three independent measurements.

NO_2_ is another major contaminant in industrial CO_2_ point sources (5–10% of NO_x_), and a substantial amount of N_2_O may also be formed as a byproduct during the NO_x_ removal process^22,23^. Thus, we further investigated the influence of NO_2_ and N_2_O in CO_2_RR on Cu, Ag, and Sn catalysts following the similar experimental procedure to the NO experiment. When 0.83% NO_2_ was introduced at *t* = 0.5 h (yellow region), the CO_2_RR FE decreased on all three catalysts (Fig. 3a). The decrease in the total FEs were 30.8, 25.6, and 22.9% on Cu, Ag, and Sn catalysts, respectively. Similarly, when 0.83% N_2_O was introduced (blue region), the total CO_2_RR FE decreased by 11.4, 10.2, and 1.4% on Cu, Ag, and Sn catalysts, respectively (Fig. 3b). Distinct from Cu and Ag catalysts, Sn catalyst did not show a significant loss of the CO_2_RR FE in the presence of N_2_O, which is likely due to the poor activity of Sn for N_2_ORR^25^. Sn catalyst maintained a high CO_2_RR FE over the course of 3 h of electrolysis, suggesting the resistive feature of Sn catalyst to N_2_O impurity. As shown in Fig. 1b, standard potentials of NO_2_ and N_2_O are also more positive than those of CO_2_RR, and therefore, we attribute the loss of the CO_2_RR FE to the preferential reduction of NO_2_ and N_2_O over CO_2_, which is further supported by the CV study (Supplementary Figs. 8–11). When a pure CO_2_ feed was restored, the total CO_2_RR FE on all three catalysts quickly recovered, suggesting that the exposure of NO_2_ and N_2_O does not affect the property of the catalysts.

A comparison of the losses in FE due to the various NO_x_ impurities is presented in Fig. 3c. NO and NO_2_ show greater losses in FE than N_2_O on all three catalysts, likely due to the greater number of electrons required in the reactions. As will be discussed in the following section, the main products of NORR are NH_3_ and NH_2_OH, which require 5 and 3 electrons, respectively, while the main product of N_2_ORR is N_2_, which only requires 2 electrons. Given that all NO_x_ readily reacts at the catalyst surface, the same amount of NO and NO_2_ consume more electrons than N_2_O, causing greater losses in CO_2_RR FE. Among all the catalysts, the Cu catalyst suffers the largest FE loss on all NO_x_ impurities, followed by Ag and Sn catalysts. Indeed, Cu has been demonstrated as one of the more active metals for the electroreduction of NO^26^ and N_2_O^25^, in which Cu achieved high FE in N_2_ORR to N_2_ at relatively low overpotentials. The results suggest that Cu is an effective electrocatalyst for NO_x_ reduction, which may be further explored in future studies.

Furthermore, pH was measured at the outlet of the electrolyzer at different time points (i.e., before, during, and after NO_x_ introduction) to investigate the effect of NO_x_ on the electrolyte pH (Supplementary Fig. 12). The measured pH shows that the presence of NO and N_2_O has a negligible effect on the pH, while the presence of NO_2_ slightly decreases the pH by 0.03. Although NO_2_ hydrolyzes to produce nitric acid and nitrous acid^27^, the effect in pH is very small, possibly due to the small amount of NO_2_ in the gas feed, rapid reaction of NO_2_ at the catalyst surface which prevents NO_2_ from penetrating to the bulk electrolyte, and a flowing electrolyte which is constantly replenished.

### Identification of NO_x_ reduction products

**Fig. 4:**
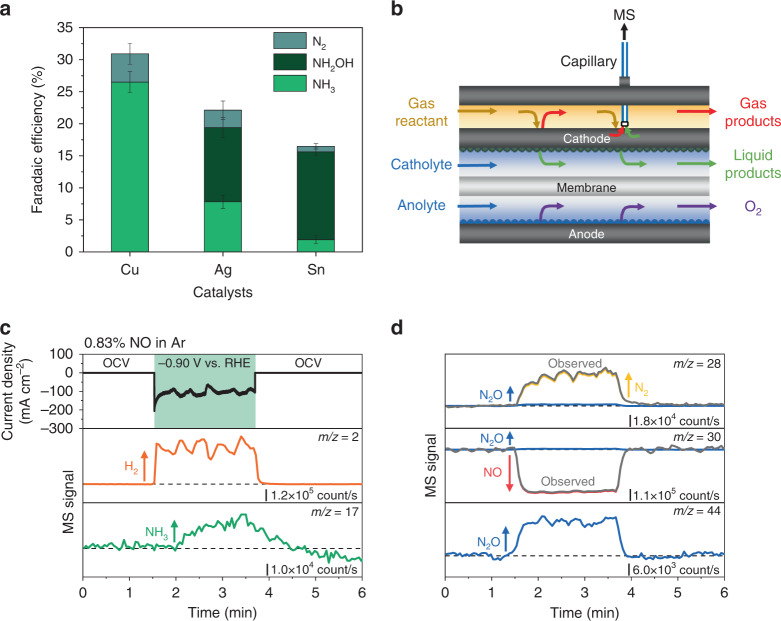
Investigation of the NO electroreduction products. (**a**) Faradaic efficiency of NO electroreduction products produced during electrolysis with 83.3% CO_2_, 15.87% Ar, and 0.83% NO on Cu, Ag, and Sn catalysts at a constant current density of 100 mA cm^−2^ in 1 M KHCO_3_ for 3 h. Corresponding Faradaic efficiencies are provided in Supplementary Table 14. Error bars represent the standard deviation of three independent measurements. (**b**) Schematic of flow electrochemical mass spectrometry (FEMS) setup. (**c**) Measured current density vs. time, and deconvoluted MS signal vs. time for *m/z* = 2, *m/z* = 17, **(d**) *m/z* = 28, *m/z* = 30, and *m/z* = 44 from FEMS on Cu catalyst in 1 M KHCO_3_ with 0.83% NO in Ar. −0.90 V vs. RHE was applied for approximately 2 min starting at *t* = 1.5 min. NORR products have been deconvoluted using the mass spectra of individual products shown in Supplementary Fig. 17. Additional information is provided in the Methods section and Supplementary Figs. 18 and 19.

The electrochemical reduction products of NO, the major component of NO_x_ in industrial point sources, were further investigated. As NH_3_, NH_2_OH, N_2_, and N_2_O have been suggested as the main products in NORR^26,28,29^, NH_3_ and NH_2_OH were detected via spectrophotometry (Supplementary Figs. 13 and 14), and N_2_ was detected via GC (Supplementary Fig. 15). We note that the concentration of N_2_O in the gas product stream was below the detection limit of GC, suggesting that N_2_O FE was below 2% FE on all three catalysts. As shown in Fig. 4a, NORR product selectivity varied among different catalysts. Cu primarily produced NH_3_ and N_2_, with no NH_2_OH, Ag produced a mixture of NORR products, and Sn primarily produced NH_2_OH. These observations are consistent with previous reports, in which Cu has been demonstrated as an effective catalyst for NORR to NH_3_^26^, and Sn has been used as a dopant in Pt to shift the selectivity from NH_3_ to NH_2_OH in nitrate reduction^30^.

To further probe the formation of NORR products with greater sensitivity and determine the formation of N_2_O, we employed the FEMS (Fig. 4b and Supplementary Fig. 16), which allows us to continuously measure gas and volatile liquid products operando with a low detection limit and a short response time by continuously pulling products to the mass spectrometry (MS) near the surface of the electrodes (See Methods for more details). The MS probe was placed near the working electrode from the gas channel side, and the MS signals linked to possible products were tracked over time. We conducted the FEMS measurement on the Cu catalyst using 0.83% NO in Ar (in the absence of CO_2_), because the ionization of N_2_ (*m/z* = 28, 14) and N_2_O (*m/z* = 44, 30, 28, 14) produces the same fragments with CO_2_ and various CO_2_ reduction products^31–33^, complicating the reliable analysis of the NORR products (see Supplementary Note for more details). MS signals of the FEMS measurement under a continuous feed of 0.83% NO are presented in Fig. 4c, d. When a constant potential of −0.90 V vs. RHE was applied at *t* = 1.5 for ~2 min, MS signals of NO (*m/z* = 30) decreased while those of H_2_ (*m/z* = 2), NH_3_ (*m/z* = 17), N_2_ (*m/z* = 28) and N_2_O (*m/z* = 44) increased (Fig. 4c, d), indicating the consumption of NO and the formation of H_2_, NH_3_, N_2_, and N_2_O. The formation of NH_3_ and N_2_ detected by FEMS is in agreement with the results obtained from spectrophotometry and GC analysis, respectively. The production of N_2_O, which was difficult to measure via GC, was clearly observed in FEMS, suggesting that N_2_O is one of the NORR products. NH_2_OH was not detected in FEMS, because it is nonvolatile^30^. Similarly, FEMS results also suggest the formation of N_2_ and N_2_O on Ag and Sn catalysts (Supplementary Figs. 20–25). However, the formation of NH_3_ was observed only on Ag and not on Sn, likely due to the small amount of NH_3_ produced on Sn. Collectively, NH_3_, NH_2_OH, N_2_, and N_2_O have been determined as the NORR products. The analysis of the NORR products further confirms that the loss in CO_2_RR FE is due to the preferential reduction of NO over CO_2_.

In the case of N_2_ORR, a substantial amount of N_2_ was quantified with a GC (Supplementary Fig. 26). While the losses of CO_2_RR FE were 11.4%, 10.2%, and 1.4% on Cu, Ag, and Sn catalysts, respectively, the amounts of N_2_ detected were 8.2%, 7.3%, and 0.5% of the total FE, respectively, accounting for the majority of the loss in the CO_2_RR FE. Small amount of N_2_ detected on Sn catalyst demonstrates the resistive nature of Sn catalyst in N_2_ORR.

### Characterization of catalyst structures in the presence of NO_x_

**Fig. 5:**
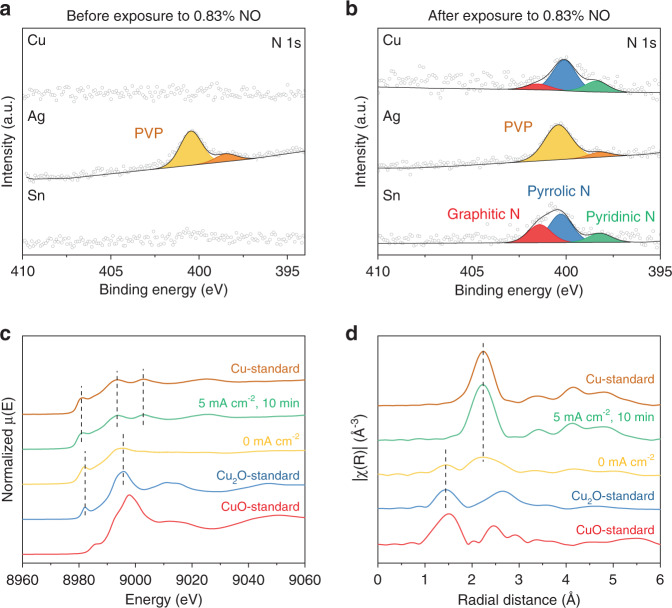
Evaluation of the influence of NO on the catalyst structure. XPS measurements of Cu, Ag, and Sn electrodes (**a**) before (*t* = 0 h) and (**b**) after exposure to 0.83% NO (*t* = 1 h) during CO_2_ electrolysis. Corresponding XPS data is provided in Supplementary Fig. 27 and Supplementary Table 15. Cu K-edge (**c**) XANES and (**d**) EXAFS spectra of spent Cu catalyst after exposure to 0.83% NO during CO_2_ electrolysis. Cu foil, Cu_2_O, and CuO were used as references.

X-ray photoelectron spectroscopy (XPS) measurements were conducted to reveal the influence of NO_x_ on the surface electronic structure and the chemical environment of the catalysts. The samples were obtained at various points of the CO_2_RR experiment, including before exposure to NO_x_, after exposure to NO_x_, and at the end of 3-h electrolysis. As shown in Fig. 5a, the Cu and Sn electrodes before the exposure to NO_x_ did not show any noticeable peak in N 1 s XPS measurements. In contrast, Ag showed two distinct peaks at 400.5 eV and 398.5 eV, which can be attributed to polyvinylpyrrolidone (PVP)^34,35^, a surfactant used in the nanoparticle synthesis. The XPS measurements obtained after the NO exposure (*t* = 1 h) exhibited new N 1 s peaks on Cu and Sn electrodes (Fig. 5b). The peaks at 401.4 eV, 400.2 eV, and 398.2 eV can be assigned to graphitic, pyrrolic, and pyridinic N, respectively^36,37^, suggesting that incorporated N atoms mainly interact with carbon in GDL rather than metal catalysts (metal nitride peaks typically observed near 397 eV)^38,39^. The XPS measurements obtained after 3-h electrolysis show that the N incorporated in the electrode surface was still intact after additional 2 h of CO_2_RR (Supplementary Fig. 27 and Supplementary Table 15), with the total amount of N in the Cu and Sn electrodes remaining relatively unchanged. In the cases of 0.83% NO_2_ and 0.83% N_2_O, the XPS measurements show similar N incorporation in GDL (Supplementary Figs. 28 and 29, and Supplementary Tables 16 and 17). Regarding the Ag electrode, the XPS investigation of N incorporation associated with NO_x_ was largely limited by the presence of the PVP surfactant.

To further confirm the incorporation of N into GDL rather than the formation of metal nitrides, we increased the catalyst loading to 2.0 mg cm^−2^, which created a thick layer of catalyst on the GDL with much less exposure of GDL in the XPS measurement. After exposure to 0.83% NO during CO_2_ electrolysis, the N 1 s signal was not detected on the Cu and Sn electrodes with the increased catalyst loading, whereas the XPS measurements for the Ag electrode clearly shows the N 1 s signal, which is due to the presence of PVP on the surface of Ag catalyst (Supplementary Fig. 30). Conversely, when the same experiment was repeated with GDL without any catalyst, N species was still detected, confirming the incorporation of N into GDL (Supplementary Fig. 30). Experiments using NO_2_ and N_2_O show similar incorporation of N into GDL (Supplementary Fig. 30).

To probe the influence of NO_x_ impurity on the oxidation state of the Cu catalyst, we conducted X-ray absorption spectroscopy (XAS) measurements using a customized XAS batch cell (Supplementary Fig. 31). Because of the toxicity of the NO_x_ gases, we did not use the NO_x_ gases directly at the synchrotron X-ray beamline but conducted XAS experiments with the electrodes taken out of the electrolyzer at 1 h (after exposure to NO_x_ for 0.5 h) during CO_2_ + NO_x_ experiments (Fig. 2a-c and Supplementary Figs. 6 and 7). The Cu K-edge X-ray absorption near-edge spectroscopy (XANES) spectra of the Cu catalyst after the NO exposure show a similar spectrum of the Cu_2_O standard, suggesting an average Cu oxidation state of +1 (Fig. 5c). Extended X-ray absorption fine structure (EXAFS) result shows that the NO-exposed Cu sample contains a mixture of Cu and Cu_2_O (Fig. 5d). Slight oxidation of Cu is likely due to the exposure of the sample in the air during sample handling. After a constant current density of 5 mA cm^−2^ was applied under CO_2_RR condition, the Cu catalyst was quickly reduced to metallic Cu, suggesting that a small amount of current is sufficient to fully reduce the Cu catalyst under CO_2_RR conditions. XAS measurements on Cu samples exposed to NO_2_ and N_2_O also exhibited similar behaviors as the NO-treated Cu sample (Supplementary Figs. 32 and 33), confirming that the Cu catalyst remains or revert to fully metallic under reaction conditions after NO_x_ is removed from the CO_2_ stream.

Moreover, ex-situ SEM images were obtained at various points of the experiment to evaluate the impact of NO_x_ on the catalyst morphology. SEM images of the spent catalysts after the exposure to NO_x_ impurities (*t* = 1 h and 3 h) exhibit minimal changes in Cu and Ag catalysts (Supplementary Figs. 1, 34, and 35). Although an increase in particle size was observed in the case of Sn catalysts (Supplementary Figs. 1 and 36), the Sn sample obtained after 1 h of CO_2_ electrolysis in the absence of NO_x_ also showed a similar increase in particle size (Supplementary Fig. 37). The Sn particles likely aggregated to lower the surface energy under CO_2_RR condition regardless of NO_x_, and therefore, NO_x_ impurities are not the primary cause of the size change of the Sn particles during CO_2_RR. These results suggest that the presence of NO_x_ during CO_2_RR has a negligible impact on the catalyst morphology.

## Conclusions

In summary, we investigated the influence of various NO_x_ (i.e., NO, NO_2_, and N_2_O) in CO_2_RR on Cu, Ag, and Sn catalysts in a flow cell. The presence of NO_x_ impurities reduced the CO_2_RR FE due to the preferential reduction of NO_x_ over CO_2_. The impact of NO and NO_2_ is more severe than that of N_2_O in CO_2_RR due to the greater number electrons involved in NORR and NO_2_RR compared to N_2_ORR. The major NORR products are NH_3_, NH_2_OH, N_2_, and N_2_O, in which the selectivity varies among different catalysts, whereas N_2_O is primarily reduced to N_2_. Despite the loss of CO_2_RR FE, a small amount of NO_x_ in the CO_2_ feed does not alter the metallic nature of the catalyst under CO_2_RR conditions as demonstrated by the XPS and XAS measurements. Furthermore, although high concentrations of NO_x_ may be detrimental to CO_2_RR, NO_x_ at typical concentrations of flue gases is compatible with CO_2_RR, causing small losses in CO_2_RR FE. NO_x_ removal process, which is a relatively mature technology, may also be employed to ensure CO_2_RR operation at maximum efficiency. This work not only demonstrates the effect of a trace amount of NO_x_ impurities that are often present in the industrial CO_2_ point sources on the most commonly studied metal catalysts, but also offers new insights on the electrochemical reduction of NO_x_, which has rarely been explored in the literature.

## Methods

### Electrode preparation

Commercial Cu (25 nm, Sigma-Aldrich), Ag (<100 nm, 99.5%, Sigma-Aldrich), and Sn (0.1 μm, Alfa Aesar) particles were used as cathode catalysts. Commercial IrO_2_ (99.99%, Alfa Aesar) was used as an anode catalyst. The catalyst inks were prepared by dissolving 3 mg of the catalyst and 20 μl of Nafion (5 weight % in 50/50 water and isopropanol) in 3 mL of isopropanol. The catalyst ink was sonicated for at least 30 min, and 0.25 mg cm^−2^ of the catalyst was drop casted onto a Sigracet 29 BC GDL (Fuel Cell Store).

### Flow cell electrolysis

The electrochemical measurements were conducted in a three-compartment flow cell with channel dimensions of 2 cm by 0.5 cm by 0.15 cm (Supplementary Fig. 2). The electrode area was 1 cm^2^ and the distance between the electrode and the membrane was 0.15 cm. A FAA-3-hydroxide exchange membrane (Fumatech) was used to separate electrolyte in the anode and the cathode chamber. 1 M KHCO_3_ was prepared by purging CO_2_ (Matheson, 99.999%) into potassium carbonate (99%, Alfa Aesar) and purified using a Chelex 100 sodium salt (Sigma Aldrich). After filtering Chelex 100 sodium salt, 1 M KHCO_3_ was used as an electrolyte for both catholyte and anolyte and was fed at 0.9 mL min^−1^ via peristaltic pumps (Cole Parmer). The total gas flow rate was maintained at 19.2 mL min^−1^ with different flow rates of CO_2_, Ar (Keengas, 99.999%), and NO_x_. For instance, 83.3% CO_2_ and 16.7% Ar was prepared by flowing 16 mL min^−1^ CO_2_ and 3.2 mL min^−1^ Ar via Brooks GF40 mass flow controllers. 83.3% CO_2_, 15.87% Ar, and 0.83% NO_x_ were prepared by flowing 16 mL min^−1^ CO_2_ with 3.2 mL min^−1^ of 5% NO/Ar (Matheson Gas) or 3.2 mL min^−1^ of 5% NO_2_/Ar (Matheson Gas) using a 50 mL gastight syringe (1050 SL, Hamilton) via a syringe pump (New Era Pump Systems). Syringes were quickly switched to another syringe before running out of gases. Similarly, 83.3% CO_2_, 15.87% Ar, and 0.83% N_2_O were prepared by flowing 16 mL min^−1^ CO_2_, 3.04 mL min^−1^ Ar, and 0.16 mL min^−1^ N_2_O (99.99%, Matheson Gas). N_2_O was fed by using a 10 mL gastight syringe (1010 SL, Hamilton) via a syringe pump (Cole Parmer). For NO_2_ experiment, the gas outlet of the electrolyzer was connected to 2 M KOH (85%, Sigma-Aldrich) to scrub the remaining NO_2_ and additional Ar was flowed at 16 mL min^−1^ to carry the CO_2_RR products to the GC.

CV and chronopotentiometry experiments were conducted via an Autolab PG128N. For CV measurements, the electrodes were pre-reduced at 100 mA cm^−2^ in 83.3% CO_2_ and 16.7% Ar for 10 min. The half-cell potentials were measured with respect to Ag/AgCl reference electrode (Pine Research) and calculated to the RHE scale in which E (vs. RHE) = E (vs. Ag/AgCl) + 0.209 V + 0.0591 V × pH − *η*_*IRdrop*_. The pH was measured at the outlet of the catholyte channel. The resistance was measured with the current-interrupt technique^40^, and the measured potential was manually post IR-corrected.

### Product quantification

The gas products were analyzed via a multiple gas analyzer no. 5 gas chromatography system (SRI Instruments) equipped with a Molsieve 5 A and a HayeSep D column connected to a thermal conductivity detector (TCD) and a flame ionization detector (FID). Ar was used as a carrier gas with a flow rate of 19 mL min^−1^ and 1 mL of sample was automatically loaded to the column. The gas sample was loaded to 0.5 m HaySep D pre-column connected to 2 m Molsieve 5 A column at 0.050 min. At 0.490 min, any molecule remaining in the HaySep D precolumn was backflushed out to vent. At 2.150 min, the gas sample was automatically loaded to 2 m HaySep D column. The column temperature was maintained at 35 °C for 2.950 min, increased to 210 °C at 40 °C/min, and maintained at 210 °C until the end of the analysis. A typical GC analyses of potential CO_2_RR products, N_2_, NO, and N_2_O are provided in Supplementary Fig. 38. 2% H_2_, 1% CO, 1% CH_4_, 1% C_2_H_4_, 0.50% C_2_H_6_, 0.25% C_3_H_6_, 0.25% C_3_H_8_ in Ar (Matheson) was used to obtain the chromatogram of potential CO_2_RR products.

The liquid CO_2_RR products were analyzed via ^1^H nuclear magnetic resonance (NMR) with water suppression using a presaturation method (Bruker AVIII 600 MHz NMR spectrometer). The liquid sample was collected at the outlet of the electrolyzer and diluted to 25% in deionized water (DI). 500 μL of the diluted sample was mixed with 100 μL of 25 ppm (volume %) dimethyl sulfoxide (99.9%, Alfa Aesar), which was used as an internal standard, in D_2_O.

NH_3_ was quantified using indophenol blue method^41^ with UV-vis spectroscopy (Nanodrop 2000, Thermo Scientific). 100 μL of the sample was mixed with 500 μL of alkaline hypochlorite solution (A1727, Sigma-Aldrich) and 500 μL of phenol nitroprusside solution (P6994, Sigma-Aldrich). The solution was incubated in the dark at room temperature for 20 min. 2 μL of the solution was pipetted onto the pedestal, and the absorbance was measured by UV–vis spectroscopy from 190 nm to 840 nm. The absorbance of the sample was measured at 630 nm, and the absorbance measured at 830 nm was subtracted to remove the background. The calibration curves were obtained using different concentrations of ammounium hydroxide (NH_4_OH; 28.0–30.0%, Sigma Aldrich) in 0.25 M KHCO_3_ (Supplementary Fig. 13).

NH_2_OH was quantified using a procedure modified from a procedure reported by Afkhami et al^42^. with UV–vis spectroscopy (Nanodrop 2000, Thermo Scientific). Neutral red solution was prepared by dissolving 200 mg of neutral red (Sigma-Aldrich) in 100 mL DI. Iodate solution was prepared by dissolving 1.00 g of potassium iodate (KIO_3_, 99.995%, Sigma-Aldrich) in 100 mL DI. A total of 500 μL of sample was mixed with 250 μL of 3.0 M sulfuric acid (Fisher Scientific) and 250 μL of iodate solution. After 5 min at room temperature, 500 μL of neutral red solution was added to the solution. The solution was incubated at room temperature for 20 min. In total 2 μL of the solution was pipetted onto the pedestal, and the absorbance was measured by UV–vis spectroscopy from 190 nm to 840 nm. The absorbance of the sample was measured at 510 nm, and the absorbance measured at 800 nm was subtracted to remove the background. The change in absorbance was determined by subtracting the absorbance of the sample solution from the absorbance of the solution with 0 mg L^−1^ NH_2_OH. The calibration curves were obtained using different concentrations of hydroxylamine (50 wt % in H_2_O, Sigma Aldrich) in 0.25 M KHCO_3_ (Supplementary Fig. 14).

### Flow electrochemical mass spectrometry (FEMS)

An identical flow cell with an entrance for the MS probe at the top of the gas channel was used for the FEMS measurement (Supplementary Fig. 16). The probe consisted of a PEEK capillary with inner diameter of 0.25 mm with PTFE membrane attached at the tip of the capillary. The PTFE membrane with a pore size of 200 μm was used to prevent the entry of aqueous electrolyte, while allowing gaseous and volatile products to enter the MS chamber. The distance between the probe and the cathode was kept constant. The electrodes were pre-reduced at 10 mA cm^−2^ for 5 min in Ar before the introduction of 0.83% NO. The products were detected by a Hiden Quadrupole mass spectrometer (MS). The mass fragments were detected by a secondary electron detection voltage of 1700 V with an ionization potential of 70 eV and emission current of 200 A. *m/z* of interest was tracked over the course of the experiment, in which a constant potential was applied for approximately 2 min starting at *t* = 1.5 min. For the deconvolution of *m/z* = 17 signal, *m/z* = 17 signal from the water was first determined using *m/z* = 18 signal. Next, the contribution from water to *m/z* = 17 signal was subtracted from the observed *m/z* = 17 signals to obtain the signal from ammonia. For the deconvolution of *m/z* = 28 and 44 signals, *m/z* = 28 and 44 signals from CO_2_ in the electrolyte was first determined using *m/z* = 12 signal. *m/z* = 12 signal was smoothed using the Savitzsky-Golay method with a window of 30 data points to reduce the oscillations in the signal prior to deconvolution. Next, the contributions from CO_2_ to *m/z* = 28 and 44 were subtracted from the observed *m/z* = 28 and 44 signals to obtain the signals from NORR products. *m/z* = 44 signal corresponded to the signal from N_2_O, and this was used to calculate the contribution of N_2_O to *m/z* = 28 and 30. Lastly, the contributions of N_2_O to *m/z* = 28 and 30 were subtracted from the *m/z* = 28 and 30 signals from NORR products, respectively, to yield N_2_ and NO signals, respectively. All deconvolution was conducted using MATLAB. Mass spectra of NH_4_OH, NO, N_2_O, N_2_, H_2_O, and CO_2_ used for the deconvolution were obtained using the same MS equipment (Supplementary Fig. 17).

### Material characterization

For SEM and XPS measurements, the electrodes were first taken out of the electrolyzer after electrolysis at desired time points. The electrodes were dried in the vacuum oven (MTI Corporation) for up to three days before SEM images were acquired with Auriga 60 CrossBeam (1.5 kV). The electrodes were quickly transported to the XPS equipment (K-alpha Alpha X-ray photoelectron spectrometer system, Thermo Fisher Scientific) after drying in the vacuum oven for 5 min. The electrodes were exposed to air for less than 20 min. High-resolution XPS measurements were obtained at pass energy of 20 eV with a step size of 0.1 eV. Flood gun was turned on. Cu 2p, Ag 3d, and Sn 3d were scanned 10 times while N 1 s was scanned 30 times. Four different spots were scanned and averaged. All peaks were fitted using Thermo Avantage software with adventitious carbon referenced to the C1s peak at 284.8 eV.

XAS measurement was performed at the 8-ID Beamline of the National Synchrotron Light Source II at Brookhaven National Laboratory (BNL). The electrodes were taken out of the electrolyzer at 1 h (after exposure to NO_x_ for 0.5 h) during a 100 mA cm^−1^ constant current CO_2_RR experiment with the introduction of NO_x_ (Fig. 2a–c and Supplementary Figs. 6 and 7). In the case of NO_2_, the samples were exposed to 0.23% NO_2_ instead due to the availability of the gas at the time of the experiment. The electrodes were quickly stored in vials filled with Ar and the vials were tightly sealed with Parafilm at the home institution. The electrodes were transported to the Brookhaven National Laboratory (New York, USA) and were loaded into a XAS batch cell, which was fabricated from Teflon and 304 stainless steel, with a Kapton film window for high transmissivity for X-ray measurements (Supplementary Fig. 31). The electrodes were exposed to air for ~20 min before the measurement. Pt wire and Ag/AgCl were used as a counter and a reference electrode, respectively. 1 M KHCO_3_ was used as an electrolyte and CO_2_ was flowed at 10 mL min^−1^. XAS data were analyzed using the IFEFFIT package, which included ATHENA and ARTEMIS^43^.

## Supplementary information

Supplementary Information

Peer Review

## Data Availability

All data needed to evaluate the conclusions in the paper are present in the paper and/or the Supplementary Materials. Additional data related to this paper may be requested from the authors.
