# Supplementary material for: The impact of nitrogen oxides on electrochemical carbon dioxide reduction
Source: Nat Commun. 2020 Nov 17;11:5856. doi: 10.1038/s41467-020-19731-8 (PMC7672067; doi:10.1038/s41467-020-19731-8)
Supplement: Supplementary file 1 — Supplementary Information [file 41467_2020_19731_MOESM1_ESM.pdf]

Supplementary Information

The impact of nitrogen oxides on electrochemical carbon dioxide reduction

Byung Hee Ko,[1] Bjorn Hasa,[1] Haeun Shin,[1] Emily Jeng,[1] Sean Overa,[1] Wilson Chen,[1] and Feng Jiao[1*]

[1] Center for Catalytic Science and Technology, Department of Chemical and Biomolecular Engineering, University of Delaware, Newark, DE, 19716 (USA)

*Corresponding author: jiao@udel.edu

Supplementary Table 1. Standard potentials for CO_2RR, HER, NO_2RR, NORR, and N_2ORR.[1–3]

	Reactions	Standard potential (V vs. RHE)
**CO_2RR**	$CO_2 + 2H^+ + 2e^- \leftrightarrow HCOOH$	-0.250
	$CO_2 + 2H^+ + 2e^- \leftrightarrow CO + H_2O$	-0.106
	$2CO_2 + 12H^+ + 12e^- \leftrightarrow C_2H_4 + 4H_2O$	0.064
	$2CO_2 + 12H^+ + 12e^- \leftrightarrow C_2H_5OH + 3H_2O$	0.084
	$3CO_2 + 18H^+ + 18e^- \leftrightarrow C_3H_7OH + 5H_2O$	0.095
	$CO_2 + 8H^+ + 8e^- \leftrightarrow CH_4 + H_2O$	0.169
**HER**	$2H^+ + 2e^- \leftrightarrow H_2$	0
**NO_2RR**	$NO_2 + 7H^+ + 7e^- \leftrightarrow NH_3 + 2H_2O$	0.80
	$NO_2 + 2H^+ + 2e^- \leftrightarrow NO + H_2O$	1.05
	$NO_2 + H^+ + e^- \leftrightarrow HNO_2$	1.10
	$2NO_2 + 6H^+ + 6e^- \leftrightarrow N_2O + 3H_2O$	1.23
	$2NO_2 + 8H^+ + 8e^- \leftrightarrow N_2 + 4H_2O$	1.36
**NORR**	$NO + 3H^+ + 3e^- \leftrightarrow NH_2OH$	0.38
	$NO + 5H^+ + 5e^- \leftrightarrow NH_3 + H_2O$	0.71
	$2NO + 2H^+ + 2e^- \leftrightarrow N_2O + H_2O$	1.59
	$2NO + 4H^+ + 4e^- \leftrightarrow N_2 + 2H_2O$	1.68
**N_2ORR**	$N_2O + 2H^+ + 2e^- \leftrightarrow N_2 + H_2O$	1.77

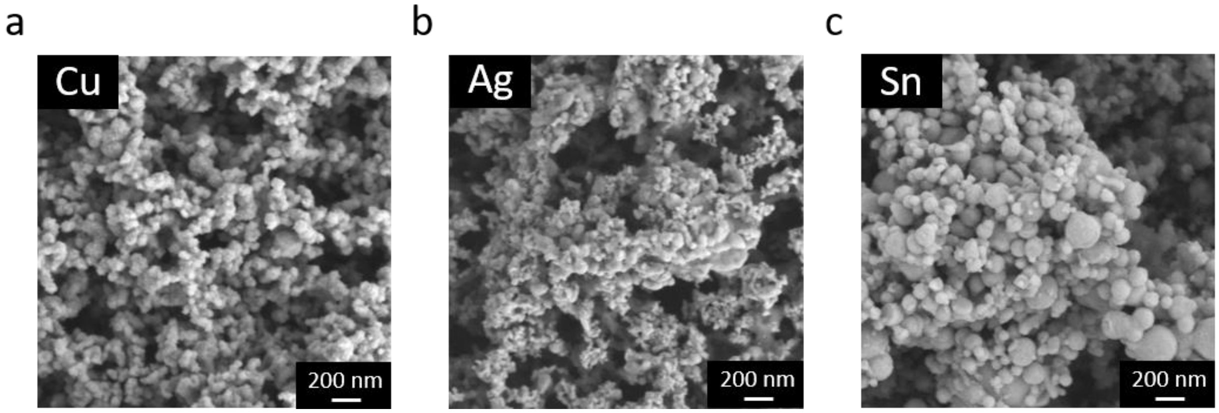

Supplementary Figure 1. SEM images of (a) Cu, (b) Ag, and (c) Sn electrodes before electrolysis. Metal particles are deposited uniformly on GDL.

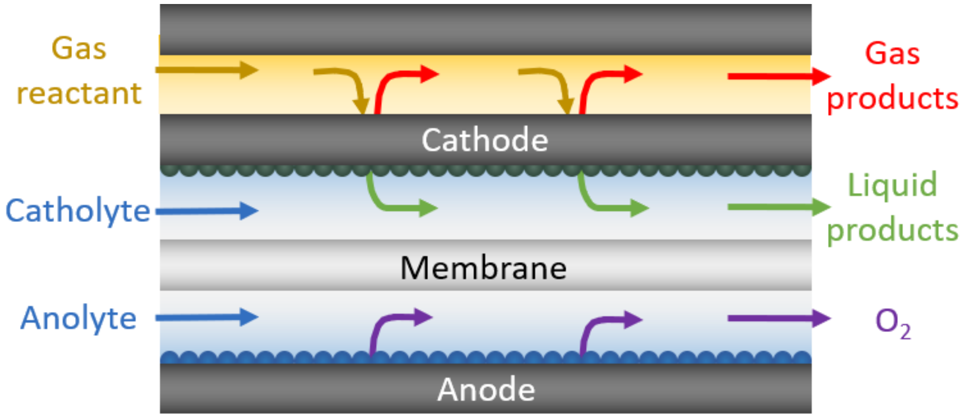

Supplementary Figure 2. Schematic of the three-compartment flow-cell. CO_2 gas is fed to the electrode-electrolyte interface without mass transport limitation, enabling CO_2RR at high current densities.

Supplementary Table 2. CO_2 electroreduction performance with the introduction of 0.83% NO impurity at 0.5 h for 0.5 h on Cu catalyst at a constant current density of 100 mA cm^{-2} in 1M $KHCO_3$ for 3 h. <3% FE of formate was detected from the anolyte side.

Time (h)	Feed	Faradaic efficiency (%)								
		H_2	CH_4	CO	C_2H_4	EtOH	Acetate	PrOH	Formate	Total
0.17	83.3% CO_2 and 16.7% Ar	13.7	2.5	34.5	16.2	9.0	0.7	5.0	9.1	90.5
0.39		14.4	2.3	34.1	15.2	7.0	0.7	4.1	10.0	87.6
0.67	83.3% CO_2, 15.87% Ar, and 0.83% NO in Ar	14.6	1.0	19.8	7.7	3.0	0.3	1.4	6.7	54.5
0.89		15.5	1.1	18.9	7.0	2.3	0.2	1.5	7.1	53.7
1.2	83.3% CO_2 and 16.7% Ar	19.9	2.3	30.6	13.7	4.9	0.5	3.9	11.1	86.9
1.4		20.3	2.1	28.3	13.7	5.8	0.5	4.0	12.2	86.9
1.8		21.6	2.8	27.8	12.6	5.0	0.7	4.1	13.2	87.7
2.3		22.5	2.4	27.8	12.9	5.3	0.6	3.4	13.4	88.3
2.8		24.1	2.5	27.9	12.1	4.4	0.7	3.5	13.0	88.4

Supplementary Table 3. CO_2 electroreduction performance with the introduction of 0.83% NO impurity at 0.5 h for 0.5 h on Ag catalyst at a constant current density of 100 mA cm^{-2} in 1M $KHCO_3$ for 3 h. <2% FE of formate was detected from the anolyte side.

Time (h)	Feed	Faradaic efficiency (%)			
		H_2	CO	Formate	Total
0.17	83.3% CO_2 and 16.7% Ar	8.0	83.1	3.7	94.8
0.39		10.0	79.2	3.7	92.9
0.67	83.3% CO_2, 15.87% Ar, and 0.83% NO in Ar	6.3	53.1	2.9	62.3
0.89		7.5	54.4	3.6	65.5
1.2	83.3% CO_2 and 16.7% Ar	11.1	76.6	5.2	92.9
1.4		11.6	77.5	6.3	95.4
1.8		12.0	73.6	7.4	93.0
2.3		13.4	71.1	7.9	92.4
2.8		14.5	69.8	8.6	92.9

Supplementary Table 4. CO_2 electroreduction performance with the introduction of 0.83% NO impurity at 0.5 h for 0.5 h on Sn catalyst at a constant current density of 100 mA cm^{-2} in 1M $KHCO_3$ for 3 h. 5-10% FE of formate was detected from the anolyte side and is included in the formate FE.

Time (h)	Feed	Faradaic efficiency (%)			
		H_2	CO	Formate	Total
0.17	83.3% CO_2 and 16.7% Ar	4.6	3.8	88.1	96.6
0.39		4.5	4.3	81.4	90.3
0.67	83.3% CO_2, 15.87% Ar, and 0.83% NO in Ar	4.2	3.9	55.4	63.5
0.89		4.6	4.0	54.0	62.7
1.2	83.3% CO_2 and 16.7% Ar	5.0	5.1	80.0	90.1
1.4		5.6	5.2	78.1	88.9
1.8		7.4	5.2	76.3	88.9
2.3		8.1	5.1	77.8	91.0
2.8		9.0	5.5	76.9	91.4

Supplementary Table 5. Conversion of NO during CO_2RR with the introduction of 0.83% NO, assuming NO is fully converted to NH_3. $Conversion\ (\%) = \frac{Current\ loss\ due\ to\ NO}{Current\ required\ to\ fully\ convert\ NO\ to\ NH_3} \times 100$. Full conversion of NO to NH_3 requires 57.4 mA.

Catalyst	Cu	Ag	Sn
Conversion (%)	59.1	51.6	48.7

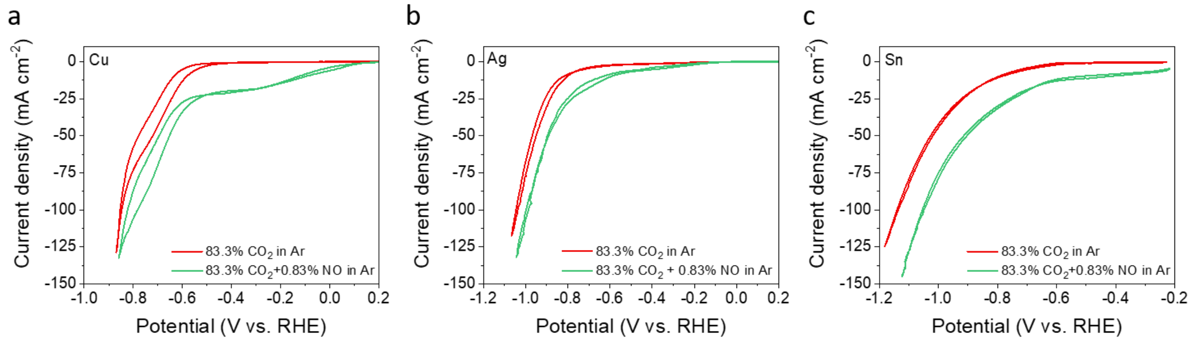

Supplementary Figure 3. Cyclic voltammograms on (a) Cu, (b) Ag, and (c) Sn catalysts in 1M $KHCO_3$ under 83.3% CO_2 and 16.7% Ar, and 83.3% CO_2, 15.87% Ar, and 0.83% NO. Scan rate: 50 mV s^{-1}. Onset potentials and cathodic currents shifted to more positive potentials when 0.83% NO was introduced, suggesting that NORR is more favorable than CO_2RR on all three catalysts.

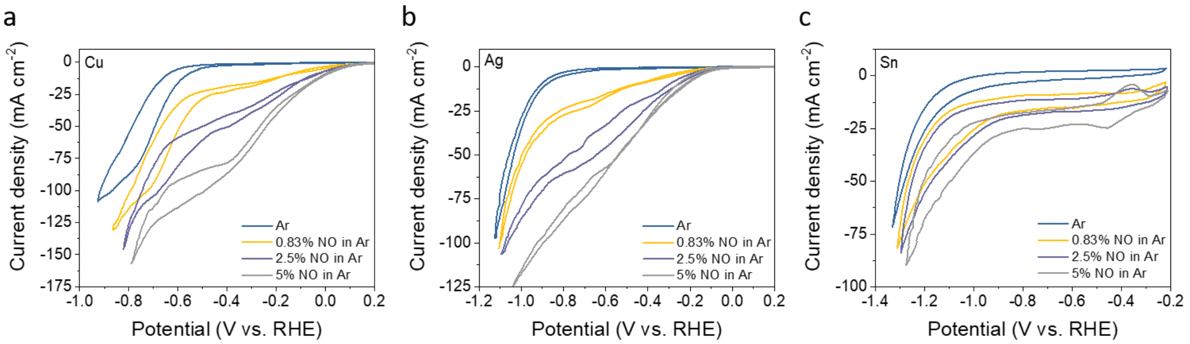

Supplementary Figure 4. Cyclic voltammograms on (a) Cu, (b) Ag, and (c) Sn catalysts in 1M $KHCO_3$ under different concentrations of NO in Ar. Scan rate: 50 mV s^{-1}. Onset potentials of NORR are more positive than CO_2RR, and shifts in CV measurements suggest that NORR is mass transport limited.

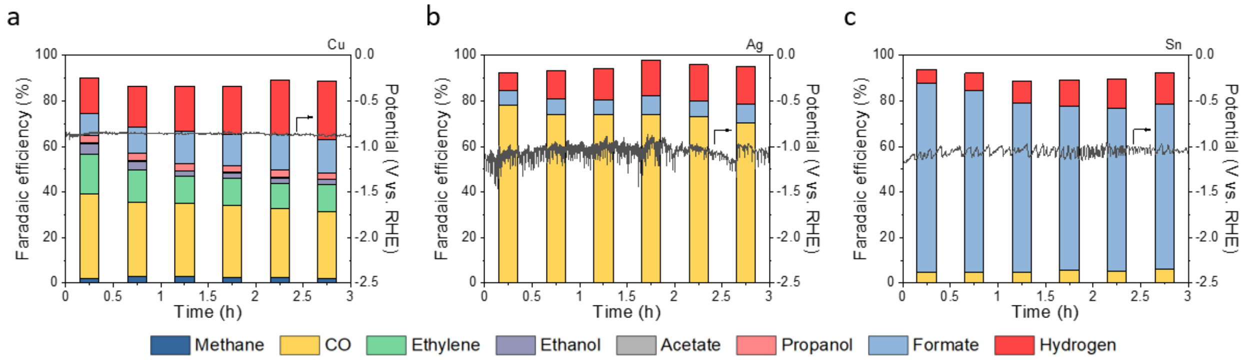

Supplementary Figure 5. Faradaic efficiency and applied potential vs. time with 83.3% CO_2 and 16.7% Ar on (a) Cu, (b) Ag, and (c) Sn catalysts at a constant current density of 100 mA cm^{-2} in 1 M $KHCO_3$ for 3 h. H_2 Faradaic efficiency increases over time due to slow flooding of the electrode, suggesting that NO_x is not responsible for the H_2 FE increase.

Supplementary Table 6. Loss in Faradaic efficiency during CO_2 electroreduction with the introduction of different concentrations of NO on Cu, Ag, and Sn catalysts.

Concentrations of NO (%)	Cu (%)	Ag (%)	Sn (%)
0.0083	0.4	0.6	1.4
0.083	3.2	3.2	3.8
0.83	33.9	29.6	27.9

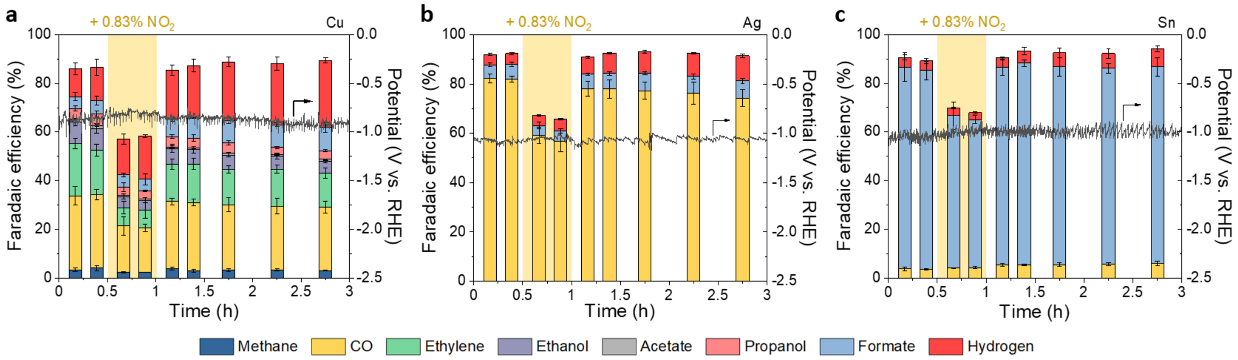

Supplementary Figure 6. Faradaic efficiency and applied potential vs. time on (a) Cu, (b) Ag, and (c) Sn catalysts at a constant current density of 100 mA cm^{-2} in 1 M $KHCO_3$ for 3 h. Gas feeds were 83.3% CO_2 and 16.7% Ar, and 83.3% CO_2, 15.87% Ar, and 0.83% NO_2 (yellow). 0.83% NO_2 was introduced at 0.5 h for 0.5 h. Corresponding Faradaic efficiencies are provided in Tables S7-9. Error bars represent the standard deviation of three independent measurements. Faradaic efficiency decreases with the introduction of NO_2 on all three catalysts.

Supplementary Table 7. CO_2 electroreduction performance with the introduction of 0.83% NO_2 impurity at 0.5 h for 0.5 h on Cu catalyst at a constant current density of 100 mA cm^{-2} in 1M $KHCO_3$ for 3 h. <3% FE of formate was detected from the anolyte side.

Time (h)	Feed	Faradaic efficiency (%)								
		H_2	CH_4	CO	C_2H_4	EtOH	Acetate	PrOH	Formate	Total
0.17	83.3% CO_2 and 16.7% Ar	11.4	3.5	30.4	21.4	8.9	1.1	4.4	4.9	86.0
0.39		13.7	4.1	30.3	18.2	8.9	1.2	4.6	5.6	86.6
0.67	83.3% CO_2, 15.87% Ar, and 0.83% NO_2 in Ar	14.8	2.3	19.2	7.5	4.5	0.5	3.4	5.0	57.2
0.89		17.8	2.5	18.1	7.3	4.2	0.6	3.3	4.7	58.4
1.2	83.3% CO_2 and 16.7% Ar	20.2	3.9	27.6	15.2	6.5	0.7	4.2	7.2	85.4
1.4		21.6	3.1	27.9	15.9	5.9	0.8	3.9	8.2	87.3
1.8		24.0	3.3	26.8	14.4	6.0	0.9	4.3	9.1	88.8
2.3		25.1	3.4	26.1	15.3	5.3	0.7	3.0	9.3	88.1
2.8		27.4	3.1	26.0	14.1	4.7	0.8	3.6	9.7	89.4

Supplementary Table 8. CO_2 electroreduction performance with the introduction of 0.83% NO_2 impurity at 0.5 h for 0.5 h on Ag catalyst at a constant current density of 100 mA cm^{-2} in 1M $KHCO_3$ for 3 h. <2% FE of formate was detected from the anolyte side.

Time (h)	Feed	Faradaic efficiency (%)			
		H_2	CO	Formate	Total
0.17	83.3% CO_2 and 16.7% Ar	4.2	82.3	5.4	92.0
0.39		4.4	81.9	6.0	92.3
0.67	83.3% CO_2, 15.87% Ar, and 0.83% NO_2 in Ar	4.2	59.0	4.0	67.3
0.89		4.8	56.8	4.3	65.8
1.2	83.3% CO_2 and 16.7% Ar	6.9	78.1	5.9	90.9
1.4		8.3	77.9	6.3	92.5
1.8		8.9	77.3	7.0	93.2
2.3		9.4	76.2	6.8	92.4
2.8		10.2	74.2	7.0	91.4

Supplementary Table 9. CO_2 electroreduction performance with the introduction of 0.83% NO_2 impurity at 0.5 h for 0.5 h on Sn catalyst at a constant current density of 100 mA cm^{-2} in 1M $KHCO_3$ for 3 h. 5-10% FE of formate was detected from the anolyte side and is included in the formate FE.

Time (h)	Feed	Faradaic efficiency (%)			
		H_2	CO	Formate	Total
0.17	83.3% CO_2 and 16.7% Ar	4.0	3.9	82.7	90.6
0.39		4.0	3.8	81.4	89.2
0.67	83.3% CO_2, 15.87% Ar, and 0.83% NO_2 in Ar	3.1	4.2	62.5	69.8
0.89		2.8	4.4	60.8	67.9
1.2	83.3% CO_2 and 16.7% Ar	4.0	5.5	80.9	90.4
1.4		5.0	5.5	82.8	93.3
1.8		5.8	5.7	81.1	92.5
2.3		6.2	5.8	80.3	92.2
2.8		7.4	6.2	80.6	94.2

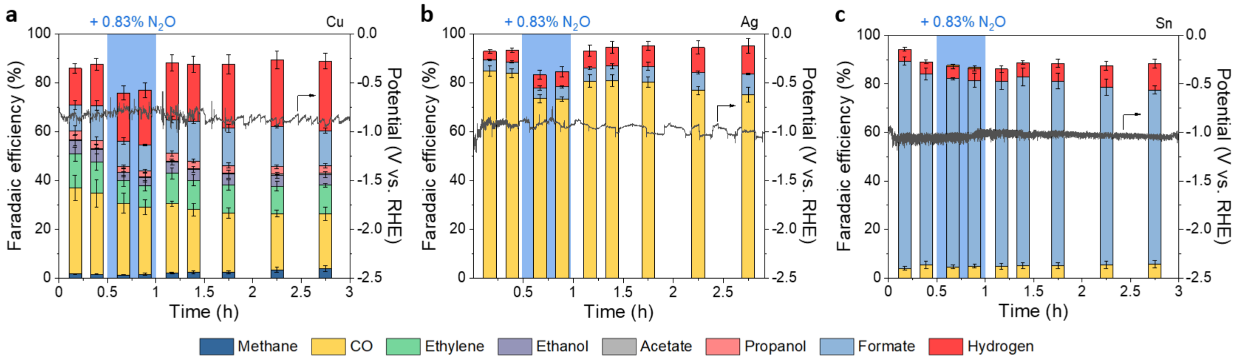

Supplementary Figure 7. Faradaic efficiency and applied potential vs. time on (a) Cu, (b) Ag, and (c) Sn catalysts at a constant current density of 100 mA cm^{-2} in 1 M $KHCO_3$ for 3 h. Gas feeds were 83.3% CO_2 and 16.7% Ar, and 83.3% CO_2, 15.87% Ar, and 0.83% N_2O (blue). 0.83% N_2O was introduced at 0.5 h for 0.5 h. Corresponding Faradaic efficiencies are provided in Tables S10-12. Error bars represent the standard deviation of three independent measurements. Faradaic efficiency decreases with the introduction of N_2O on Cu and Ag catalysts, while N_2O has negligible effect on Sn catalyst.

Supplementary Table 10. CO_2 electroreduction performance with the introduction of 0.83% N_2O impurity at 0.5 h for 0.5 h on Cu catalyst at a constant current density of 100 mA cm^{-2} in 1M $KHCO_3$ for 3 h. <3% FE of formate was detected from the anolyte side.

Time (h)	Feed	Faradaic efficiency (%)								
		H_2	CH_4	CO	C_2H_4	EtOH	Acetate	PrOH	Formate	Total
0.17	83.3% CO_2 and 16.7% Ar	15.0	1.8	35.2	13.9	5.6	0.3	3.7	10.5	85.9
0.39		16.9	1.7	33.0	12.8	5.2	0.3	3.3	14.2	87.5
0.67	83.3% CO_2, 15.87% Ar, and 0.83% N_2O in Ar	19.6	1.4	29.4	9.2	3.3	0.2	2.3	10.4	75.7
0.89		22.4	1.6	27.6	8.6	3.6	0.2	2.2	10.7	77.0
1.2	83.3% CO_2 and 16.7% Ar	23.2	2.1	28.4	12.4	4.6	0.4	3.1	13.8	88.1
1.4		23.3	2.5	25.6	12.0	4.5	0.3	3.1	16.2	87.5
1.8		26.1	2.6	24.1	11.5	4.4	0.5	3.0	15.2	87.5
2.3		27.4	3.5	23.0	11.2	4.4	0.5	3.0	16.4	89.4
2.8		28.4	4.1	22.3	11.7	4.3	0.6	3.1	14.3	88.8

Supplementary Table 11. CO_2 electroreduction performance with the introduction of 0.83% N_2O impurity at 0.5 h for 0.5 h on Ag catalyst at a constant current density of 100 mA cm^{-2} in 1M $KHCO_3$ for 3 h. <2% FE of formate was detected from the anolyte side.

Time (h)	Feed	Faradaic efficiency (%)			
		H_2	CO	Formate	Total
0.17	83.3% CO_2 and 16.7% Ar	3.5	84.9	4.4	92.8
0.39		4.7	83.9	4.6	93.2
0.67	83.3% CO_2, 15.87% Ar, and 0.83% N_2O in Ar	5.4	73.4	4.4	83.3
0.89		6.1	73.3	5.0	84.5
1.2	83.3% CO_2 and 16.7% Ar	7.1	80.7	5.3	93.1
1.4		7.7	80.8	6.0	94.5
1.8		8.5	80.2	6.5	95.2
2.3		10.1	76.9	7.4	94.4
2.8		11.5	75.1	8.5	95.1

Supplementary Table 12. CO_2 electroreduction performance with the introduction of 0.83% N_2O impurity at 0.5 h for 0.5 h on Sn catalyst at a constant current density of 100 mA cm^{-2} in 1M $KHCO_3$ for 3 h. 5-10% FE of formate was detected from the anolyte side and is included in the formate FE.

Time (h)	Feed	Faradaic efficiency (%)			
		H_2	CO	Formate	Total
0.17	83.3% CO_2 and 16.7% Ar	4.9	3.9	85.4	94.3
0.39		4.9	5.4	78.7	89.0
0.67	83.3% CO_2, 15.87% Ar, and 0.83% N_2O in Ar	4.9	4.6	77.6	87.1
0.89		4.8	4.9	76.6	86.2
1.2	83.3% CO_2 and 16.7% Ar	5.1	4.8	76.2	86.1
1.4		5.9	5.2	77.7	88.7
1.8		7.3	5.2	76.0	88.5
2.3		9.0	5.4	73.1	87.5
2.8		10.9	5.7	71.8	88.4

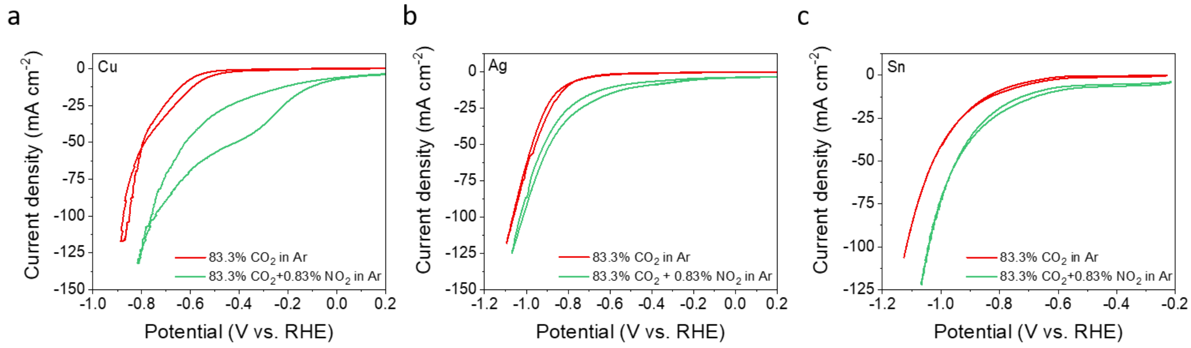

Supplementary Figure 8. Cyclic voltammograms on (a) Cu, (b) Ag, and (c) Sn catalysts in 1M $KHCO_3$ under 83.3% CO_2 and 16.7% Ar, and 83.3% CO_2, 15.87% Ar, and 0.83% NO_2. Scan rate: 50 mV s^{-1}. Onset potentials and cathodic currents shift to more positive potentials when 0.83% NO_2 is introduced, suggesting that NO_2RR is more favorable than CO_2RR on all three catalysts.

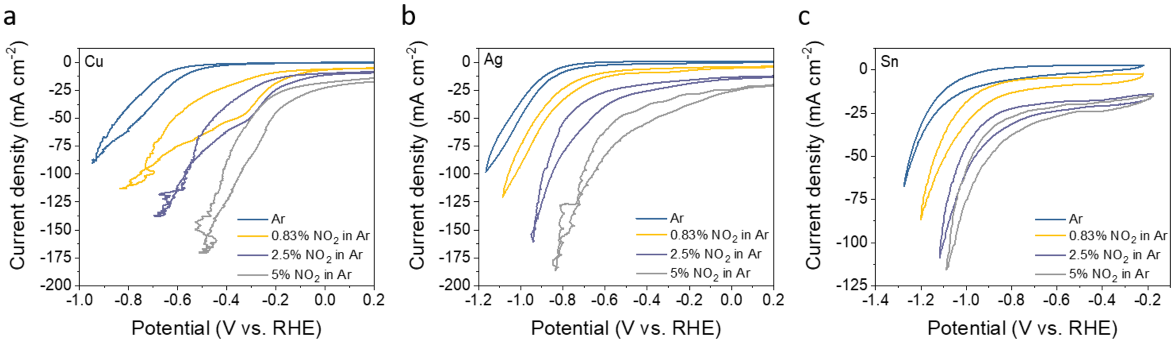

Supplementary Figure 9. Cyclic voltammograms on (a) Cu, (b) Ag, and (c) Sn catalysts in 1M $KHCO_3$ under different concentrations of NO_2 in Ar. Scan rate: 50 mV s^{-1}. Onset potentials of NO_2RR are more positive than CO_2RR, and shifts in CV measurements suggest that NO_2RR is mass transport limited.

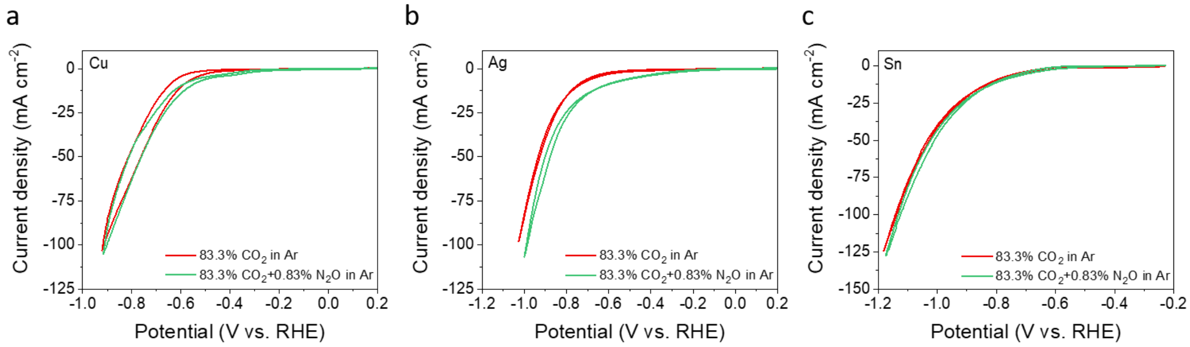

Supplementary Figure 10. Cyclic voltammograms on (a) Cu, (b) Ag, and (c) Sn catalysts in 1M $KHCO_3$ under 83.3% CO_2 and 16.7% Ar, and 83.3% CO_2, 15.87% Ar, and 0.83% N_2O. Scan rate: 50 mV s^{-1}. Onset potentials and cathodic currents shift to more positive potentials when N_2O is introduced on Cu and Ag catalysts, suggesting that N_2ORR is more favorable than CO_2RR on Cu and Ag catalysts. N_2O has negligible effect on Sn catalyst.

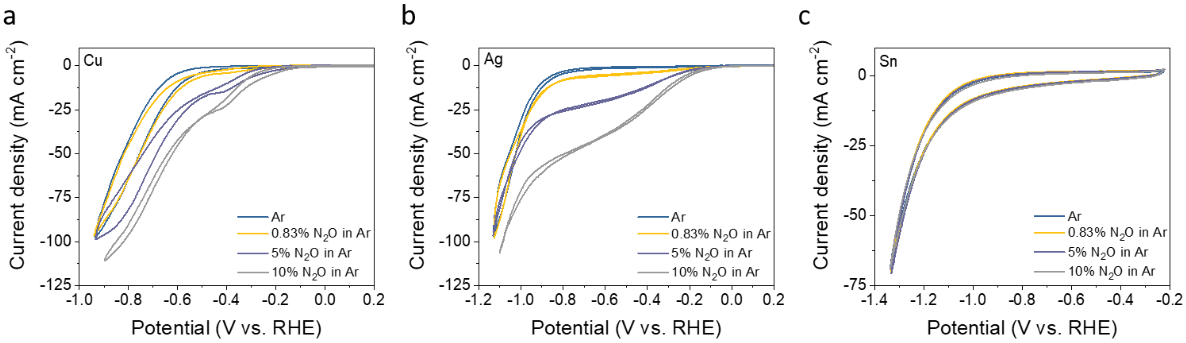

Supplementary Figure 11. Cyclic voltammograms on (a) Cu, (b) Ag, and (c) Sn catalysts in 1M $KHCO_3$ under different concentrations of N_2O in Ar. Scan rate: 50 mV s^{-1}. Onset potentials of N_2ORR are more positive than CO_2RR and shifts in CV measurements suggest that N_2ORR is mass transport limited on Cu and Ag catalysts. N_2O has negligible effect on Sn catalyst.

Supplementary Table 13. Loss in Faradaic efficiency during CO_2 electroreduction with the introduction of 0.83% NO, 0.83% NO_2, and 0.83% N_2O on Cu, Ag, and Sn catalysts.

Impurity	Cu (%)	Ag (%)	Sn (%)
NO	33.9	29.6	27.9
NO_2	30.8	25.6	22.9
N_2O	13.3	10.2	1.4

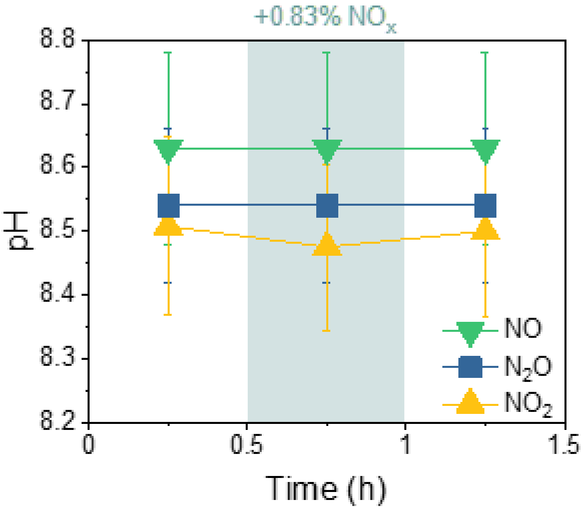

Supplementary Figure 12. pH measured at the outlet of the electrolyzer from a constant current 100-mA cm^{-2} CO_2RR experiment with the introduction of various NO_x. pH of 1M $KHCO_3$ before entering the electrolyzer was 7.8 ± 0.1. Error bars represent the standard deviation of independent measurements from Cu, Ag, and Sn catalysts. The presence of NO and N_2O has negligible effect in pH, while the presence of NO_2 slightly decreases the pH by 0.03.

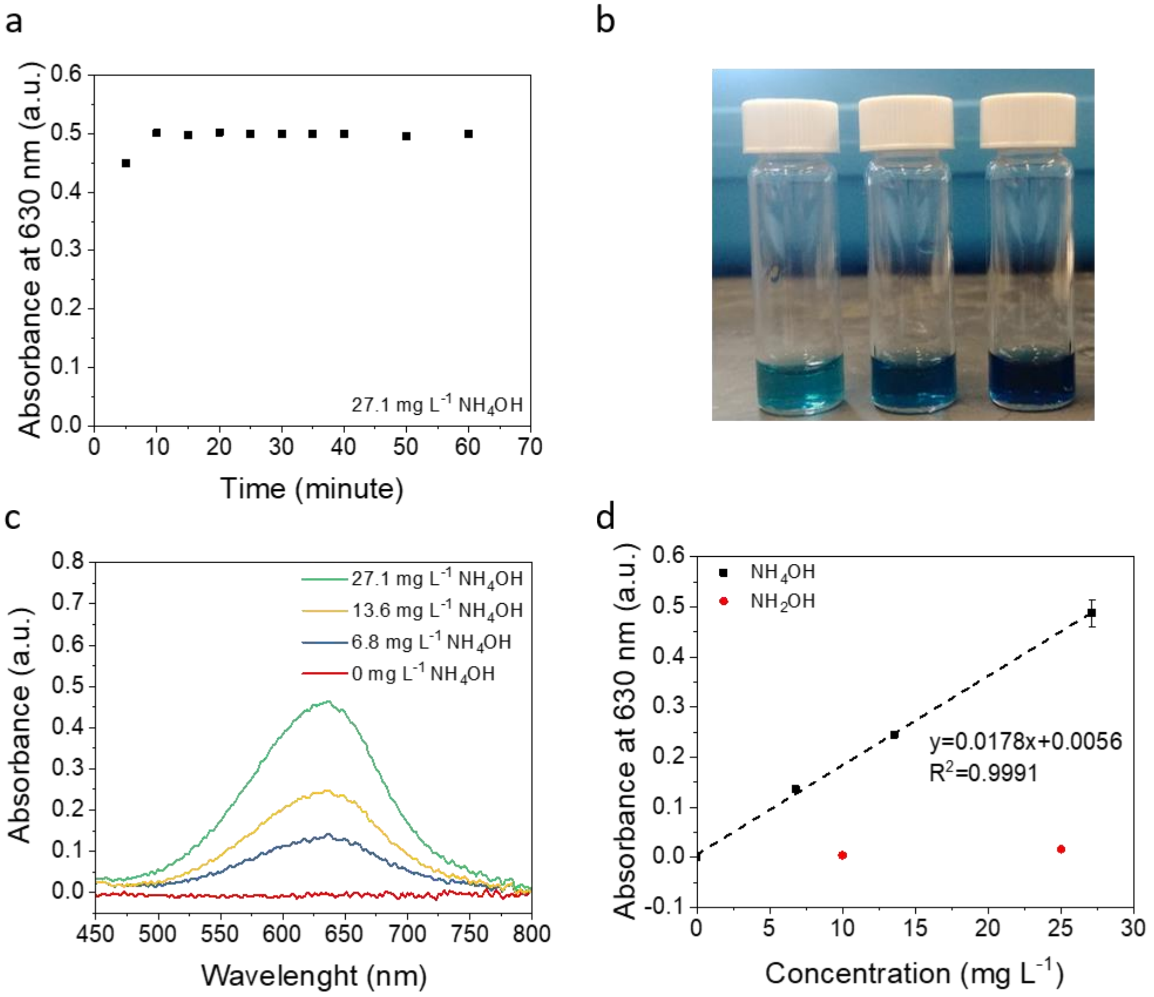

Supplementary Figure 13. Ammonia quantification using indophenol blue method. (a) Absorbance vs. time of 27.1 mg L^{-1} NH_4OH. Reaction is complete after 10 minutes. (b) Photograph of 6.8, 13.6, and 27.1 mg L^{-1} NH_4OH (from left to right) at 20 minutes. (c) Absorption spectra for different concentrations of NH_4OH measured at 20 minutes. (d) Calibration curve for NH_4OH. Absorbance was measured at 20 minutes. NH_2OH has negligible interference. All solutions were prepared in 0.25M $KHCO_3$ to match the condition of the liquid products in the electrolyte.

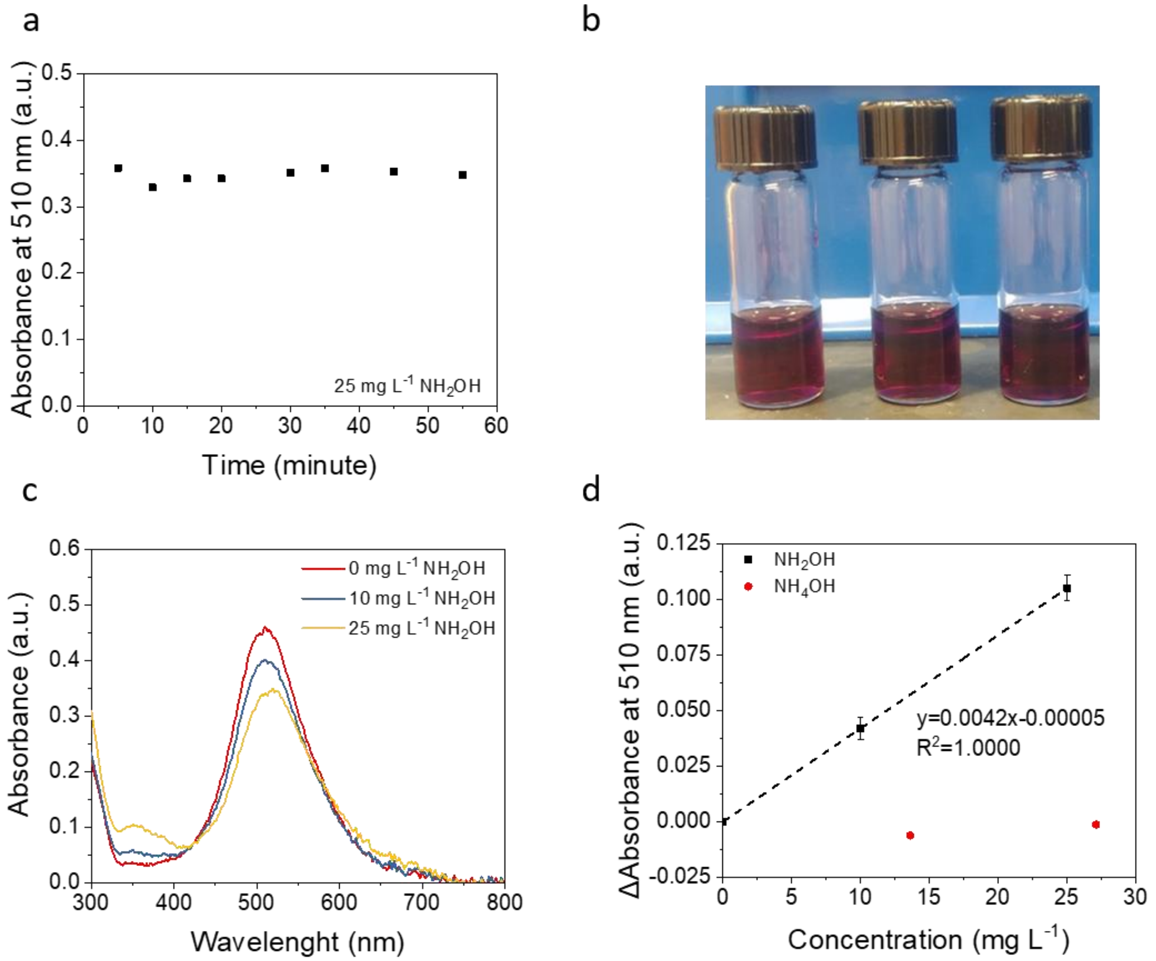

Supplementary Figure 14. Hydroxylamine quantification. (a) Absorbance vs. time of 25 mg L^{-1} NH_2OH. Reaction is complete after 10 minutes. (b) Photograph of 0, 10, and 25 mg L^{-1} NH_2OH (from left to right) at 20 minutes. (c) Absorption spectra for different concentrations of NH_2OH measured at 20 minutes. (d) Calibration curve for NH_2OH. Absorbance was measured at 20 minutes. Absorbance was subtracted from that of 0 mg L^{-1} NH_2OH. NH_4OH has negligible interference. All solutions were prepared in 0.25M $KHCO_3$ to match the condition of the liquid products in the electrolyte.

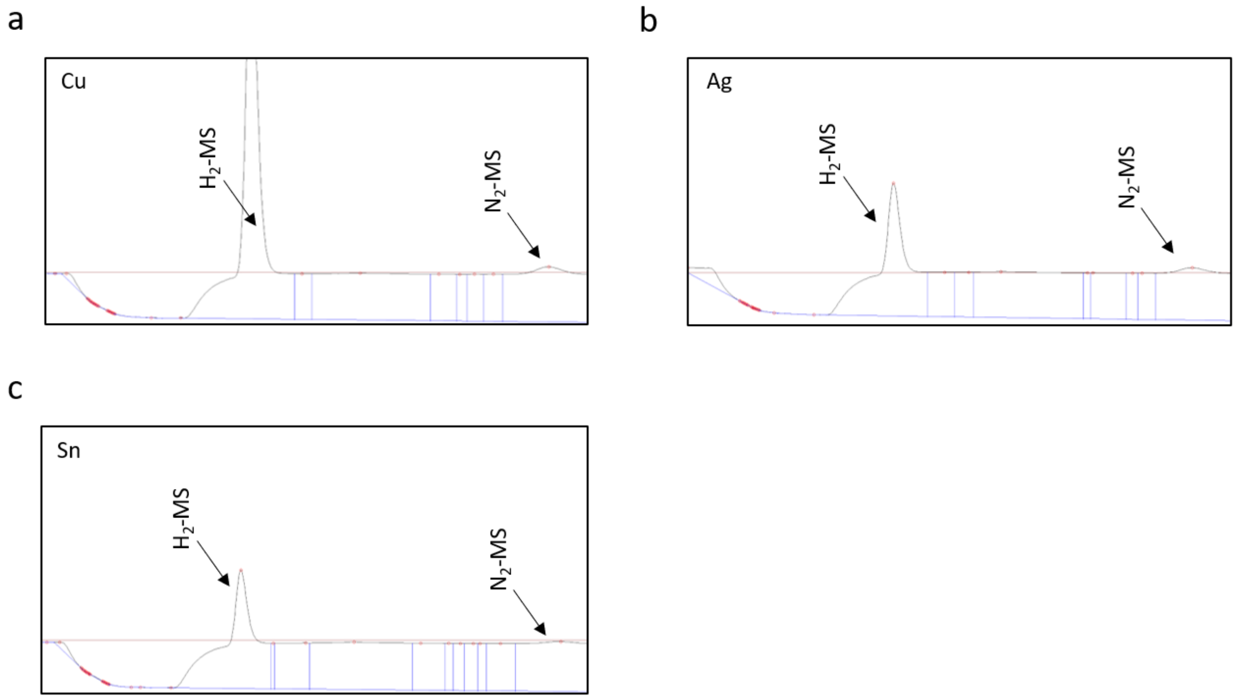

Supplementary Figure 15. Chromatogram of gas products from electrolysis in 83.3% CO_2, 15.87% Ar, and 0.83% NO on (a) Cu, (b) Ag, and (c) Sn catalysts at 100 mA cm^{-2}. TCD and Molecular sieve 5 A (MS) column are used, and 0 to 2.1 min is shown.

Supplementary Table 14. Faradaic efficiency of NO electroreduction products produced during electrolysis with 83.3% CO_2, 15.87% Ar, and 0.83% NO on Cu, Ag, and Sn catalysts at a constant current density of 100 mA cm^{-2} in 1 M $KHCO_3$ for 3 h. Concentration of N_2O in the gas product stream was below the detection limit of GC, suggesting that N_2O FE is below 2% on all three catalysts.

Impurity	Cu (%)	Ag (%)	Sn (%)
NH_3	26.5	7.8	1.9
NH_2OH	-	11.6	13.7
N_2	4.4	2.7	0.9

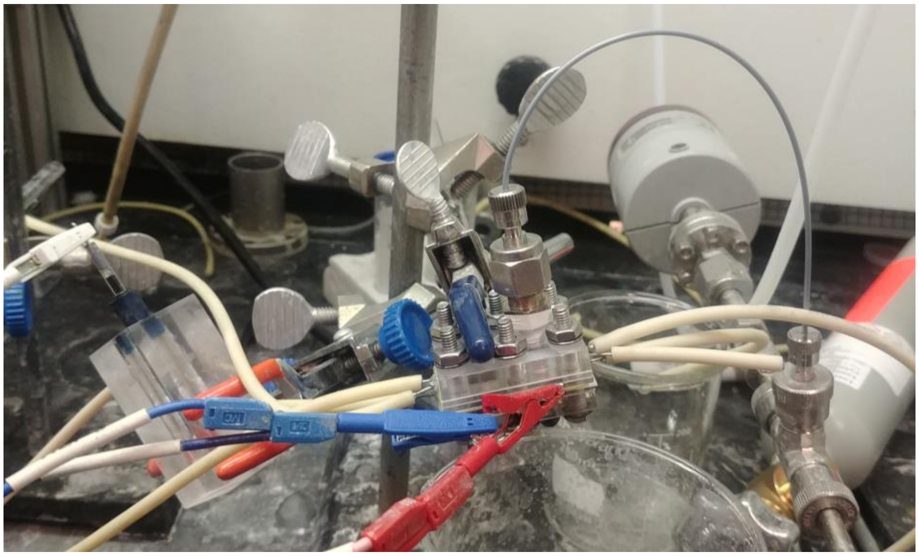

Supplementary Figure 16. Photograph of the FEMS setup.

Supplementary Note: Design of FEMS measurement

To differentiate N_2 (m/z=28, 14) from N_2O (m/z=44, 30, 28, 14), the signals associated with N_2O need to be determined from m/z=44 or 30 signals. However, when the gas feed is 83.3% CO_2+0.83% NO in Ar, m/z=44 and 30 signals are dominated by CO_2 and NO, respectively, making the determination of the relatively small N_2O signals unreliable. In addition, CO_2 reduction products such as CO, methane, ethylene, and ethanol complicate the analysis of NORR products. As an alternative, FEMS experiment was conducted using 0.83% NO in Ar in the absence of CO_2 to gain insight on the NORR products.

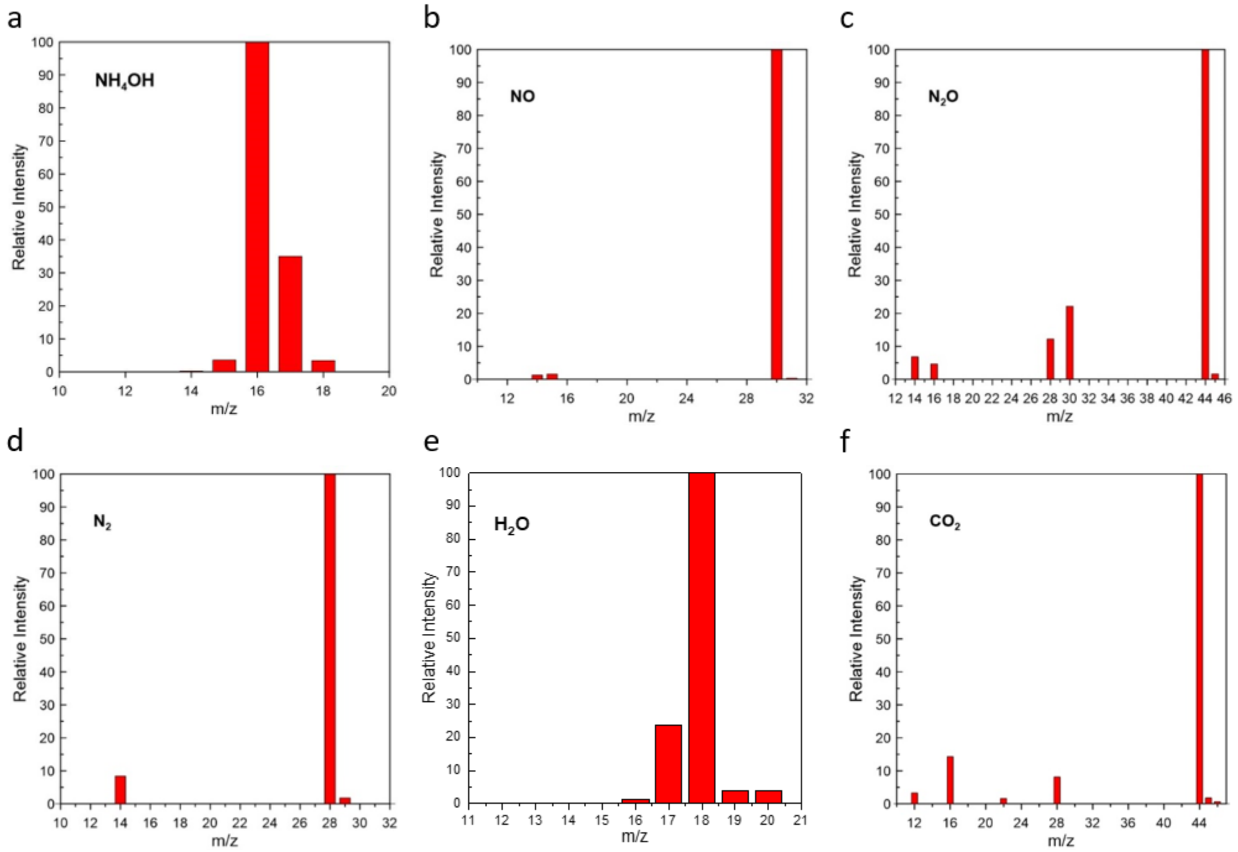

Supplementary Figure 17. Mass spectra of (a) NH_4OH, (b) NO, (c) N_2O, (d) N_2, (e) H_2O, and (f) CO_2. MS signals were deconvoluted using the following mass spectra.

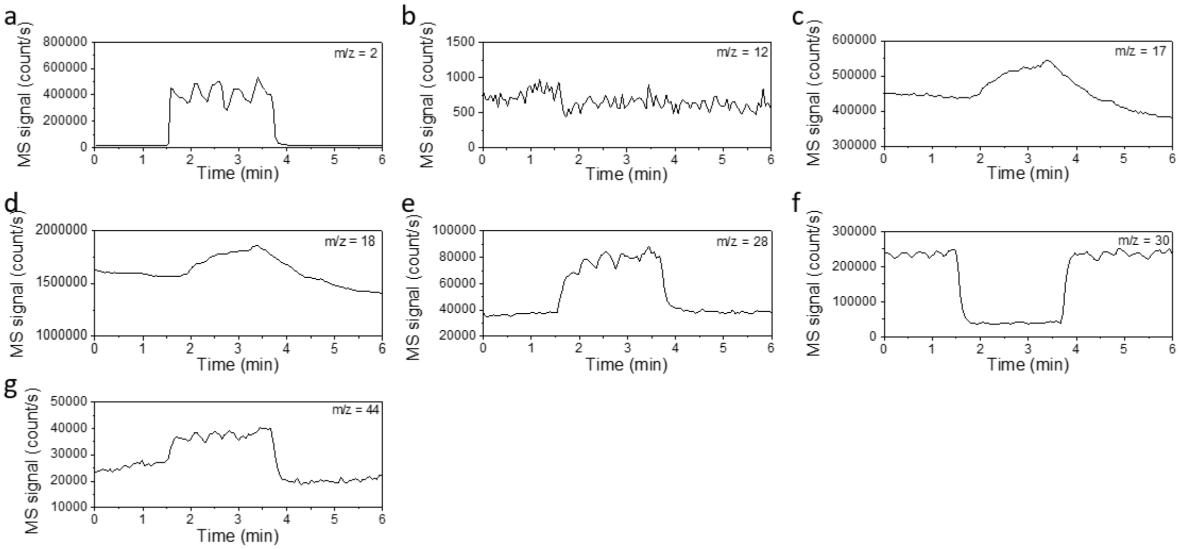

Supplementary Figure 18. MS signal vs. time obtained from FEMS on Cu in 1M $KHCO_3$ with 0.83% NO in Ar before deconvolution for (a) m/z=2, (b) m/z=12, (c) m/z=17, (d) m/z=18, (e) m/z=28, (f) m/z=30, and (g) m/z=44. -0.90 V vs. RHE was applied for approximately 2 minutes starting at t=1.5 min.

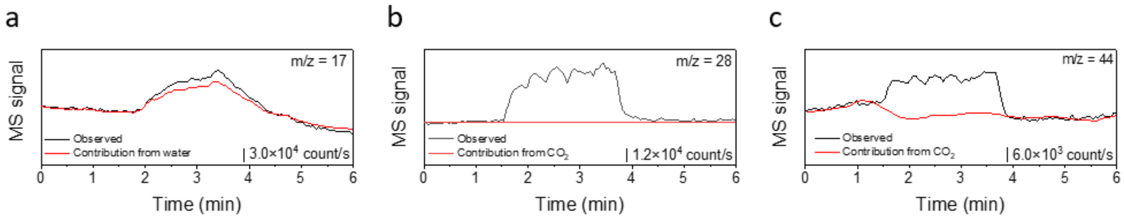

Supplementary Figure 19. MS signal vs. time obtained from FEMS on Cu in 1M $KHCO_3$ with 0.83% NO in Ar for (a) m/z=17 with contribution from water obtained from m/z=18, (b) m/z=28 with contribution from CO_2 in electrolyte obtained from m/z=12, and (c) m/z=44 with contribution from CO_2 in electrolyte obtained from m/z=12. m/z=12 has been smoothed. -0.90 V vs. RHE was applied for approximately 2 minutes starting at t=1.5 min. The differences are attributed to NORR products.

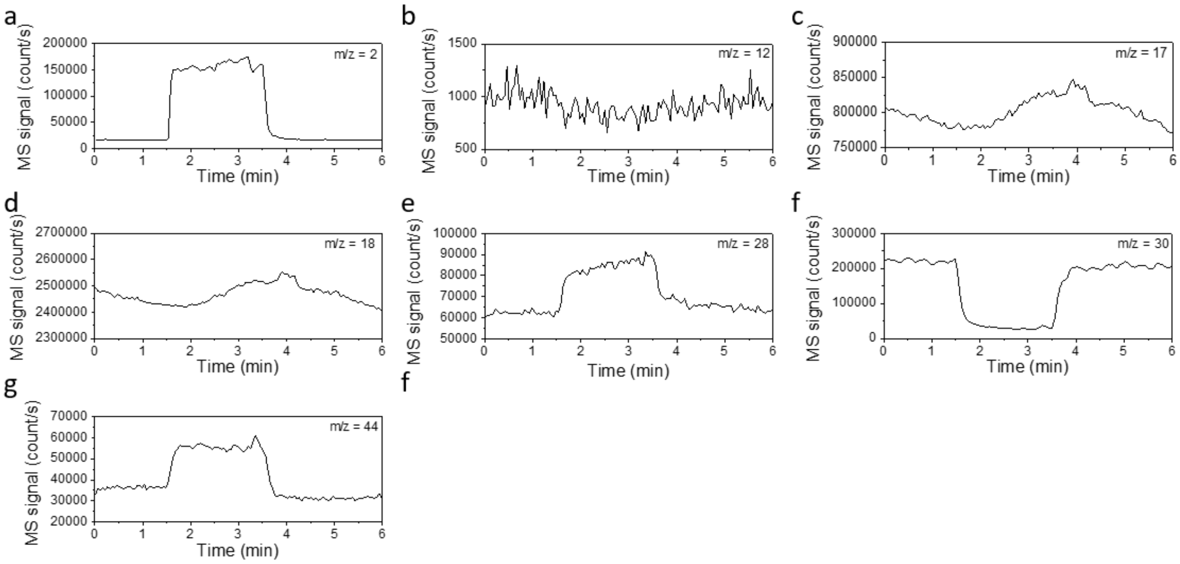

Supplementary Figure 20. MS signal vs. time obtained from FEMS on Ag in 1M $KHCO_3$ with 0.83% NO in Ar before deconvolution for (a) m/z=2, (b) m/z=12, (c) m/z=17, (d) m/z=18, (e) m/z=28, (f) m/z=30, and (g) m/z=44. -1.00 V vs. RHE was applied for approximately 2 minutes starting at t=1.5 min.

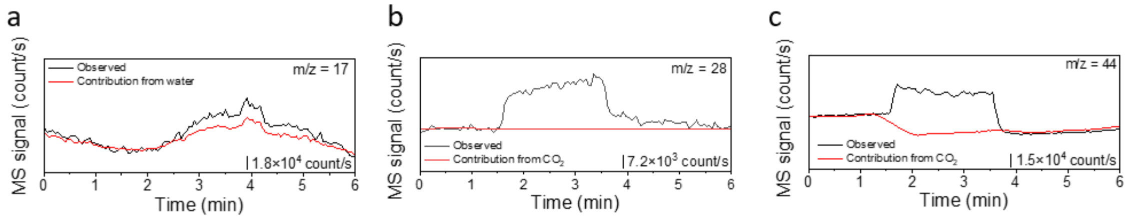

Supplementary Figure 21. MS signal vs. time obtained from FEMS on Ag in 1M $KHCO_3$ with 0.83% NO in Ar for (a) m/z=17 with contribution from water obtained from m/z=18, (b) m/z=28 with contribution from CO_2 in electrolyte obtained from m/z=12, and (c) m/z=44 with contribution from CO_2 in electrolyte obtained from m/z=12. m/z=12 has been smoothed. -1.00 V vs. RHE was applied for approximately 2 minutes starting at t=1.5 min. The differences are attributed to NORR products.

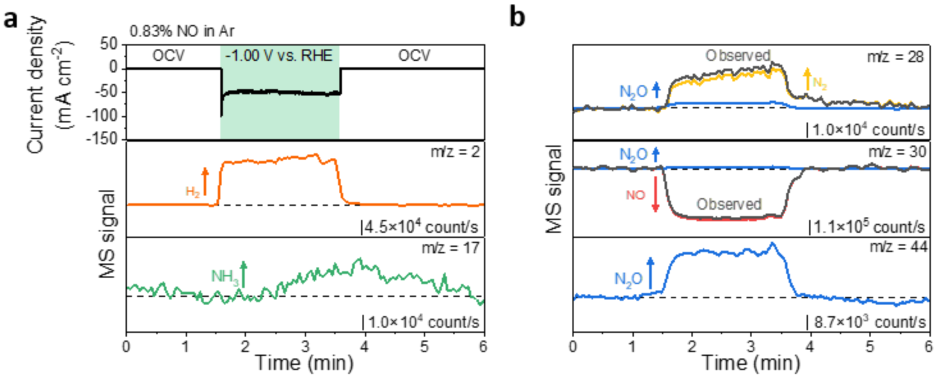

Supplementary Figure 22. (a) Measured current density vs. time, and deconvoluted MS signal vs. time for m/z=2, m/z=17, (b) m/z=28, m/z=30, and m/z=44 from FEMS on Ag catalyst in 1M $KHCO_3$ with 0.83% NO in Ar. -1.00 V vs. RHE was applied for approximately 2 minutes starting at t=1.5 min. NORR products have been deconvoluted using the mass spectra of individual products shown in Supplementary Fig. 17. Additional information is provided in the methods section and Supplementary Figs. 20 and 21.

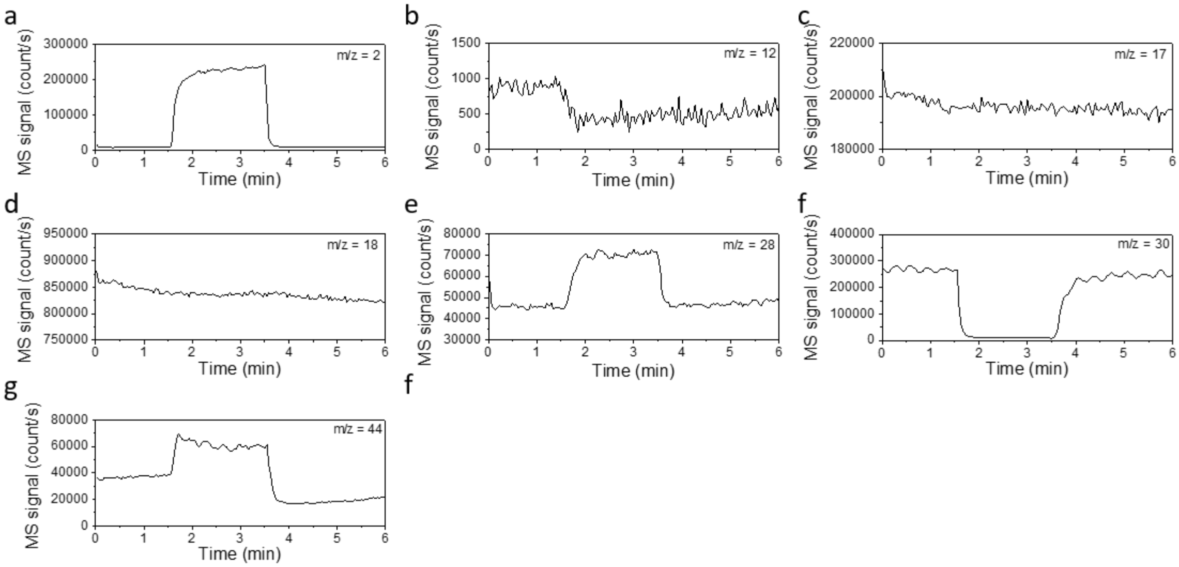

Supplementary Figure 23. MS signal vs. time obtained from FEMS on Sn in 1M $KHCO_3$ with 0.83% NO in Ar before deconvolution for (a) m/z=2, (b) m/z=12, (c) m/z=17, (d) m/z=18, (e) m/z=28, (f) m/z=30, and (g) m/z=44. -1.05 V vs. RHE was applied for approximately 2 minutes starting at t=1.5 min.

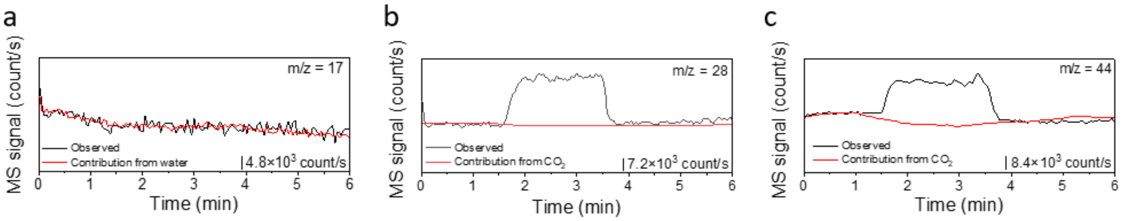

Supplementary Figure 24. MS signal vs. time obtained from FEMS on Sn in 1M $KHCO_3$ with 0.83% NO in Ar for (a) m/z=17 with contribution from water obtained from m/z=18, (b) m/z=28 with contribution from CO_2 in electrolyte obtained from m/z=12, and (c) m/z=44 with contribution from CO_2 in electrolyte obtained from m/z=12. m/z=12 has been smoothed. -1.05 V vs. RHE was applied for approximately 2 minutes starting at t=1.5 min. The differences are attributed to NORR products.

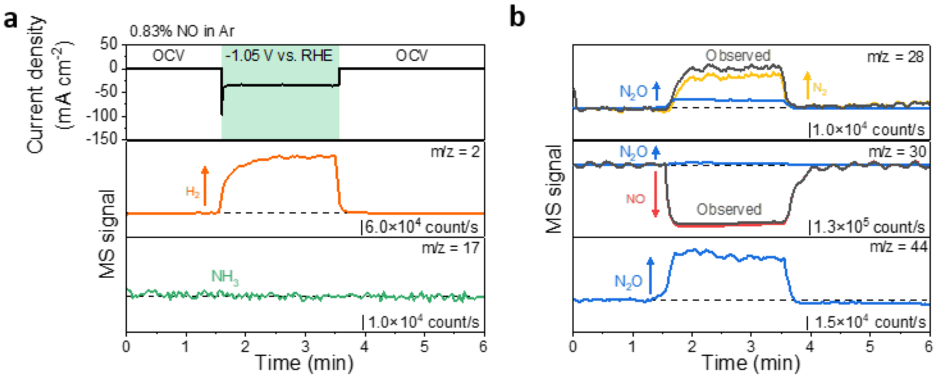

Supplementary Figure 25. (a) Measured current density vs. time, and deconvoluted MS signal vs. time for m/z=2, m/z=17, (b) m/z=28, m/z=30, and m/z=44 from FEMS on Sn catalyst in 1M $KHCO_3$ with 0.83% NO in Ar. -1.05 V vs. RHE was applied for approximately 2 minutes starting at t=1.5 min. NORR products have been deconvoluted using the mass spectra of individual products shown in Supplementary Fig. 17. Additional information is provided in the methods section and Supplementary Figs. 23 and 24.

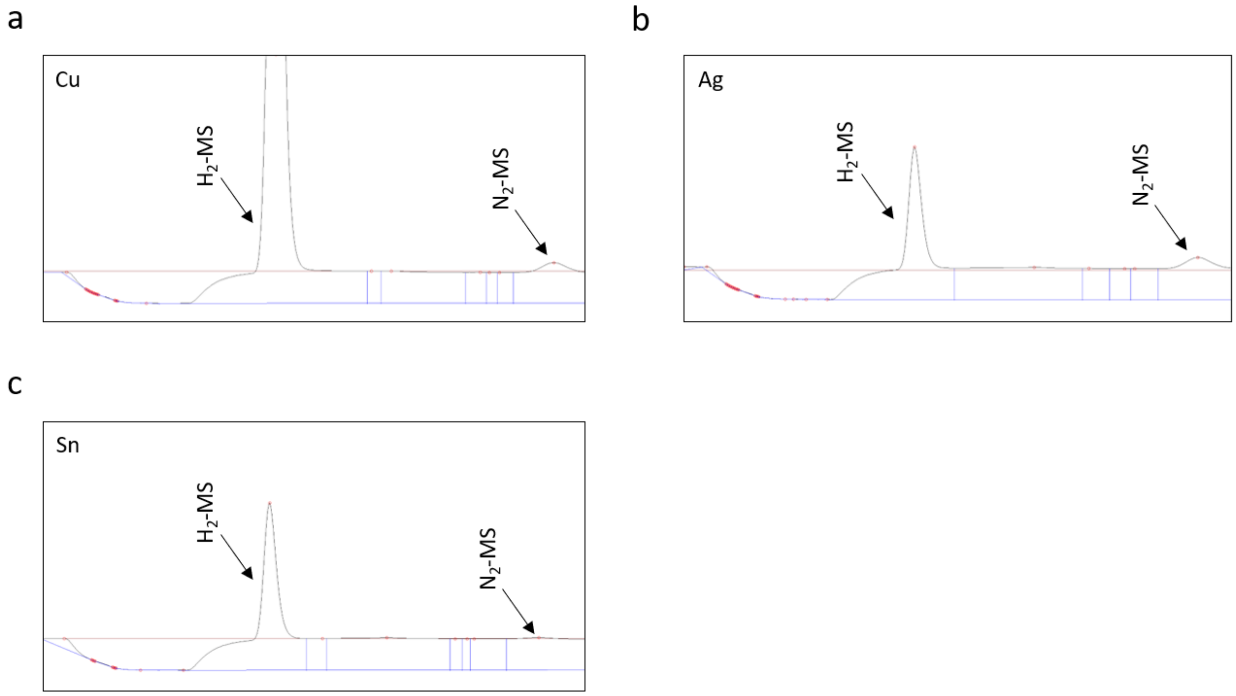

Supplementary Figure 26. Chromatogram of gas products from electrolysis in 83.3% CO_2+0.83% N_2O in Ar on (a) Cu, (b) Ag, and (c) Sn catalysts at 100 mA cm^{-2}. TCD and Molecular sieve 5 A (MS) column were used, and 0 to 2 min is shown.

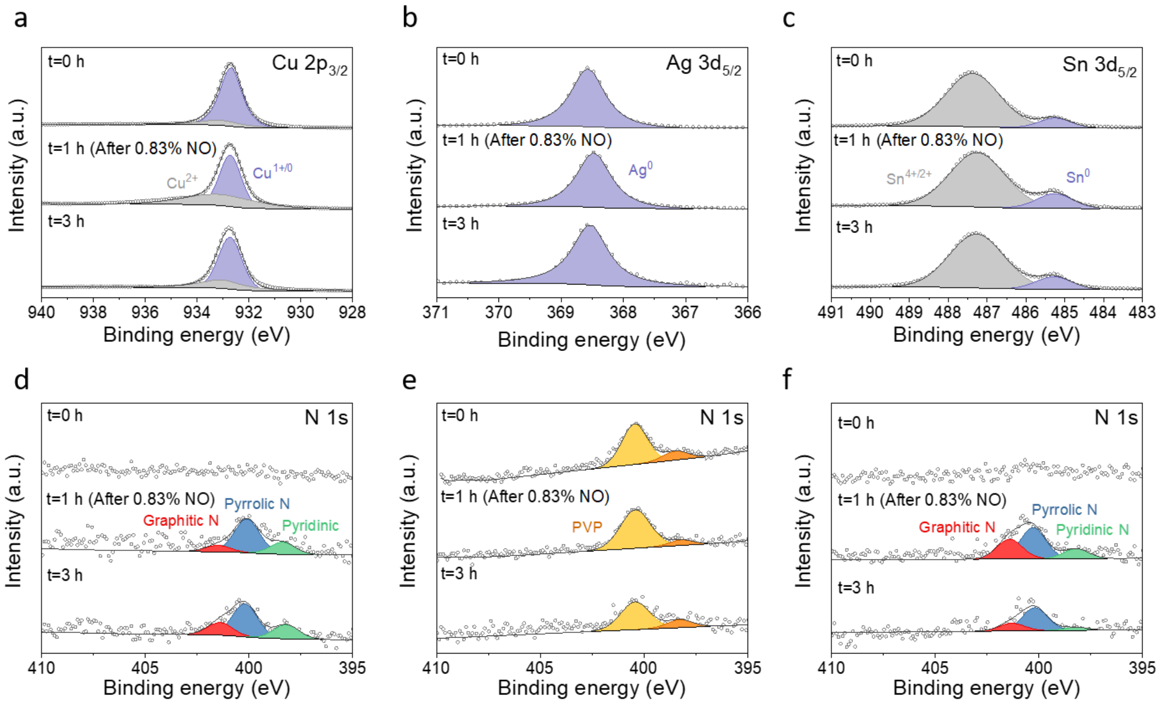

Supplementary Figure 27. XPS measurements of (a) Cu, (b) Ag, and (c) Sn electrodes before exposure to 0.83% NO (t=0 h), after exposure to 0.83% NO (t=1 h), and after 3 h electrolysis (t=3 h) from a 100 mA cm^{-2} constant current CO_2RR experiment with the introduction of 0.83% NO. Corresponding N 1s XPS measurements of (d) Cu, (e) Ag, and (f) Sn electrodes. Incorporation of N into GDL is observed on Cu and Sn electrodes. In the case of Ag electrode, the investigation of N incorporation is limited due to the presence of PVP surfactant. Corresponding details are provided in Supplementary Table 15.

Supplementary Table 15. XPS N 1s peak positions and surface nitrogen content (wt %) of Cu, Ag, and Sn samples obtained from a 100 mA cm^{-2} constant current CO_2RR experiment with the introduction of 0.83% NO. Sample time refers to the time at which the electrode was taken out of the electrolyzer. N content has been calculated using the following equation: N content (%) = $\frac{\Sigma N\ (wt\ \%)}{\Sigma N\ (wt\ \%)+metal\ (wt\ \%)} \times 100$, where ΣN (wt %) = graphitic N (wt %) + pyrollic N (wt %) + pyridinic N (wt%) or PVP (wt%), and metal = Cu, Ag, or Sn.

	Sample time (h)	Graphitic (eV)	Pyrrolic (eV)	Pyridinic (eV)	PVP (eV)	Total N content (wt %)
Cu	1	401.5	400.2	398.4	-	1.48
	3	401.4	400.2	398.2	-	1.12
Ag	0	-	-	-	400.5, 398.5	2.72
	1	-	-	-	400.4, 398.5	2.92
	3	-	-	-	400.4, 398.3	2.40
Sn	1	401.4	400.3	398.2	-	1.03
	3	401.3	400.2	398.1	-	0.64

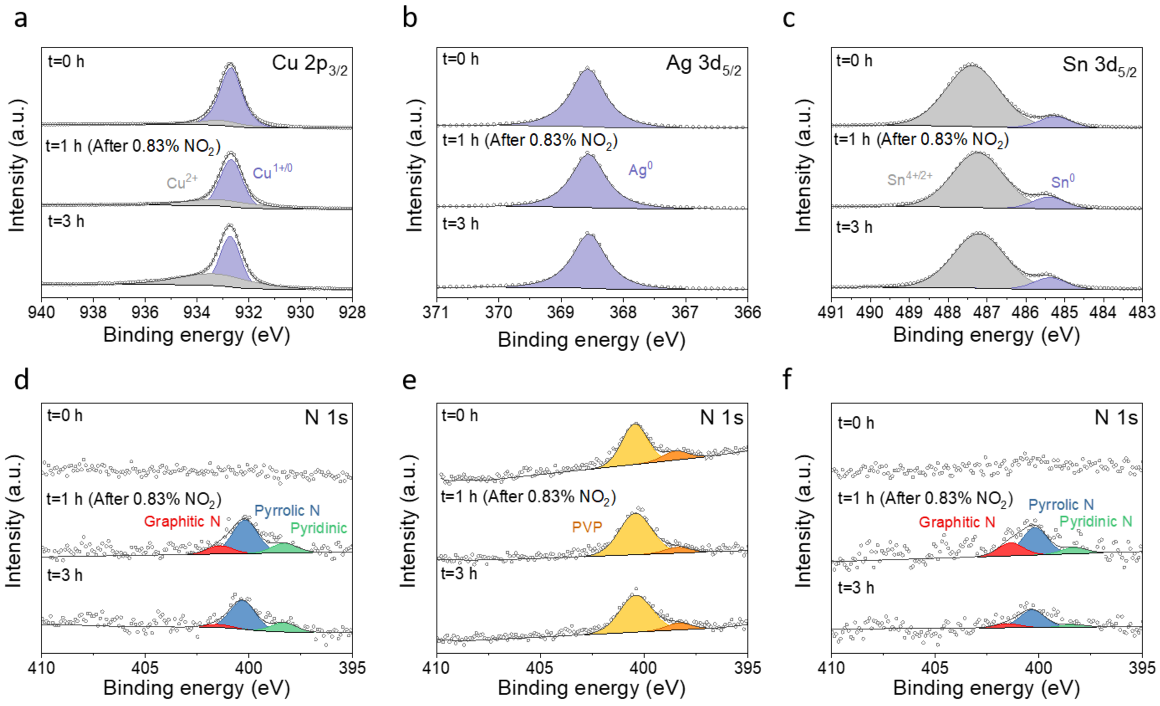

Supplementary Figure 28. XPS measurements of (a) Cu, (b) Ag, and (c) Sn electrodes before exposure to 0.83% NO_2 (t=0 h), after exposure to 0.83% NO_2 (t=1 h), and after 3 h electrolysis (t=3 h) from a 100 mA cm^{-2} constant current CO_2RR experiment with the introduction of 0.83% NO_2. Corresponding N 1s XPS measurements of (d) Cu, (e) Ag, and (f) Sn electrodes. Incorporation of N into GDL is observed on Cu and Sn electrodes. In the case of Ag electrode, the investigation of N incorporation is limited due to the presence of PVP surfactant. Corresponding details are provided in Supplementary Table 16.

Supplementary Table 16. XPS N 1s peak positions and surface nitrogen content (wt %) of Cu, Ag, and Sn samples obtained from a 100 mA cm^{-2} constant current CO_2RR experiment with the introduction of 0.83% NO_2. Sample time refers to the time at which the electrode was taken out of the electrolyzer. N content has been calculated using the following equation: N content = $\frac{\Sigma N\ (wt\ \%)}{\Sigma N\ (wt\ \%)+metal\ (wt\ \%)} \times 100$, where ΣN (wt %) = graphitic N (wt %) + pyrollic N (wt %) + pyridinic N (wt%) or PVP (wt%), and metal = Cu, Ag, or Sn.

	Sample time (h)	Graphitic (eV)	Pyrrolic (eV)	Pyridinic (eV)	PVP (eV)	Total N content (wt %)
Cu	1	401.4	400.2	398.3	-	0.97
	3	401.4	400.3	398.2	-	1.26
Ag	0	-	-	-	400.5, 398.5	2.72
	1	-	-	-	400.4, 398.4	3.63
	3	-	-	-	400.4, 398.3	3.22
Sn	1	401.4	400.2	398.2	-	2.25
	3	401.2	400.3	398.2	-	1.47

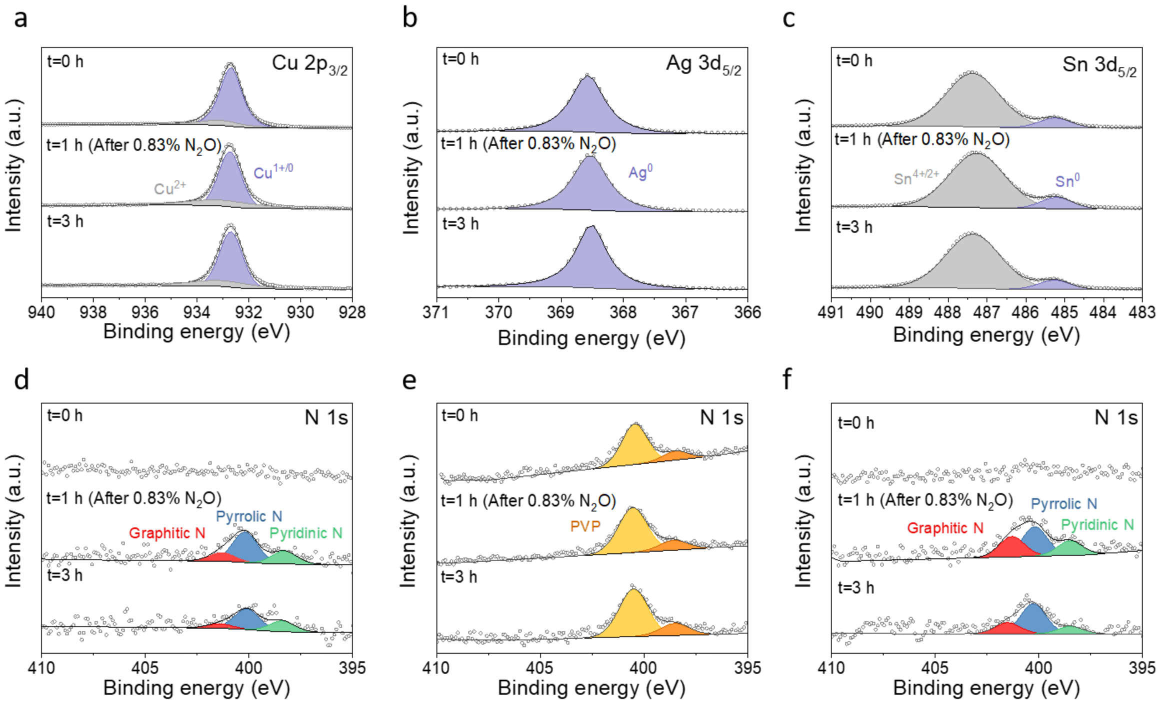

Supplementary Figure 29. XPS measurements of (a) Cu, (b) Ag, and (c) Sn electrodes before exposure to 0.83% N_2O (t=0 h), after exposure to 0.83% N_2O (t=1 h), and after 3 h electrolysis (t=3 h) from a 100 mA cm^{-2} constant current CO_2RR experiment with the introduction of 0.83% N_2O. Corresponding N 1s XPS measurements of (d) Cu, (e) Ag, and (f) Sn electrodes. Incorporation of N into GDL is observed on Cu and Sn electrodes. In the case of Ag electrode, the investigation of N incorporation is limited due to the presence of PVP surfactant. Corresponding details are provided in Supplementary Table 17.

Supplementary Table 17. XPS N 1s peak positions and surface nitrogen content (wt %) of Cu, Ag, and Sn samples obtained from a 100 mA cm^{-2} constant current CO_2RR experiment with the introduction of 0.83% N_2O. Sample time refers to the time at which the electrode was taken out of the electrolyzer. N content has been calculated using the following equation: N content = $\frac{\Sigma N\ (wt\ \%)}{\Sigma N\ (wt\ \%) + metal\ (wt\ \%)} \times 100$, where ΣN (wt %) = graphitic N (wt %) + pyrollic N (wt %) + pyridinic N (wt%) or PVP (wt%), and metal = Cu, Ag, or Sn.

	Sample time (h)	Graphitic (eV)	Pyrrolic (eV)	Pyridinic (eV)	PVP (eV)	Total N content (wt %)
Cu	1	401.4	400.1	398.4	-	1.28
	3	401.3	400.2	398.3	-	0.89
Ag	0	-	-	-	400.5, 398.5	2.72
	1	-	-	-	400.6, 398.6	2.39
	3	-	-	-	400.5, 398.5	1.88
Sn	1	401.3	400.2	398.5	-	1.02
	3	401.4	400.2	398.4	-	1.46

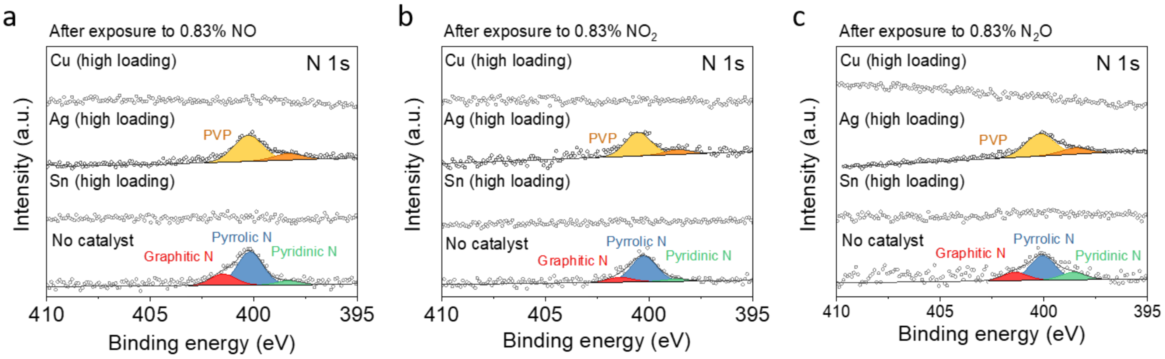

Supplementary Figure 30. XPS measurements of electrodes with high catalyst loadings of 2.0 mg cm^{-2} Cu, Ag, and Sn, and no catalyst at t=1 h after exposure to (a) 0.83% NO, (b) 0.83% NO_2, and (c) 0.83% N_2O for 0.5 h during CO_2 electrolysis. The results confirm that N is incorporated in GDL rather than metal catalysts.

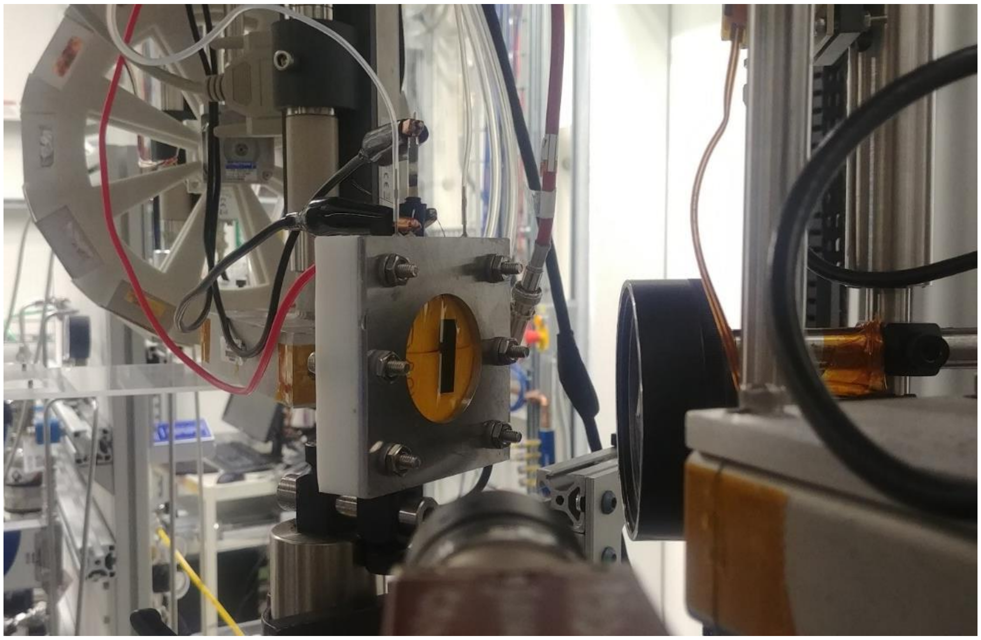

Supplementary Figure 31. Photograph of electrochemical batch cell for XAS operando experiments.

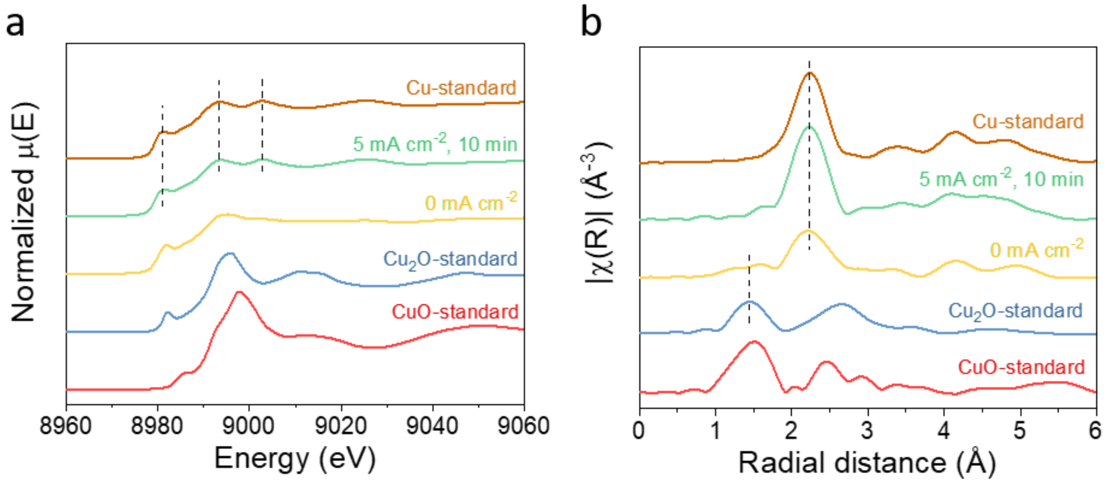

Supplementary Figure 32. Cu K-edge (a) XANES and (f) EXAFS of Cu catalyst obtained after exposure to 0.23% NO_2 for 0.5 h. 0.23% was used instead of 0.83% due to the availability of the gas at the time of the experiment. Nonetheless, insight on the effect of the introduction of NO_2 during CO_2RR on the catalyst oxidation state is still obtained. Cu catalyst is quickly reduced to metallic Cu once current is applied, suggesting that Cu catalyst remains or revert to fully metallic under reaction conditions after NO_2 is removed from the CO_2 stream

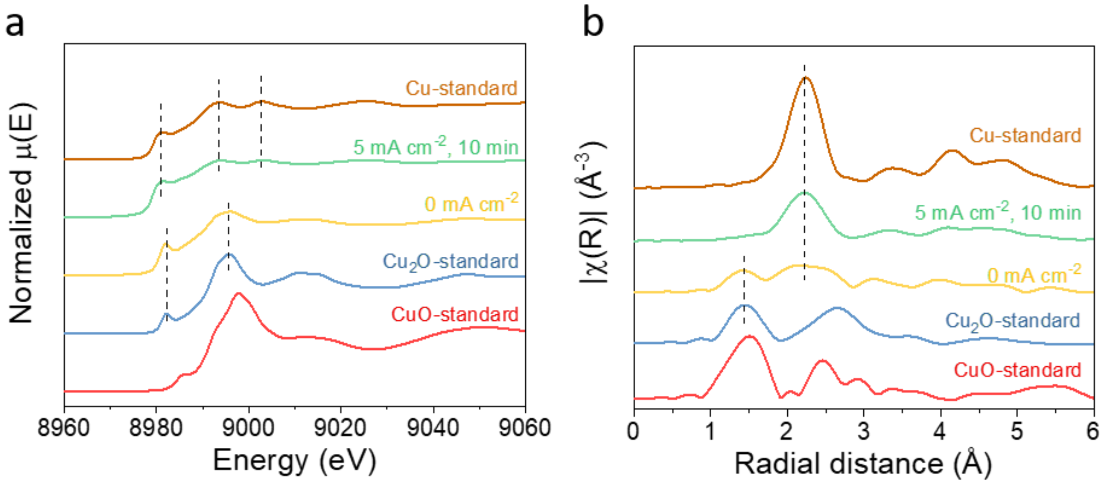

Supplementary Figure 33. Cu K-edge (a) XANES and (f) EXAFS of Cu catalyst obtained after exposure to 0.83% N_2O for 0.5 h. Cu catalyst is quickly reduced to metallic Cu once current is applied, suggesting that Cu catalyst remains or revert to fully metallic under reaction conditions after N_2O is removed from the CO_2 stream

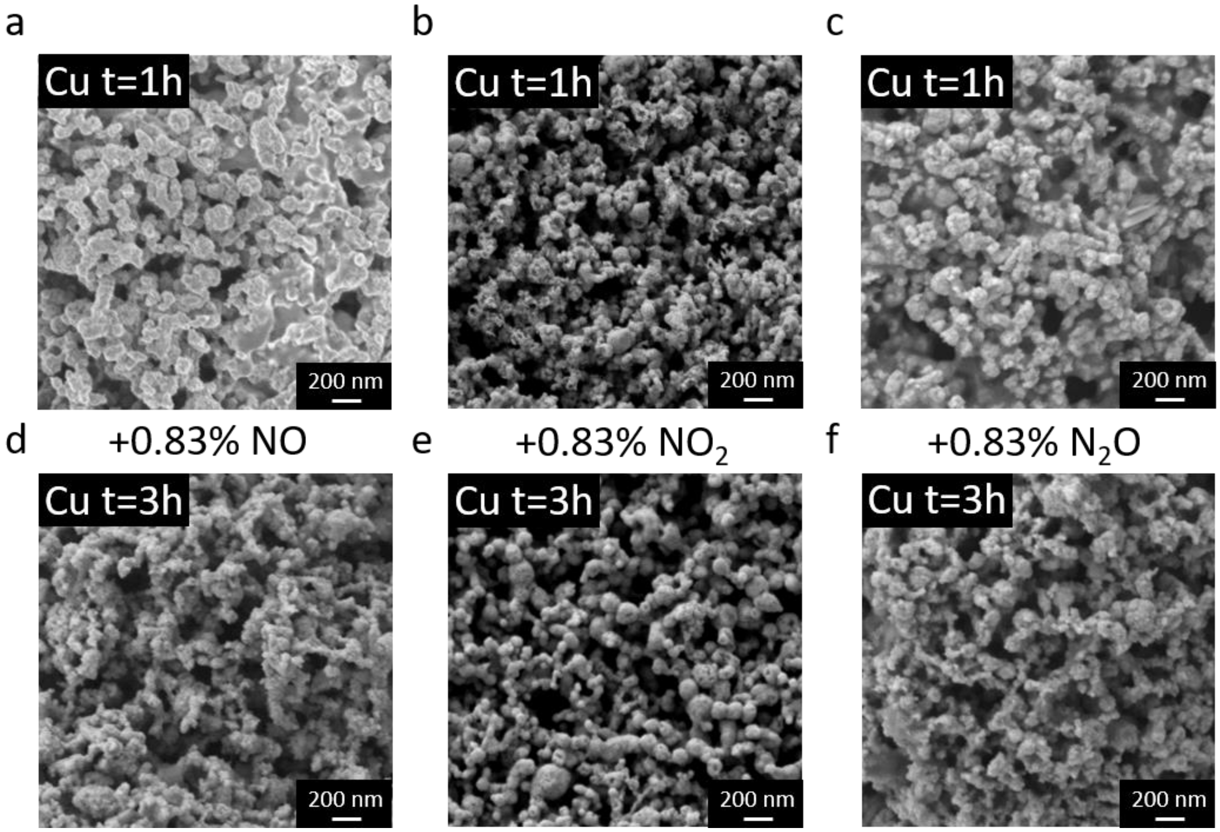

Supplementary Figure 34. SEM images of Cu electrodes obtained at (a) t=1 h and (d) t=3 h after exposure to 0.83% NO, (b) t=1 h and (e) t=3 h after exposure to 0.83% NO_2, and (c) t=1 h and (f) t=3 h after exposure to 0.83% N_2O during CO_2 electrolysis. Change in Cu catalyst obtained at t=1 and 3 h compared to that obtained at t=0 h (Supplementary Fig. 1) is negligible.

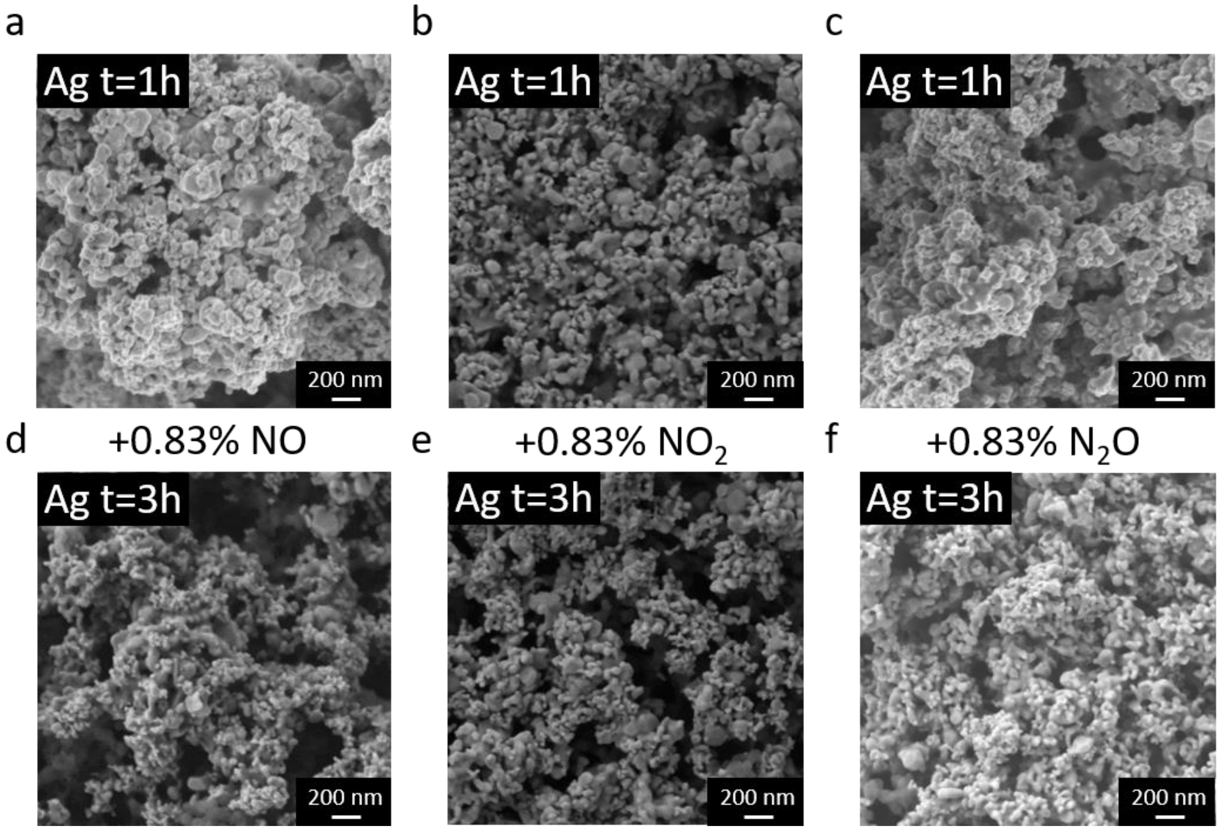

Supplementary Figure 35. SEM images of Ag electrodes obtained at (a) t=1 h and (d) t=3 h after exposure to 0.83% NO, (b) t=1 h and (e) t=3 h after exposure to 0.83% NO_2, and (c) t=1 h and (f) t=3 h after exposure to 0.83% N_2O during CO_2 electrolysis. Change in Ag catalyst obtained at t=1 and 3 h compared to that obtained at t=0 h (Supplementary Fig. 1) is negligible.

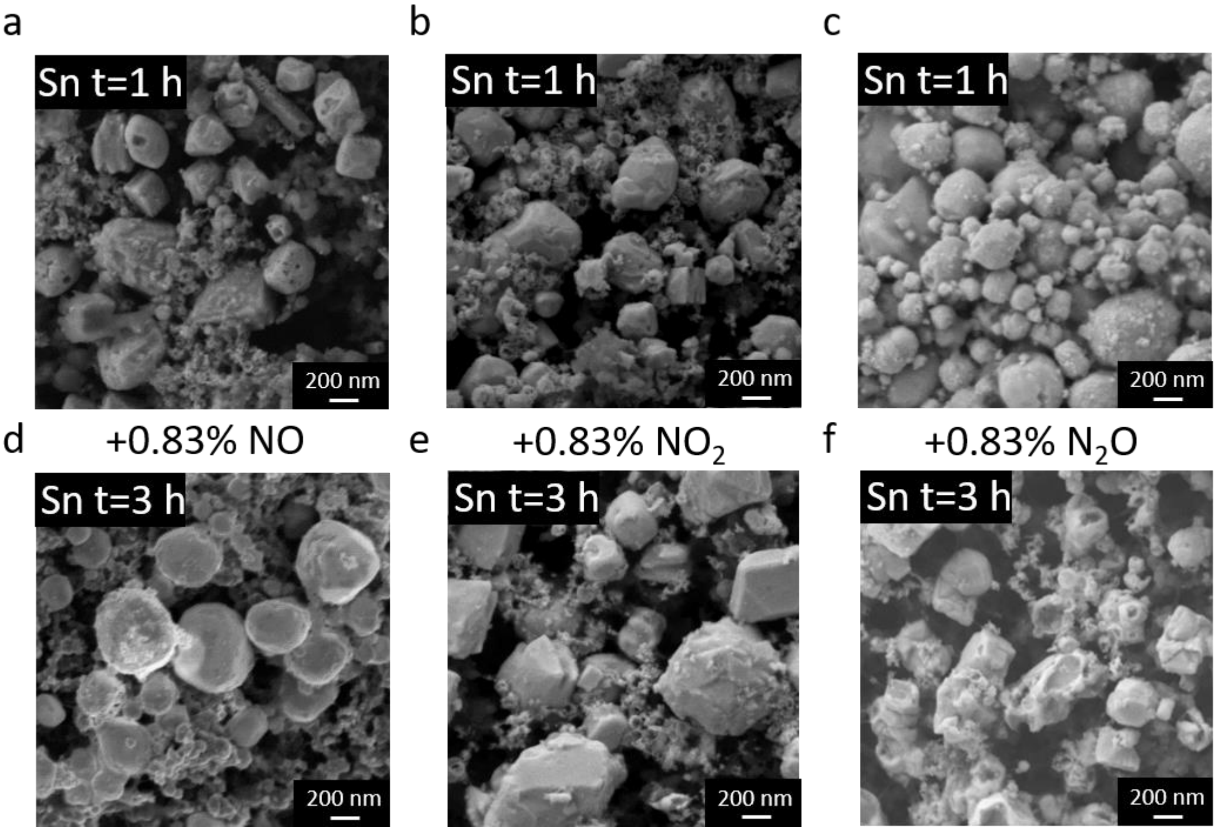

Supplementary Figure 36. SEM images of Sn electrodes obtained at (a) t=1 h and (d) t=3 h after exposure to 0.83% NO, (b) t=1 h and (e) t=3 h after exposure to 0.83% NO_2, and (c) t=1 h and (f) t=3 h after exposure to 0.83% N_2O during CO_2 electrolysis. The particle size for Sn catalyst obtained at t=1 and 3 h increased noticeably compared to that obtained at t=0 h (Supplementary Fig. 1)

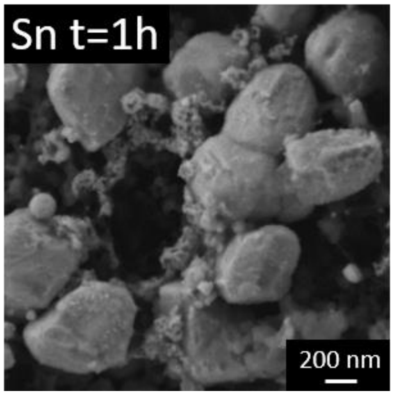

Supplementary Figure 37. SEM image of Sn electrode obtained at t=1 h after CO_2RR without NO_x impurity. Because the particle size increased after CO_2RR without NO_x impurity, NO_x impurities are unlikely the cause of the size change.

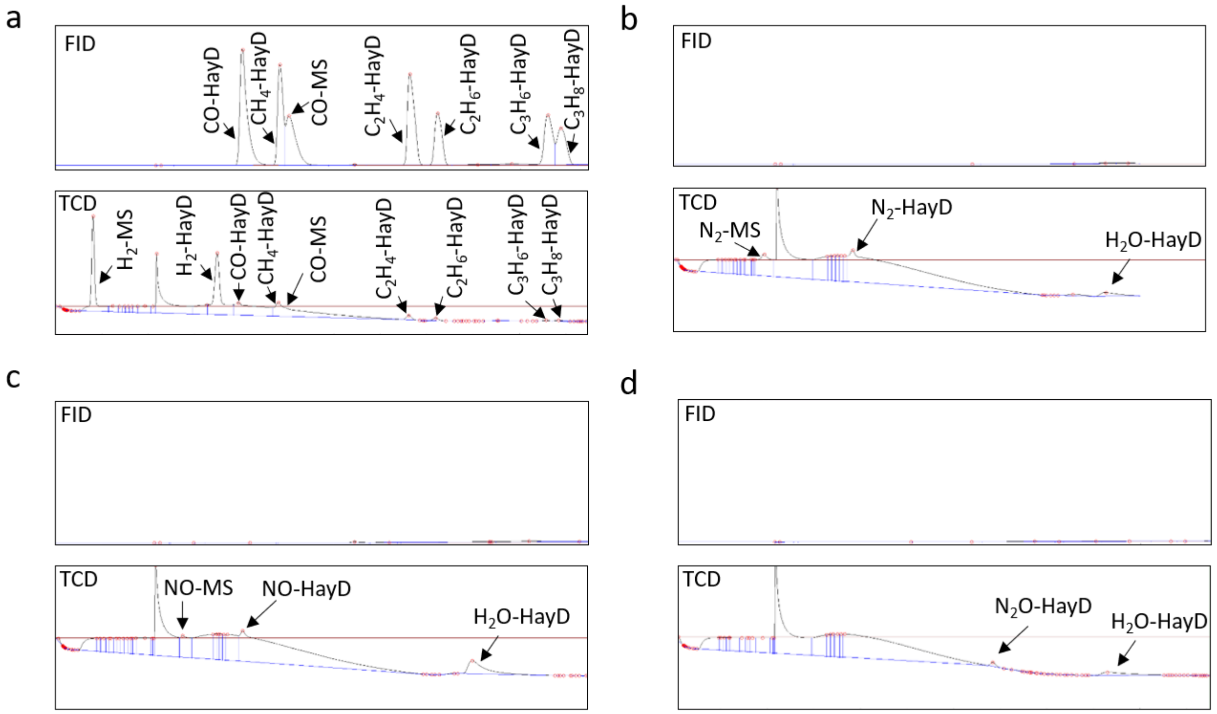

e

Component	Time (min)	Component	Time (min)	Component	Time (min)	Component	Time (min)
H_2-MS	0.81	N_2-HayD	3.85	CO-MS	4.95	H_2O-HayD	9.16
N_2-MS	1.92	CO-HayD	3.94	N_2O-HayD	6.83	C_3H_6-HayD	10.57
NO-MS	2.94	NO-HayD	4.05	C_2H_4-HayD	7.61	C_3H_8-HayD	10.84
H_2-MS	3.48	CH_4-HayD	4.80	C_2H_6-HayD	8.19		

Supplementary Figure 38. Chromatogram of (a) 2% H_2, 1% CO, 1% CH_4, 1% C_2H_4, 0.50% C_2H_6, 0.25% C_3H_6, 0.25% C_3H_8 in Ar, (b) 1% N_2 in Ar, (c) 1% NO in Ar, and (d) 1% N_2O in Ar. Top and bottom are measured by FID and TCD, respectively. (e) Retention time of different components in TCD during GC analysis. Molecular sieve 5 A (MS) and Haysep D (HayD) columns are used for separation of gases.

Supplementary References

1. Rosca, V., Duca, M., DeGroot, M. T. & Koper, M. T. M. Nitrogen Cycle Electrocatalysis. *Chem. Rev.* **109**, 2209–2244 (2009).

2. Bratsch, S. G. Standard Electrode Potentials and Temperature Coefficients in Water at 298.15 K. *J. Phys. Chem. Ref. Data* **18**, 1–21 (1989).

3. Jouny, M., Luc, W. & Jiao, F. General Techno-Economic Analysis of CO_2 Electrolysis Systems. *Ind. Eng. Chem. Res.* **57**, 2165–2177 (2018).
