# Supplementary material for: The impact of nitrogen oxides on electrochemical carbon dioxide reduction
Source: Nat Commun. 2020 Nov 17;11:5856. doi: 10.1038/s41467-020-19731-8 (PMC7672067; doi:10.1038/s41467-020-19731-8)
Supplement: Supplementary file 2 — Peer Review [file 41467_2020_19731_MOESM2_ESM.pdf]

Reviewer #1 (Remarks to the Author):

In this work, Byung Hee Ko et al. investigate the role of nitrogen oxides (NOx) on the selectivity and cell voltage of a carbon dioxide reduction cell. The impacts of 3 gas impurities (NO, NO2, and N2O) in the CO2 stream are measured independently. Three model catalysts (Cu, Ag, and Sn) are used to evaluate the performance changes for each metal. The authors show a reduction in selectivity towards CO2R products and the increase of N2O, N2, and NH3 electroproduction. These nitrogen-containing products are quantified by flow electrochemical mass spectrometry. After the impurities are removed, the selectivity towards CO2R is re-established. The metal catalysts are determined to contain no traces of nitrogen using XPS, operando XANES, and EXAFS.

Comments:

1. Linking to past findings. Remove the sentence from the introduction "Understanding the behavior of the NOx impurities in CO2R is of great importance for practical CO2 electrolysis devices, but it has not been explored yet." Replace it with a more fulsome discussion of past work and key findings including the details of your reference 18, Zhai et al. ECS Transactions (2009), and Komatsu et al. [Preparation of Cu-solid polymer electrolyte composite electrodes and application to gas-phase electrochemical reduction of CO2, Electrochimica Acta (1995)], and other relevant studies citing those. Frame the contribution here more clearly in that context.
2. Conduct further analyses of mass transporting limiting partial current towards NOxR as a function of gas flow rate. How much of the NOx is reduced and how much is left unreacted? Is the loss of current towards CO2R products disastrous even at low gas flow rates? This analysis need not be complex. For instance, a simple calculation could show the amount of current lost to NOxR if all of the NOx that enters the cell is reduced. I would encourage more thinking and analysis along these lines, and some contextualization of the scale of this loss for typical systems and typical impurity containing streams.
3. Discuss the effect of NO2 on HER. Tables S5, S6, and S7 show that the HER is eliminated for all catalysts in the presence of NO2. It would be very interesting to mechanistically understand this phenomenon and whether it can be applied to reduce HER in typical CO2R systems. Moreover, how does the cost of NOxR (see 2 above) change if an offsetting benefit of HER suppression is achieved, or could be achieved. The mechanism here – although not the main focus of the paper – is intriguing and worthy of discussion.
4. Why does the addition of the NOx have a negligible influence on the cell voltage during the constant current experiments? This disagrees with the CVs, which show a large influence on the cell voltage, especially when 0.3% NO2 is added.
5. What are the implications of N-C bonds in the GDL from your XPS investigation? If there are no implications, consider removing some of the panels of Figure 5.
6. Include additional discussion on strategies to avoid the detrimental effects of the lost current towards CO2R (or whether the value of the ammonia, including separation offsets the decreased CO2R selectivity).

Overall, this work builds on previous studies on the impact of NOx impurities contained within CO2 feedstocks on the CO2R selectivity by using more relevant gas diffusion electrodes. I appreciate the work and the focus on practical incoming streams with impurities. Although significant improvements are called for above, I remain upbeat that a suitably revised paper would warrant publication in Nature Communications.

Reviewer #2 (Remarks to the Author):

The manuscript by Ko et al. presents an important quality study on the effects of NOx contaminants in the CO2 feed gas on the performance of three benchmark catalysts for the carbon dioxide electroreduction. The work is generally well designed and presented, though the reviewer still has several scientific questions/suggestions detailed below.

More importantly, the level of innovation of this study does not seem to match the expectations for a paper to be published in the Nature Communications journal. The reviewer would probably change their opinion if the authors introduced a technologically simple solution for the elimination of the pernicious NOx effects (e.g. through gas-liquid scrubbing) and demonstrated its efficient implementation through sufficiently long-term experiments (days). Otherwise, the reviewer can only recommend the publication of the manuscript by Ko et al. in a specialised journal, not Nature Communications.

Other comments:
1| The reviewer could not identify any data on the reproducibility of the results. A top-quality study cannot be based on single runs of each type of experiments – reliability should be demonstrated through consistent results derived from several repeats and presentation of all key data with an appropriately quantified errors attached.
Providing values reported to 3 significant figures preceded by "approximately" is not appropriate as well.

2| It is not clear how introduction of 1% NO and 0.3 % NO_2 into CO_2 stream was achieved using 5% NO in Ar and 1% NO_2 in Ar. Does this mean that actual gas phase composition during the periods when NOx compounds were present were 80% CO_2 + 19% Ar + 1% NO and 67% CO_2 + 32.7% Ar + 0.3 % NO_2, not "1% NO, 0.3% NO_2, … in CO_2"? If this was the case, the reviewer would strongly recommend undertaking relevant control experiments with 80% CO_2 + 20% Ar and 67% CO_2 + 33% Ar mixtures to demonstrate that the decrease in the CO_2 reduction rate is actually exclusively due to the presence of NOx.

3| Moreover, control experiments with no NOx present are anyway required to confirm that the detected continuous deterioration of the performance for all tested materials is the inherent property of each system, not the effect of nitrogen oxides.

4| In XPS, did the new nitrogen signals decrease in intensity after NOx were removed, or remained constant?
Additionally, the reviewer cannot agree with the background (and therefore the overall) fitting of the N 1s XPS data, at least in the way these results are currently presented. Why the background appears like a tangent to the experimental data, rather than an actual fit (i.e. goes approximately in the middle of the noise)?

5| It would be more convenient if potentials were recalculated to the RHE scale to facilitate comparisons with the data in Fig. 1.
Additionally, a bit more details on the IR correction procedure would be desirable: (i) how the Ru values derived from the not highly reliable current-interrupt method compared to a more precise EIS measurements? (ii) were the potential values manually post-corrected (this is not quite clear)?

6| The GC analysis of N_2 is mentioned, but the reviewer could not see any relevant experimental data. Additionally, proper level of details on this analysis should be provided in experimental (temperature gradient, gas carrier / flow rate, sample volume, standard gas mixtures, etc.).

7| The level of details provided for the physical characterisation is unacceptably short and is often limited to the model of the instrument employed. Sample preparation, handling and mounting procedures, conditions during analysis, key instrumental parameters, data analysis protocols – all of these should be explained for every method employed.

Minor:
8| A conceptual comment on the statement "greenhouse gas CO_2, which is independent of fossil sources, such as coal, oil, and natural gas.": this does not seem to connect well to the problem of NOx

and other admixtures in CO2, i.e. the fact that the CO2RR technology would be most useful when coupled to the existing fossil fuel plants. If CO2 is not coming from these plants, the mechanisms for NOx and SOx contamination need to be better explained.

9| Electrochemical half-reaction terminology always includes "R" in the end standing for reaction(s). Although "CO2R", "N2OR" and "NO2R" might not cause too much confusion, the acronym "NOR" actually stands for the nitrogen oxidation reaction. Overall, conversion of all "XR" to "XRR" is recommended.

10| "electron efficiency" is quite a strange term that does not help explaining what faradaic efficiency is. To be confident that the reviewer's memory does not fail them, classical electrochemistry textbooks and Bard's electrochemical dictionary have been checked before writing this comment.

11| Tables S5-S7 – why Total value is "n/a"? Besides, it might be more informative to use < [LOD value], rather than unexplained "n/a" abbreviation for the components that were not detected (if the reviewer interprets these data correctly).

12| XPS spectra = X-ray photoelectron spectroscopy spectra = salty salt.

13| Line 273: "1 cm-2" should be fixed.

14| Line 302: "um" should be "μm".

Reviewer #3 (Remarks to the Author):

Summary:
The article submitted for publication by Ko and coworkers examines the impact of NOx impurities upon electrochemical CO2 reduction. The authors study Cu, Ag, and Sn, which are highly active CO2 reduction catalysts for C2+ products, CO, and formate, respectively. The authors find that of the impurities NO, NO2, and N2O, it is primarily NO and NO2 which negatively but reversibly impact the performance of high-rate GDE flow cell systems for CO2 electrolysis. The results of the study indicate that when NOx impurities are added to the gas feed stream during CO2 reduction, FE for CO2 reduction decreases due to the competing NOx reduction reactions, which in the case of NO reduction largely produces NH3 (as well as some N2O on Cu), and in the case of N2O reduction largely produces N2 (except over Sn where N2O is not easily reduced). Catalytic performance of CO2 reduction bounces back, however, when the NOx impurities are removed. The authors argue based on catalyst characterization data that the presence of NOx during electrolysis results in nitridation of the carbon paper catalyst support, while the metal catalyst particles are unaffected, in part due to the reductive potential of the cathode.

This is an impactful and important paper for the field of CO2 reduction in that it evaluates a crucial practical aspect of high-rate CO2 electrolysis that will influence practical implementation, while taking a basic science perspective to this question. The catalysts chosen for study, as well as the conditions of the study (i.e. a high-rate flow cell setup), were well-selected for practical applicability. However, there are some questions which should be addressed by the authors to strengthen their work and clarify both their procedures and conclusions. These questions and suggestions are grouped below:

1. Site blocking:
What are the authors' conclusions or next hypotheses based on the competition effect that they observe? I'm particularly interested in distinguishing the "competing reaction" versus the "site-blocking" depiction, which is shown in figure 1A. Clearly the reactions are occurring concurrently, which is what the first panel shows. But based on Figure 2, there is no distinct change in cathodic operating potential as NO is added to the feed stream. A decrease in CO2R FE at constant current

means the CO_2 partial current has decreased. This happening while potential is held more or less constant would imply competition for active sites, or "site blocking," not necessarily brought about by catalyst poisoning but by high coverage of active sites which are dynamically exchanging substrates. What do the authors think about this? Do they have an alternative explanation for why partial current for CO2R would decrease at a constant potential? (Note: if potential is not roughly constant, then the potential axis on Figure 2 needs to be re-scaled to accurately depict changes.)

2. Transport:
a. The argument that N_2O is less reactive than NO_2 or NO is at least partially predicated on the notion that all of these reactions are under kinetic control at the conditions of interest – is this the case? I drew up some math for a flooded system that wouldn't apply to a GDE, but I'll present it here anyway: NO as an example is 18x less soluble than CO_2 with concentration in water of around 2 mM at 25 C saturation. So 1% NO gas would result in a 0.02 mM NO solution. In flooded configuration one would not expect higher rates than hundreds of micro-Amps at most. By what factor can we expect transport-limited currents to increase in the GDE configuration, especially given the catalyst-flooding argument that the authors present?
b. The above comment could be addressed experimentally fairly easily. A good place to start is with cyclic voltammetry. Fig. S3 is interesting – I'd be curious to see what the CV would look like with 1% NO in an inert balance (N_2 or Ar). One would expect with the large difference in standard reduction potential between NO and CO_2 that the onset of NOR might be significantly positive of CO2R. It's hard to tell from these plots what the NOR looks like on its own, even if we just assume that the currents are purely additive. Is it possible that even in this GDE setup, because NO is so dilute, the reaction could be reaching transport limitations prior to CO2R onset? Especially on Ag it appears that NOR sort of "plateaus" at around -0.7 V vs. RHE at a current density of ~10 mA/cm2. It's hard to tell without the NO-only CV.

3. Gas composition choices & procedures:
a. Methods: Flow Cell Electrolysis – this section says that stock gases were already dilute: NO stock was 5% in Ar and NO_2 was 1% in Ar. This means that a large portion of the inlet gas consisted of Ar. Can the authors speak to how they kept the partial pressure of CO_2 constant in the electrolysis cell across all conditions? This is important, because unless this constant-CO2-pressure condition was met, the decrease in CO2R FE could be attributed merely to lower CO_2 partial pressures rather than to the interference of NOx as the authors claim. (Note that constant mass flow does not equate to constant pressure!)
b. I'm intrigued by the authors' choice to use 0.3% NO_2 while the other gases are present at 1%. Why the distinction? It seems that even if the percentage NOx component used is indicative of its concentration in industrial flue gas, we would gain more by comparing the gases in equal quantity to normalize for gas availability. The authors should justify their decision.
c. In all of the cases where FEMS was used, the authors have justified their use of 1% NO in Ar rather than in CO_2. It appears that the total current density applied during these tests was the same as that applied in the experiments with 1% NO in CO_2. It would seem that the potential required in order to achieve this current would be greater in the case of 99% inert gas than it would be in the case of fully reactive gas. What is the cathode potential during these tests without CO_2? If the cathode has to be held at a higher potential in order to achieve the same current density, then is not the NOR facing a different overpotential? How then are the results in the presence of 99% Ar translatable to a system with 99% CO_2, when the catalytic system used to interrogate CO_2 reduction response to NOx and the one used to interrogate NOx reduction products both contain different gases and are held at different potentials?
d. NO_2 hydrolyzes in water to generate nitric acid and nitrous acid. To what extent does the gas dissolve into the electrolyte? Could this affect the catalyst? Is this effect dampened by the fact that the electrolyte is constantly replenished?

4. N_2O as a less-reactive species:
a. Line 133: the authors claim that CO_2R on Sn catalyst is not affected by the presence of N_2O because Sn is a bad N_2O catalyst, then provide a citation. However, in the citation it appears that at -1.7 V vs. Ag/AgCl (a potential within the range of CVs in the present work's SI), Sn catalyzes N_2O reduction to N_2 with ~70% FE at a total current of 8 mA/cm2, and this was in a flooded electrode configuration, although at 100% N_2O. Is the explanation that the current toward N_2O reduction is small in comparison to the 100 mA/cm2 current being drawn by CO_2 reduction? Could concurrent CO_2 reduction actually diminish the amount of N_2O reduction occurring? It would be nice if the authors could give their thoughts.
b. Lines 143-145 suggest that Cu is a good NOx reduction catalyst and point to this as a direction for future study. It is worth pointing out that citation 23 mentioned above does actually include Cu as one of the best-performing N_2O reduction catalysts, achieving high FEs for N_2O to N_2 at relatively low overpotentials.

5. Catalyst oxidation state:
It seems that the factors of oxidation by NOx and reduction by potential are competing here. The authors have shown that pre-treated catalysts will revert back to metal under reductive potential, but this does not necessarily mean that this is the case even when the catalyst is being actively exposed to NOx. It seems that in-operando experiments would be most useful here to definitively show the oxidation state of the catalyst. However, lacking sure evidence, I would tend to form a hypothesis in agreement with the authors' conclusions – it would not surprise me if under the strongly reductive potentials required for CO_2R, the catalysts were fully metallic.

6. FE closure:
Can the authors explain why their FE closure is sub-90% in all cases (and sub-70% in some) even with a pure CO_2 feed? At such high currents there should be little issue detecting products. Can the balance be attributed to product crossover to the anolyte?

7. Transient selectivity changes:
The authors claim that increases in HER (and if I may add from my own interpretation, formate – which tends to be an "outer-sphere" CO_2R product) over time are due to GDE flooding, based on a previous paper of theirs. Do we know in this instance whether this holds true? For instance, what is the 3-hour CO_2R baseline (no NO added)? Could transient changes (e.g. increase in HER FE) be attributed to metal plating? This is not a huge priority, since transient changes are not on a scale that derails the central argument of the paper.

8. General comments on methods and SI:
a. Could the authors provide some supporting data or at least a comprehensive reference on their Nessler's method calibration curves, exact procedure, etc.?
b. The choice to detect NOR products via FEMS is interesting. It seems that the drawback mentioned by the authors in the SI is significant: simultaneous NOR and CO_2R are difficult to study. Is it not possible to use in-line gas chromatography to detect N_2 and N_2O, as was done during N_2O reduction?
c. Could the authors provide a more thorough SI explanation of the MS processing techniques used? It is currently a bit difficult to follow the logic behind these steps. After reading for quite some time I eventually ascertained that, for example, Figure S9 represents the raw data for NOR on Cu; Figure S10 shows how background contributions from water and CO_2 are subtracted from the m/z = 17 and 44 peaks respectively; Figure 4c shows the background-subtracted data for these two m/z and additionally m/z = 30 which contains both NO and N_2O, but which can be distinguished by accounting for the N_2O area based off the m/z = 44 peak as well as the ratio of peaks in the pure N_2O spectrum;

and Figure 4d combines all of this information to show how the m/z = 14 peak can be broken down into contributions from NO consumed, NH3 produced, and N2O produced. A description to this end would be useful.
d. The authors should provide a sample MS of CO2 in Figure S11 if they're going to be using the CO2 spectrum to process data.
e. The authors argue that the residual of the m/z = 14 peak being near-zero is sufficient to say that no N2 is produced. While I agree this should be decently good evidence for that, could we not tell this more directly (i.e. with fewer contributions to subtract out) by examining the m/z = 28 peak, which only appears to contain contributions from N2 and N2O? Are results obtained in this manner consistent with results obtained using the authors' approach?
f. Can the authors detail the procedure for XPS and XAS preparation? Were the catalysts exposed to air in the process of transferring from electrolysis cell to XPS for analysis? If so, how long did this take? Similarly, how long before XAS were the catalysts exposed to NOx? In what environment were they kept in the interim?
g. Nessler's reagent is prone to significant errors in quantification and the indophenol method should be used instead to confirm results

9. Small corrections and typos:
a. I would recommend including Engelbrecht et al. 2017 (https://doi.org/10.1016/j.electacta.2016.12.059) in the literature review – the specific goal of this paper was to oxidize the Cu catalyst by co-feeding O¬2 during CO2 reduction (second effect depicted in your Fig. 1A).
b. Two tables are listed as "Table S1" in the supporting information
c. Line 188: it's probably best to remind the reader in this spot what the loss of CO2R FE was during these tests so they don't have to go back and look to check that this is a true statement.
d. Table S11 caption: could the authors clarify what they mean by "N content has been calculated relative to metal content"? I would assume this means that the percentage column is equal to 100% times the ratio N/(N plus the nominal metal), but want to clarify.
e. Methods line 266: I'm guessing "Sn (0.1 m, Alfa Aesar)" really means 0.1 um rather than 0.1 m. Those are some big particles if not! :)
f. Line 305-306: SEM here refers to secondary electron multiplier – I might suggest using terminology which corresponds to the acronym so that it's not confusing.

This is an important contribution to the literature - if the revisions above are made, I would advocate for accepting the article.

RESPONSES TO REVIEWER COMMENTS

Reviewer #1 (Remarks to the Author):

In this work, Byung Hee Ko et al. investigate the role of nitrogen oxides (NOx) on the selectivity and cell voltage of a carbon dioxide reduction cell. The impacts of 3 gas impurities (NO, NO2, and N2O) in the CO2 stream are measured independently. Three model catalysts (Cu, Ag, and Sn) are used to evaluate the performance changes for each metal. The authors show a reduction in selectivity towards CO2R products and the increase of N2O, N2, and NH3 electroproduction. These nitrogen-containing products are quantified by flow electrochemical mass spectrometry. After the impurities are removed, the selectivity towards CO2R is re-established. The metal catalysts are determined to contain no traces of nitrogen using XPS, operando XANES, and EXAFS.

Comments:

1. Linking to past findings. Remove the sentence from the introduction "Understanding the behavior of the NOx impurities in CO2R is of great importance for practical CO2 electrolysis devices, but it has not been explored yet." Replace it with a more fulsome discussion of past work and key findings including the details of your reference 18, Zhai et al. ECS Transactions (2009), and Komatsu et al. [Preparation of Cu-solid polymer electrolyte composite electrodes and application to gas-phase electrochemical reduction of CO2, Electrochimica Acta (1995)], and other relevant studies citing those. Frame the contribution here more clearly in that context.

Reply:

We thank the reviewer for the constructive comments. The following discussion has been added to the manuscript and the work by Komatsu et al. has been cited as reference #18:

> "Previous study has shown that 200 ppm of NO has a negligible influence on Cu catalysts in CO_2RR in a conventional batch cell.[18] Furthermore, less than or equal to 1667 ppm of NO_2 has shown to be either beneficial or neutral, and greater than 1667 ppm of NO_2 has shown to be detrimental in CO_2RR, mainly due to a reduction in pH of the electrolyte, also on Cu catalysts in a conventional batch cell.[19] However, the behavior of various NO_x impurities in CO_2RR at industrially relevant high current densities (>100 mA cm^{-2}) has not been explored yet." (Page 2, Lines 50-56)

2. Conduct further analyses of mass transporting limiting partial current towards NOxR as a function of gas flow rate. How much of the NOx is reduced and how much is left unreacted? Is the loss of current towards CO_2R products disastrous even at low gas flow rates? This analysis need not be complex. For instance, a simple calculation could show the amount of current lost to NOxR if all of the NOx that enters the cell is reduced. I would encourage more thinking and analysis along these lines, and some contextualization of the scale of this loss for typical systems and typical impurity containing streams.

Reply:

We have evaluated the influence of NO in CO_2RR at two additional concentrations of 0.083%, representing the typical NO_x concentration in flue gases, and 0.0083%, representing the typical NO_x concentration after the NO_x removal process (**Fig. R1**). Although the losses in

CO_2RR FE at 0.83% were detrimental, the effect was less severe at 0.083% NO, with less than 5% loss in CO_2RR FE, and negligible at 0.0083% NO. Therefore, we conclude that NO at typical concentrations of NO_x in flue gases is compatible with CO_2RR, although a complete removal of NO_x is desired to maximize CO_2RR FE.

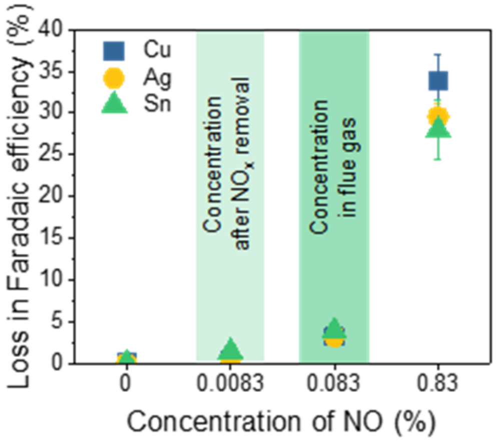

Figure R1 (New Figure 2d). Effect of different concentrations of NO in CO_2 electroreduction on Cu, Ag, and Sn catalysts. 0.083% and 0.0083% represent the typical NO_x concentration in flue gases and flue gases after NO_x removal processes, respectively.

In the revised manuscript, we have included the following discussion:

> "Investigation of the effect of different concentrations of NO in CO_2RR shows that NO_x at typical concentrations in flue gases is compatible with CO_2RR." (Page 3, Lines 77 and 79)
>
> "To obtain insight on the influence of NO_x in CO_2RR at typical concentrations of NO_x in point sources, we evaluated the effect of 0.083% and 0.0083% NO, representing the typical NO_x concentration in flue gases and flue gases after NO_x removal processes,[22] respectively, in CO_2RR (Fig. 2d). Although the losses in FE at 0.83% NO were detrimental, the effect of NO was less severe at 0.083%, with less than 5% losses in FE, and negligible at 0.0083% NO. Therefore, NO at typical concentrations of NO_x in flue gases is compatible with CO_2RR, although a complete removal of NO_x is desired to maximize CO_2RR FE." (Page 5, Lines 132-138)

Furthermore, we have calculated the conversion of NO during CO_2RR with the introduction of 0.83% NO, assuming NO fully is converted to NH_3 (**Table R1**). Conversions of NO were 59.1%, 51.5%, and 48.7% on Cu, Ag, and Sn catalysts, respectively.

Table R1 (New Table S5). Conversion of NO during CO_2RR with the introduction of 0.83% NO, assuming NO is fully converted to NH_3. $Conversion\ (\%) = \frac{Current\ loss\ due\ to\ NO}{Current\ required\ to\ fully\ convert\ NO\ to\ NH_3} \times 100$. Full conversion of NO to NH_3 requires 57.4 mA.

Catalyst	Cu	Ag	Sn
Conversion (%)	59.1	51.6	48.7

In the revised manuscript, we have included the following discussion:

"Assuming NO is fully converted to NH_3, conversions of NO during CO_2RR are between 48% and 62% (Table S5)." (Page 4, Lines 117-118)

3. Discuss the effect of NO_2 on HER. Tables S5, S6, and S7 show that the HER is eliminated for all catalysts in the presence of NO_2. It would be very interesting to mechanistically understand this phenomenon and whether it can be applied to reduce HER in typical CO_2R systems. Moreover, how does the cost of NO_xR (see 2 above) change if an offsetting benefit of HER suppression is achieved, or could be achieved. The mechanism here – although not the main focus of the paper – is intriguing and worthy of discussion.

Reply:

We apologize for the confusion. We originally thought that NO_2 produces a peak near H_2 peak in GC, making it difficult to quantify H_2. During the revision, we have confirmed that NO_2 does not overlap with H_2 and accurately quantified H_2 FE for the experiment with NO_2. In the revised manuscript, we have provided the updated H_2 FE in **Tables S7-9**. Similar to the introduction of NO and N_2O, the introduction of NO_2 slightly decreases or has negligible effect on H_2 FE.

4. Why does the addition of the NOx have a negligible influence on the cell voltage during the constant current experiments? This disagrees with the CVs, which show a large influence on the cell voltage, especially when 0.3% NO2 is added.

Reply:

Introduction of NO_x shifts the potential during the constant current experiments, and we have adjusted the scale of the potential axis to show this more clearly (**Fig. 2a-c, S6, and S7**), although the large fluctuation in potentials caused by continuous bubble formation and flush out on the electrode surface makes the shift in potential less evident. Regarding the difference between the constant current experiments and the CV measurements, the CV measurements do not necessarily translate directly to the constant current experiments. We used 50 mV/s as the scan rate for the CV measurements and the intensity of currents in the CV measurements is dependent on the scan rates. Although the trends will remain the same, the intensity will be lower for the slow scan rates and greater for the fast scan rates.

5. What are the implications of N-C bonds in the GDL from your XPS investigation? If there are no implications, consider removing some of the panels of Figure 5.

Reply:

Thank you for the suggestion. In the revised manuscript, we have moved two panels from the main figure to the SI (**Fig. S27 and Fig S30**) and the revised **Figure 5** is shown below (**Fig. R2**).

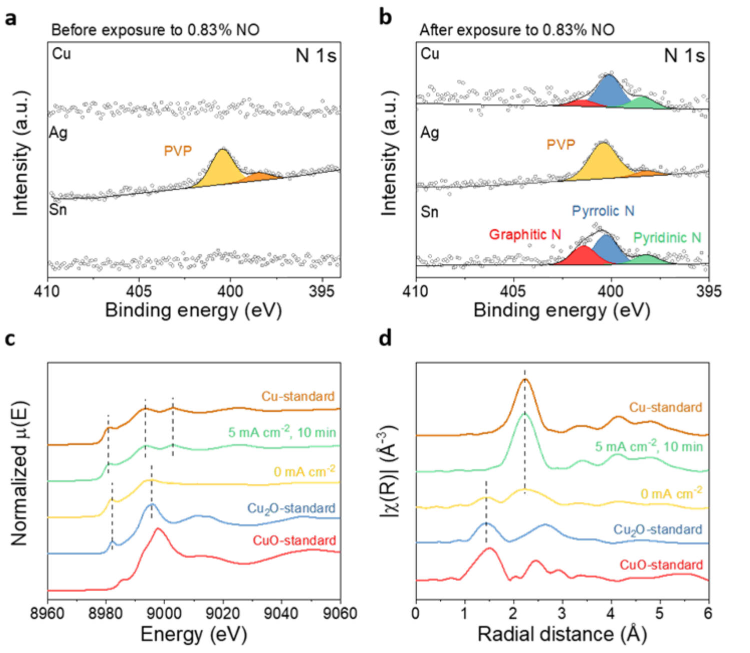

Figure R2 (New Figure 5). Evaluation of the influence of NO on the catalyst structure. XPS measurements of Cu, Ag, and Sn electrodes (a) before exposure to 0.83% NO (t=0 hour), (b) after exposure to 0.83% NO (t=1 hour). Corresponding XPS data is provided in Fig. S18 and Table S11. Cu K-edge (c) XANES and (d) EXAFS spectra of Cu catalyst after exposure to 0.83% NO. Cu foil, Cu_2O, and CuO were used as references.

6. Include additional discussion on strategies to avoid the detrimental effects of the lost current towards CO2R (or whether the value of the ammonia, including separation offsets the decreased CO2R selectivity).

Reply:

As discussed in **Q2 (Reviewer 1)**, we conclude that although high concentrations of NO_x may be detrimental to CO_2RR, NO_x at typical concentrations of flue gases is compatible with CO_2RR, causing small losses of CO_2RR FE without altering the catalyst properties permanently. Integration of NO_x removal process, which is a relatively mature technology, may also be considered to ensure CO_2RR operation at maximum efficiency. In addition, because the loss in FE is relatively small at typical NO_x concentrations in flue gases, production of NORR products, such as ammonia, is too low to be considered as main products.

In the revised manuscript, we have added the following discussion in the Conclusions section:

> "Furthermore, although high concentrations of NO_x may be detrimental to CO_2RR, NO_x at typical concentrations of flue gases is compatible with CO_2RR, causing small losses in CO_2RR FE. NO_x removal process, which is a relatively mature technology, may also be employed to ensure CO_2RR operation at maximum efficiency." (Page 10, Lines 309-312)

Overall, this work builds on previous studies on the impact of NOx impurities contained within CO2 feedstocks on the CO2R selectivity by using more relevant gas diffusion

electrodes. I appreciate the work and the focus on practical incoming streams with impurities. Although significant improvements are called for above, I remain upbeat that a suitably revised paper would warrant publication in Nature Communications.

Reviewer #2 (Remarks to the Author):

The manuscript by Ko et al. presents an important quality study on the effects of NOx contaminants in the CO2 feed gas on the performance of three benchmark catalysts for the carbon dioxide electroreduction. The work is generally well designed and presented, though the reviewer still has several scientific questions/suggestions detailed below.
More importantly, the level of innovation of this study does not seem to match the expectations for a paper to be published in the Nature Communications journal. The reviewer would probably change their opinion if the authors introduced a technologically simple solution for the elimination of the pernicious NOx effects (e.g. through gas-liquid scrubbing) and demonstrated its efficient implementation through sufficiently long-term experiments (days). Otherwise, the reviewer can only recommend the publication of the manuscript by Ko et al. in a specialised journal, not Nature Communications.

We thank the reviewer for the comments. As NO_x removal is a relatively mature technology, we believe the demonstration of the integration of NO_x removal process and CO_2RR system is out of scope of this work. In fact, we could consider the CO_2 electrolyzer itself as a potential solution to remove low concentrations of NO_x. This work is focused on understanding the effect of trace amount of NO_x, common contaminants in flue gases, in CO_2RR, and we provide a comprehensive insight on the effect of NO_x in CO_2RR at commercially relevant high current densities. Given the rapid growth and commercialization activities of the CO_2 electrolysis technology, this work is an important contribution to reveal the potential impacts of NO_x in realistic CO_2 sources. We believe that the reviewer's comments are fully addressed and the revised manuscript is suitable for a publication in Nature Communications.

Other comments:
1| The reviewer could not identify any data on the reproducibility of the results. A top-quality study cannot be based on single runs of each type of experiments – reliability should be demonstrated through consistent results derived from several repeats and presentation of all key data with an appropriately quantified errors attached.
Providing values reported to 3 significant figures preceded by "approximately" is not appropriate as well.

Reply:

In the revised manuscript, all experiments have been repeated three times and error bars have been included (**Figs. 2a-c, 3, 4a, S6, S7, and S12**). The error bars represent the standard deviation of three independent measurements.

We have also removed "approximately" in the manuscript.

2| It is not clear how introduction of 1% NO and 0.3 % NO2 into CO2 stream was achieved using 5% NO in Ar and 1% NO2 in Ar. Does this mean that actual gas phase composition during the periods when NOx compounds were present were 80% CO2 + 19% Ar + 1% NO and 67% CO2 + 32.7% Ar + 0.3 % NO2, not "1% NO, 0.3% NO2, … in CO2"? If this was the

case, the reviewer would strongly recommend undertaking relevant control experiments with 80% CO2 + 20% Ar and 67% CO2 + 33% Ar mixtures to demonstrate that the decrease in the CO2 reduction rate is actually exclusively due to the presence of NOx.

Reply:

We apologize for the confusion. The reviewer is correct that Ar is introduced with NO_x mixture. To eliminate the effect of the change in CO_2 partial pressure during the introduction of NO_x mixture containing Ar, CO_2 partial pressure was controlled throughout the experiment by flowing Ar with CO_2 when NO_x was not required in the gas feed. In essence, the total flow rate of the gas feed was fixed at 19.2 mL min^{-1}. The gas feed was switched from a mixture of 83.3% CO_2 and 16.7% Ar to a mixture of 83.3% CO_2, 15.87% Ar, and 0.83% NO_x, and then back to a mixture of 83.3% CO_2 and 16.7% Ar. We also purchased 5% NO_2 (balanced by Ar) during the revision process to ensure that all NO, NO_2, and N_2O experiments are conducted in the same way for a fair comparison of the effect of different NO_x in CO_2RR.

In the revised manuscript, we included the following explanation:

> "As the concentration of NO_x in typical exhaust streams may be as high as ~3,000 ppm (i.e., 0.3 vol. %),[13] conservative streams of 83.3% CO_2, 15.87% Ar, and 0.83% NO_x were used for most studies. To keep the CO_2 partial pressure constant during the introduction of NO_x, CO_2 partial pressure was maintained at 0.833 bar throughout the study by using a mixture of 83.3% CO_2 and 16.7% Ar when NO_x was not introduced." (Page 3, Lines 89-93)

Furthermore, more detail was added to the methods section:

> "The total gas flow rate was maintained at 19.2 mL min^{-1} with different flow rates of CO_2, Ar (Keengas, 99.999%), and NO_x. For instance, 83.3% CO_2 and 16.7% Ar was prepared by flowing 16 mL min^{-1} CO_2 and 3.2 mL min^{-1} Ar via Brooks GF40 mass flow controllers. The gas mixtures of 83.3% CO_2, 15.87% Ar, and 0.83% NO_x were prepared by flowing 16 mL min^{-1} CO_2 with 3.2 mL min^{-1} of 5% NO/Ar (Matheson Gas) or 3.2 mL min^{-1} of 5% NO_2/Ar (Matheson Gas), respectively, using a 50 mL gastight syringe (1050 SL, Hamilton) via a syringe pump (Cole Parmer). Syringes were quickly switched to another syringe filled with NO or NO_2 before running out of gases. Similarly, 83.3% CO_2, 15.87% Ar, and 0.83% N_2O was prepared by flowing 16 mL min^{-1} CO_2, 3.04 mL min^{-1} Ar, and 0.16 mL min^{-1} N_2O (99.99%, Matheson Gas)." (Page 11, Lines 331-340)

3| Moreover, control experiments with no NOx present are anyway required to confirm that the detected continuous deterioration of the performance for all tested materials is the inherent property of each system, not the effect of nitrogen oxides.

Reply:

We have conducted control experiments with 83.3% CO_2 and 16.7% Ar for 3 hours on all three catalysts (**Fig. R3**). Similar trend of H_2 FE increase over time was observed on all three catalysts, regardless of the introduction of NO_x, supporting that NO_x is not the main cause for the increase in H_2. In the revised manuscript, we have added this figure in **Fig. S5**.

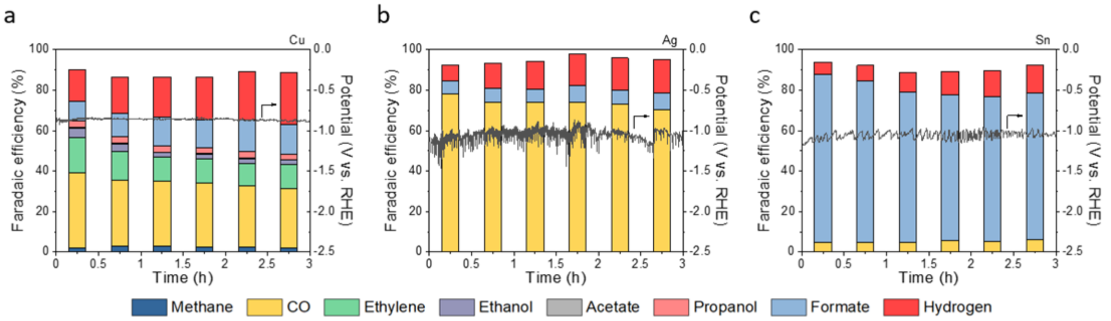

Figure R3 (New Figure S5). Faradaic efficiency and applied potential vs. time with 83.3% CO_2 and 16.7% Ar on (a) Cu, (b) Ag, and (c) Sn catalysts at a constant current density of 100 mA cm^{-2} in 1 M $KHCO_3$ for 3 h. H_2 Faradaic efficiency increases over time due to slow flooding of the electrode, suggesting that NO_x is not responsible for the H_2 FE increase.

4| In XPS, did the new nitrogen signals decrease in intensity after NOx were removed, or remained constant?
Additionally, the reviewer cannot agree with the background (and therefore the overall) fitting of the N 1s XPS data, at least in the way these results are currently presented. Why the background appears like a tangent to the experimental data, rather than an actual fit (i.e. goes approximately in the middle of the noise)?

Reply:

The XPS measurements obtained after 3-hour electrolysis show that the N incorporated in the electrode surface was still intact after additional 2 hour of CO_2RR (Fig. S27 and Table S15), with the total amount of N in the Cu and Sn electrodes remaining relatively unchanged. See Page 8, Lines 249-251.

Regarding the XPS fitting, data points below the background were not shown in the previous data by mistake. In the revised manuscript, all data points below the background are shown and XPS results were fitted again when needed (**Fig. 5a and b, and S27-30**).

5| It would be more convenient if potentials were recalculated to the RHE scale to facilitate comparisons with the data in Fig. 1.
Additionally, a bit more details on the IR correction procedure would be desirable: (i) how the Ru values derived from the not highly reliable current-interrupt method compared to a more precise EIS measurements? (ii) were the potential values manually post-corrected (this is not quite clear)?

Reply:

We have adjusted all potentials to the RHE scale and clearly stated that the potentials are manually post IR-corrected.

We have also compared the resistances of our electrolyzers in 1M $KHCO_3$, measured by electrochemical impedance spectroscopy (EIS) and current-interrupt (CI) (Fig. R4). EIS and CI yielded similar resistance values, with differences of approximately 0.3 ohms. The maximum discrepancy caused by the method at 100 mA is only 30 mV. Liu et al. have also measured the resistances with EIS and CI and showed the reliability of both methods.[1]

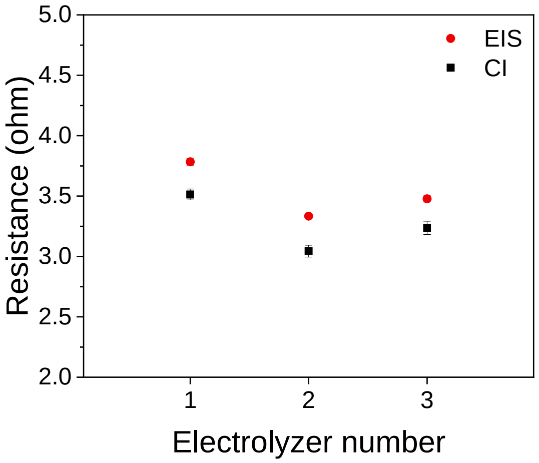

Figure R4. Measured resistance between the working and the reference electrodes of three different electrolyzer units in 1M $KHCO_3$ by EIS and CI.

Reference

1. Liu, K., Smith, W. A. & Burdyny, T. Introductory Guide to Assembling and Operating Gas Diffusion Electrodes for Electrochemical CO 2 Reduction. *ACS Energy Lett.* **4**, 639–643 (2019).

In the revised manuscript, we changed all potentials to the RHE scale (**Figs. 2a-c and S3-11**), clearly stated that the resistance was manually post-corrected, and included the reference for the CI technique.

> "The half-cell potentials were measured with respect to Ag/AgCl reference electrode (Pine Research) and calculated to the RHE scale in which E (vs. RHE) = E (vs. Ag/AgCl) + 0.209 V + 0.0591 V × pH - η_{IRdrop}. The pH was measured at the outlet of the catholyte channel. The resistance was measured with the current-interrupt technique,[40] and the measured potential was manually post IR-corrected" (Page 11, Lines 346-349)

6| The GC analysis of N_2 is mentioned, but the reviewer could not see any relevant experimental data. Additionally, proper level of details on this analysis should be provided in experimental (temperature gradient, gas carrier / flow rate, sample volume, standard gas mixtures, etc.).

Reply:

A typical GC analysis of N_2 during CO_2RR with the introduction of NO and N_2O on Cu, Ag, and Sn catalysts have been provided in **Figs. R5 and 6**, respectively. We have also added details on the GC analysis method in the methods section.

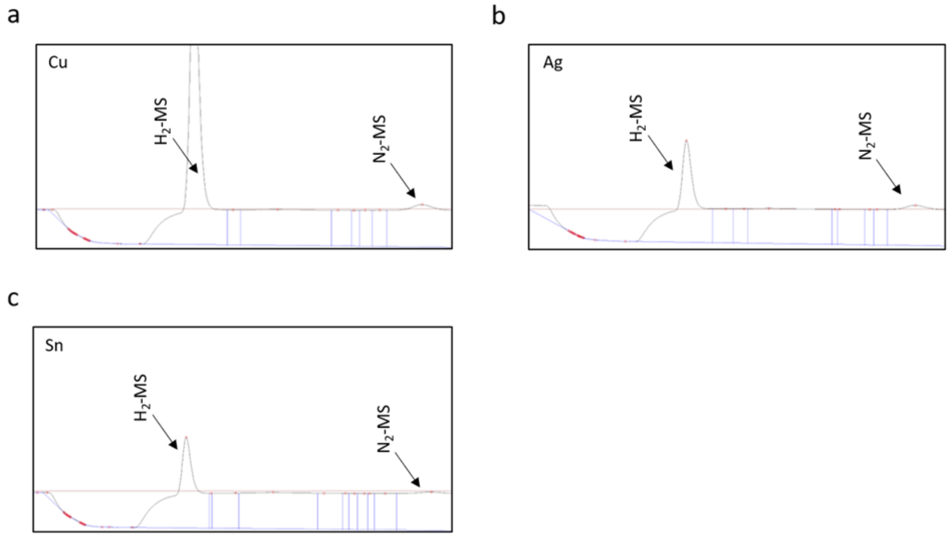

Figure R5 (New Figure S13). Chromatogram of gas products from electrolysis in 83.3% CO_2+0.83% NO in Ar on (a) Cu, (b) Ag, and (c) Sn catalysts at 100 mA cm^{-2}. TCD and Molecular sieve 5A (MS) column were used, and 0 to 2.1 min is shown.

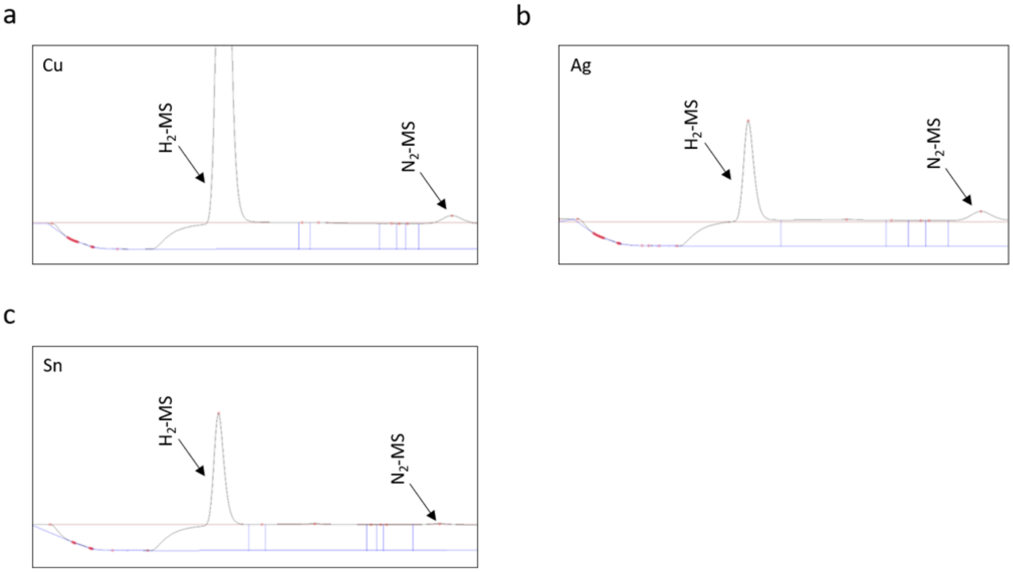

Figure R6. (New Figure S26). Chromatogram of gas products from electrolysis in 83.3% CO_2+0.83% N_2O in Ar on (a) Cu, (b) Ag, and (c) Sn catalysts at 100 mA cm^{-2}. TCD and Molecular sieve 5A (MS) column were used, and 0 to 2 min is shown.

In the revised manuscript, we have added the following details in the methods section:

> "Ar (Keengas, 99.999%) was used as a carrier gas with a flow rate of 19 mL min^{-1} and 1 mL of sample was automatically loaded to the column. The gas sample was loaded to 0.5 m HaySep D pre-column connected to 2 m Molsieve 5A column at 0.050 min. At 0.490 min, any molecule remaining in the HaySep D precolumn was backflushed out to vent. At 2.150 min, gas sample was automatically loaded to 2 m HaySep D column. The column temperature was maintained at 35℃ for 2.950 min, increased to 210℃ at 40℃/min, and maintained at 210℃ until the end of the analysis. A

typical GC analyses of potential CO_2RR products, N_2, NO, and N_2O are provided in Fig. S38. 2% H_2, 1% CO, 1% CH_4, 1% C_2H_4, 0.50% C_2H_6, 0.25% C_3H_6, 0.25% C_3H_8 in Ar (Matheson) was used to obtain the chromatogram of potential CO_2RR products." (Page 11 and 12, Lines 355-363)

7| The level of details provided for the physical characterization is unacceptably short and is often limited to the model of the instrument employed. Sample preparation, handling and mounting procedures, conditions during analysis, key instrumental parameters, data analysis protocols – all of these should be explained for every method employed.

Reply:

We thank the reviewer for the suggestion. In the revised manuscript, we have added more details to the material characterization methods.

"For SEM and XPS measurements, the electrodes were first taken out of the electrolyzer after electrolysis at desired time points. The electrodes were dried in the vacuum oven (MTI Corporation) for up to three days before SEM images were acquired with Auriga 60 CrossBeam (1.5 kV). The electrodes were quickly transported to the XPS equipment (K-alpha Alpha X-ray photoelectron spectrometer system, Thermo Fisher Scientific) after drying in the vacuum oven for 5 minutes. The electrodes were exposed to air for less than 20 minutes. High-resolution XPS measurements were obtained at a pass energy of 20 eV with a step size of 0.1 eV. Flood gun was turned on. Cu 2p, Ag 3d, and Sn 3d were scanned 10 times while N 1s was scanned 30 times. Four different spots were scanned and averaged." (Pages 13, Lines 418-426)

"The electrodes were taken out of the electrolyzer at 1 h (after exposure to NO_x for 0.5 h) during a 100 mA cm^{-1} constant current CO_2RR experiment with the introduction of NO_x (Fig.2a-c, S6 and S7). In the case of NO_2, the samples were exposed to 0.23% NO_2 instead due to the availability of the gas at the time of the experiment. The electrodes were quickly stored in vials filled with Ar and the vials were tightly sealed with Parafilm at the home institution. The electrodes were transported to the Brookhaven National Laboratory (New York, USA) and were loaded into a XAS batch cell, which was fabricated from Teflon and 304 stainless steel, with a Kapton film window for high transmissivity for X-ray measurements (Fig. S31). The electrodes were exposed to air for approximately 20 minutes before the measurement. Pt wire and Ag/AgCl were used as a counter and a reference electrode, respectively. 1M $KHCO_3$ was used as an electrolyte and CO_2 was flowed at 10 mL min^{-1}." (Page 13, Lines 430-441)

Minor:

8| A conceptual comment on the statement "greenhouse gas CO2, which is independent of fossil sources, such as coal, oil, and natural gas.": this does not seem to connect well to the problem of NOx and other admixtures in CO2, i.e. the fact that the CO2RR technology would be most useful when coupled to the existing fossil fuel plants. If CO2 is not coming from these plants, the mechanisms for NOx and SOx contamination need to be better explained.

Reply:

We agree with the review and the statement has been revised. The new sentence now reads as follows:

> "The electrochemical CO_2 reduction (CO_2RR) provides a promising, sustainable avenue to generate value-added fuels and chemicals from greenhouse gas CO_2." (Page 1, Lines 25-26)

9| Electrochemical half-reaction terminology always includes "R" in the end standing for reaction(s). Although "CO2R", "N2OR" and "NO2R" might not cause too much confusion, the acronym "NOR" actually stands for the nitrogen oxidation reaction. Overall, conversion of all "XR" to "XRR" is recommended.

Reply:

We agree that NOR may be interpreted as nitrogen oxidation reaction. All terminologies have been modified to "CO_2RR", "NORR", NO_2RR", and "N_2ORR".

10| "electron efficiency" is quite a strange term that does not help explaining what faradaic efficiency is. To be confident that the reviewer's memory does not fail them, classical electrochemistry textbooks and Bard's electrochemical dictionary have been checked before writing this comment.

Reply:

We have removed the term "electron efficiency" and redefined Faradaic efficiency:

> "… Faradaic efficiency (FE; i.e., number of electrons transferred to desired products divided by the total number of electrons passed in the system) …" (Page 1, Lines 40-41)

11| Tables S5-S7 – why Total value is "n/a"? Besides, it might be more informative to use < [LOD value], rather than unexplained "n/a" abbreviation for the components that were not detected (if the reviewer interprets these data correctly).

Reply:

In the original submission, we did not quantify the amount of H_2. In the revision, the amount of H_2 is now accurately quantified, and the results are provided in Tables S7-8.

12| XPS spectra = X-ray photoelectron spectroscopy spectra = salty salt.

Reply:

The term "XPS spectra" has been modified to "XPS measurements."

13| Line 273: "1 cm-2" should be fixed.

Reply:

The unit has been corrected.

14| Line 302: "um" should be "µm".

Reply:

The unit has been corrected.

Reviewer #3 (Remarks to the Author):

Summary:
The article submitted for publication by Ko and coworkers examines the impact of NOx impurities upon electrochemical CO2 reduction. The authors study Cu, Ag, and Sn, which are highly active CO2 reduction catalysts for C2+ products, CO, and formate, respectively. The authors find that of the impurities NO, NO2, and N2O, it is primarily NO and NO2 which negatively but reversibly impact the performance of high-rate GDE flow cell systems for CO2 electrolysis. The results of the study indicate that when NOx impurities are added to the gas feed stream during CO2 reduction, FE for CO2 reduction decreases due to the competing NOx reduction reactions, which in the case of NO reduction largely produces NH3 (as well as some N2O on Cu), and in the case of N2O reduction largely produces N2 (except over Sn where N2O is not easily reduced). Catalytic performance of CO2 reduction bounces back, however, when the NOx impurities are removed. The authors argue based on catalyst characterization data that the presence of NOx during electrolysis results in nitridation of the carbon paper catalyst support, while the metal catalyst particles are unaffected, in part due to the reductive potential of the cathode.

This is an impactful and important paper for the field of CO2 reduction in that it evaluates a crucial practical aspect of high-rate CO2 electrolysis that will influence practical implementation, while taking a basic science perspective to this question. The catalysts chosen for study, as well as the conditions of the study (i.e. a high-rate flow cell setup), were well-selected for practical applicability. However, there are some questions which should be addressed by the authors to strengthen their work and clarify both their procedures and conclusions. These questions and suggestions are grouped below:

We thank the reviewer for the helpful discussion and comments. We have addressed the comments and revised the manuscript accordingly.

1. Site blocking:
What are the authors' conclusions or next hypotheses based on the competition effect that they observe? I'm particularly interested in distinguishing the "competing reaction" versus the "site-blocking" depiction, which is shown in figure 1A. Clearly the reactions are occurring concurrently, which is what the first panel shows. But based on Figure 2, there is no distinct change in cathodic operating potential as NO is added to the feed stream. A decrease in CO2R FE at constant current means the CO2 partial current has decreased. This happening while potential is held more or less constant would imply competition for active sites, or "site blocking," not necessarily brought about by catalyst poisoning but by high coverage of active sites which are dynamically exchanging substrates. What do the authors think about this? Do they have an alternative explanation for why partial current for CO2R would decrease at a constant potential? (Note: if potential is not roughly constant, then the potential axis on Figure 2 needs to be re-scaled to accurately depict changes.)

Reply:

Thank you for the fruitful comment. We actually observe a shift in cathodic potential when NO is introduced during CO_2RR. We have conducted the experiment more carefully and adjusted the scale of the potential axis to show this more clearly (**Fig. R8**). Although the change in potential is unclear for Ag catalyst, due to the large fluctuation in potential caused by the continuous bubble formation and flush out at the electrode surface, CV

measurements with CO_2 and 0.83% NO clearly show the shift in potential for all three catalysts (**Fig. S3**). Even though we do not exclude the possibility of "site-blocking," brought by high coverage of active sites by NO, NORR intermediates, and NORR products, our hypothesis that the main effect of NO_x during CO_2RR is "competing reaction" still holds. The main effect of NO_x is reducing CO_2RR partial current densities from the preferential reduction of NO_x, which are thermodynamically more favorable.

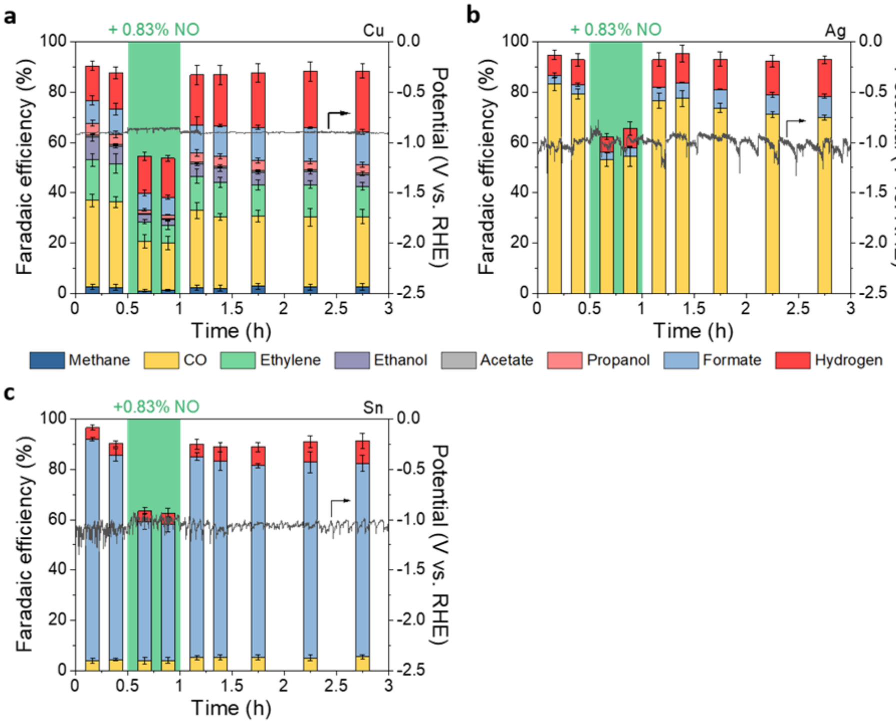

Figure R8. (New Figure 2a-c). CO_2 electroreduction performance in the presence of NO. Faradaic efficiency and applied potential vs. time on (a) Cu, (b) Ag, and (c) Sn catalysts at a constant current density of 100 mA cm^{-2} in 1 M $KHCO_3$ for 3 h. Gas feed was 83.3% CO_2 and 16.7% Ar, and 83.3% CO_2, 15.87% Ar, and 0.83% NO (green). 0.83% NO was introduced at 0.5 h for 0.5 h. Corresponding Faradaic efficiencies are provided in Table S2-4.

In the revised manuscript, we have adjusted the scale of potential axis to show the potential profiles more clearly.

2. Transport:
a. The argument that N2O is less reactive than NO2 or NO is at least partially predicated on the notion that all of these reactions are under kinetic control at the conditions of interest – is this the case? I drew up some math for a flooded system that wouldn't apply to a GDE, but I'll present it here anyway: NO as an example is 18x less soluble than CO2 with concentration in water of around 2 mM at 25 C saturation. So 1% NO gas would result in a 0.02 mM NO solution. In flooded configuration one would not expect higher rates than hundreds of micro-Amps at most. By what factor can we expect transport-limited currents to increase in the GDE configuration, especially given the catalyst-flooding argument that the authors present?

Reply:

The effectiveness of the three-compartment flow cell configuration regarding gas transport has been demonstrated in our previous work on the electrochemical reduction of CO (CORR).[1] The solubility of CO in water at 25°C (~1mM) is even lower than NO, which has limited the CORR current densities to less than 0.5 mA cm^{-2} on Cu catalyst in a conventional batch cell.[2] In contrast, >800 mA cm^{-2} of CORR current density, which is three orders of magnitude greater than that obtained from conventional batch cells, was achieved in the three-compartment flow cell.[1] Similar enhancement in the mass transport of NO is expected in the three-compartment flow cell.

References

1. Jouny, M., Luc, W. & Jiao, F. High-rate electroreduction of carbon monoxide to multi-carbon products. *Nat. Catal.* **1**, (2018).
2. Li, C. W., Ciston, J. & Kanan, M. W. Electroreduction of carbon monoxide to liquid fuel on oxide-derived nanocrystalline copper. *Nature* **508**, 504–507 (2014).

b. The above comment could be addressed experimentally fairly easily. A good place to start is with cyclic voltammetry. Fig. S3 is interesting – I'd be curious to see what the CV would look like with 1% NO in an inert balance (N2 or Ar). One would expect with the large difference in standard reduction potential between NO and CO2 that the onset of NOR might be significantly positive of CO2R. It's hard to tell from these plots what the NOR looks like on its own, even if we just assume that the currents are purely additive. Is it possible that even in this GDE setup, because NO is so dilute, the reaction could be reaching transport limitations prior to CO2R onset? Especially on Ag it appears that NOR sort of "plateaus" at around -0.7 V vs. RHE at a current density of ~10 mA/cm2. It's hard to tell without the NO-only CV.

Reply:

We have conducted CV measurements with different concentrations of NO_x in Ar (without CO_2) on all three catalysts to better understand the onset potentials and investigate the transport of NO_x (**Fig. R10-12**). More positive onset potentials of NO_x electroreduction (NO_xRR) compared to CO_2RR have been confirmed, except for N_2ORR on Sn catalyst (**Fig. R12c**), in which Sn appears to be inactive for N_2ORR. The results also suggest that all NO_xRR, except for N_2ORR on Sn catalyst, are mass transport limited at 0.83% since the CV measurement shifts with increasing concentrations of NO_x. Because NO_xRR is mass transport limited at 0.83%, we no longer claim that N_2O is less reactive than NO and NO_2. Alternatively, we observed that the main products of NORR are NH_3 and NH_2OH, which require 5 and 3 electrons, respectively, whereas the main product of N_2RR is N_2, which only requires 2 electrons. Given that all NO_x readily react on the catalyst surface, the reduction of NO and NO_2 is expected to consume more electrons than that of N_2O, and thus greater losses in FE, due to the greater number of electrons involved in the NO_xRR.

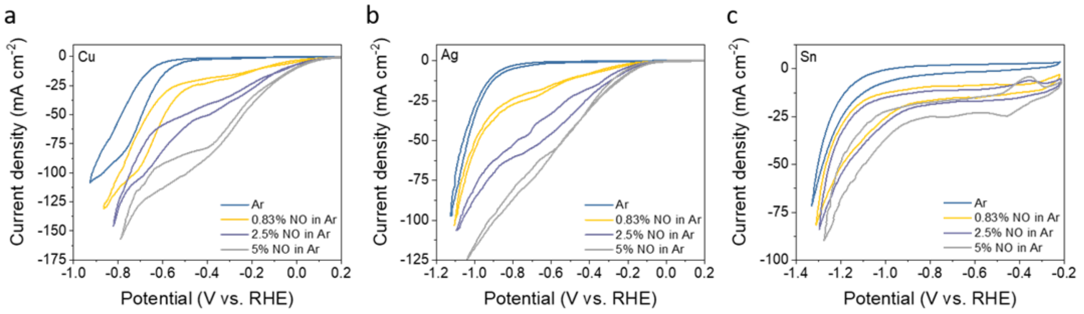

Figure R10 (New Figure S4). Cyclic voltammograms on (a) Cu, (b) Ag, and (c) Sn catalysts in 1M $KHCO_3$ under different concentrations of NO in Ar. Scan rate: 50 mV s^{-1}. Shifts in CV measurements suggest that NORR is mass transport limited.

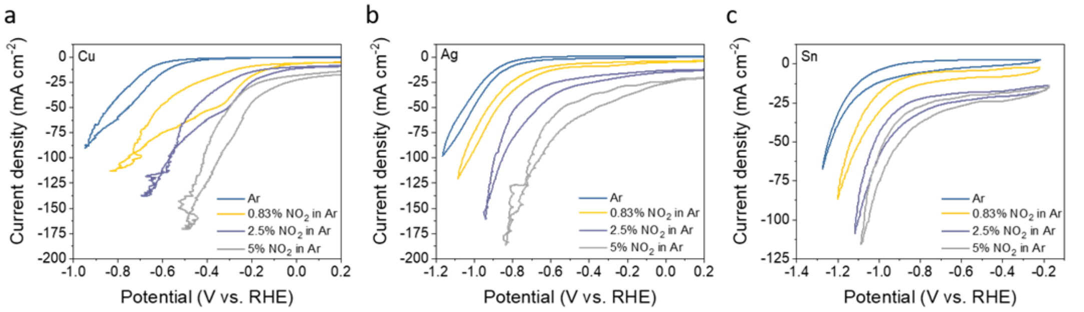

Figure R11 (New Figure S9). Cyclic voltammograms on (a) Cu, (b) Ag, and (c) Sn catalysts in 1M $KHCO_3$ under different concentrations of NO_2 in Ar. Scan rate: 50 mV s^{-1}. Shifts in CV measurements suggest that NO_2RR is mass transport limited.

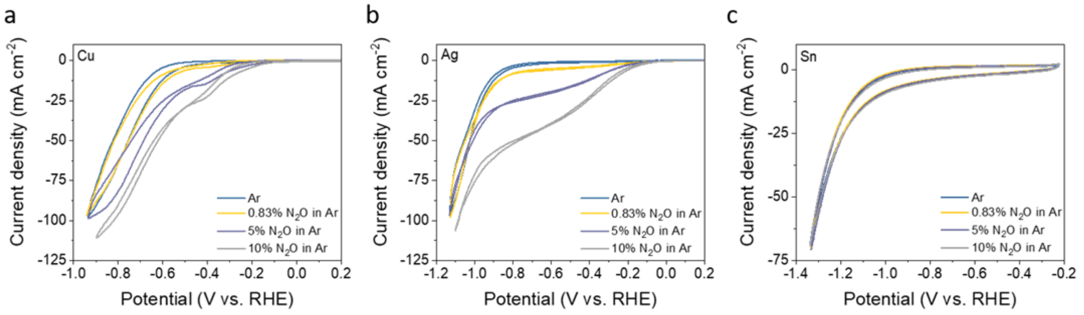

Figure R12 (New Figure S11). Cyclic voltammograms on (a) Cu, (b) Ag, and (c) Sn catalysts in 1M $KHCO_3$ under different concentrations of N_2O in Ar. Scan rate: 50 mV s^{-1}. Shifts in CV measurements on Cu and Ag catalysts suggest that NO_2RR is mass transport limited. N_2O had negligible effect on Sn catalyst.

In the revised manuscript, we have added these figures and revised the discussion. The following sentences are now included in the revised manuscript:

> "NO and NO_2 impurities have more severe impacts on CO_2RR FE than N_2O, likely due to the greater number of electrons required in the NO_x reactions." (Page 3, Lines 71-72)

> "CV measurements under different concentrations of NO in Ar also confirmed more positive onset potentials of NORR than CO_2RR and showed that NORR at 0.83% NO is mass transport limited (Fig. S4)." (Page 5, Lines 124-126)

"NO and NO_2 show greater losses in FE than N_2O on all three catalysts, likely due to the greater number of electrons required in the reactions. As will be discussed in the following section, the main products of NORR are NH_3 and NH_2OH, which require 5 and 3 electrons, respectively, while the main product of N_2ORR is N_2. Given that all NO_x readily reacts at the catalyst surface, the same amount of NO and NO_2 consume more electrons than N_2O, causing greater losses in CO_2RR FE." (Page 6, Lines 168-173)

"The impact of NO and NO_2 is more severe than that of N_2O in CO_2RR due to the greater number electrons involved in NORR and NO_2RR compared to N_2ORR." (Page 10, Lines 303-305)

3. Gas composition choices & procedures:
a. Methods: Flow Cell Electrolysis – this section says that stock gases were already dilute: NO stock was 5% in Ar and NO2 was 1% in Ar. This means that a large portion of the inlet gas consisted of Ar. Can the authors speak to how they kept the partial pressure of CO2 constant in the electrolysis cell across all conditions? This is important, because unless this constant-CO2-pressure condition was met, the decrease in CO2R FE could be attributed merely to lower CO2 partial pressures rather than to the interference of NOx as the authors claim. (Note that constant mass flow does not equate to constant pressure!)

Reply:

Please see our response to **Q2 from Reviewer 2**. In short, to eliminate the effect of the change in CO_2 partial pressure during the introduction of NO_x mixture containing Ar (e.g., 5% NO/Ar and 5% NO_2/Ar), CO_2 partial pressure was maintained at 0.833 bar throughout the experiment by flowing Ar with CO_2 (83.3% CO_2 and 16.7% Ar) when NO_x was not required in the gas feed.

b. I'm intrigued by the authors' choice to use 0.3% NO2 while the other gases are present at 1%. Why the distinction? It seems that even if the percentage NOx component used is indicative of its concentration in industrial flue gas, we would gain more by comparing the gases in equal quantity to normalize for gas availability. The authors should justify their decision.

Reply:

We are sorry for the confusion. 0.3% NO_2 was chosen because of the gas availability at the time of the experiment (1% NO_2/Ar). In the revised manuscript, we kept all NO_x studies with the same composition, 83.3% CO_2, 15.87% Ar, and 0.83% NO_x, for a fair comparison. The influence of 0.83% NO_2 in CO_2RR at constant current density of 100 mA cm^{-2} is shown in **Fig. R13.** The losses in CO_2RR Faradaic efficiency caused by 0.83% NO_2 is compared with those of NO and N_2O in **Fig. R14**.

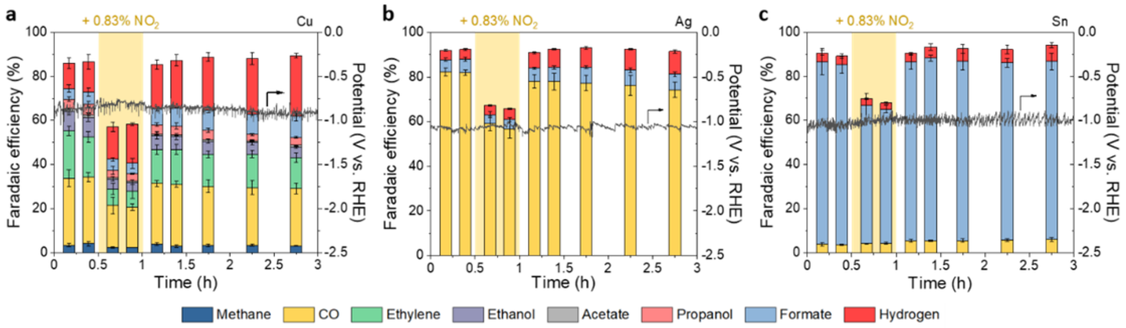

Figure R13 (New Figure S6). Faradaic efficiency and applied potential vs. time on (a) Cu, (b) Ag, and (c) Sn catalysts at a constant current density of 100 mA cm^{-2} in 1 M $KHCO_3$ for 3 h. Gas feed was 83.3% CO_2 and 16.7% Ar, and 83.3% CO_2, 15.87% Ar, and 0.83% NO_2 (yellow). 0.83% NO_2 was introduced at 0.5 h for 0.5 h. Corresponding Faradaic efficiencies are provided in Tables S7-9. Error bars represent the standard deviation of three independent measurements. Faradaic efficiency decreases with the introduction of NO_2 on all three catalysts.

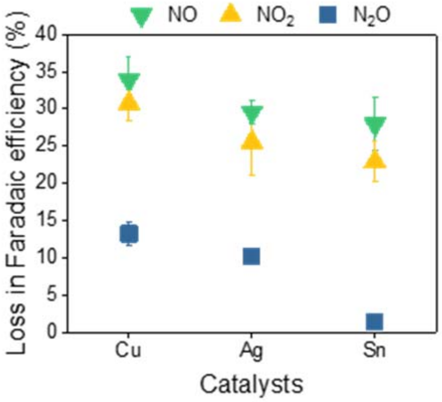

Figure R14 (New Figure 3c). Loss in Faradaic efficiency during CO_2 electroreduction due to 0.83% NO, 0.83% NO_2, and 0.83% N_2O on Cu, Ag, and Sn catalysts. Error bars represent the standard deviation of three independent measurements.

We have also obtained XPS measurements **(Fig. R15 and 16)** and SEM images **(Fig. R17)** with 0.83% NO_2, in which the results were similar to those obtained with 0.23% NO_2. Unfortunately, new XAS results with 0.83% NO_2 could not be obtained since the equipment was not accessible due to the COVID-19 pandemic. Although we present the XAS results with 0.23% NO_2 instead of 0.83% NO_2, insight on the effect of the presence of NO_2 during CO_2RR on the catalyst oxidation state is still obtained.

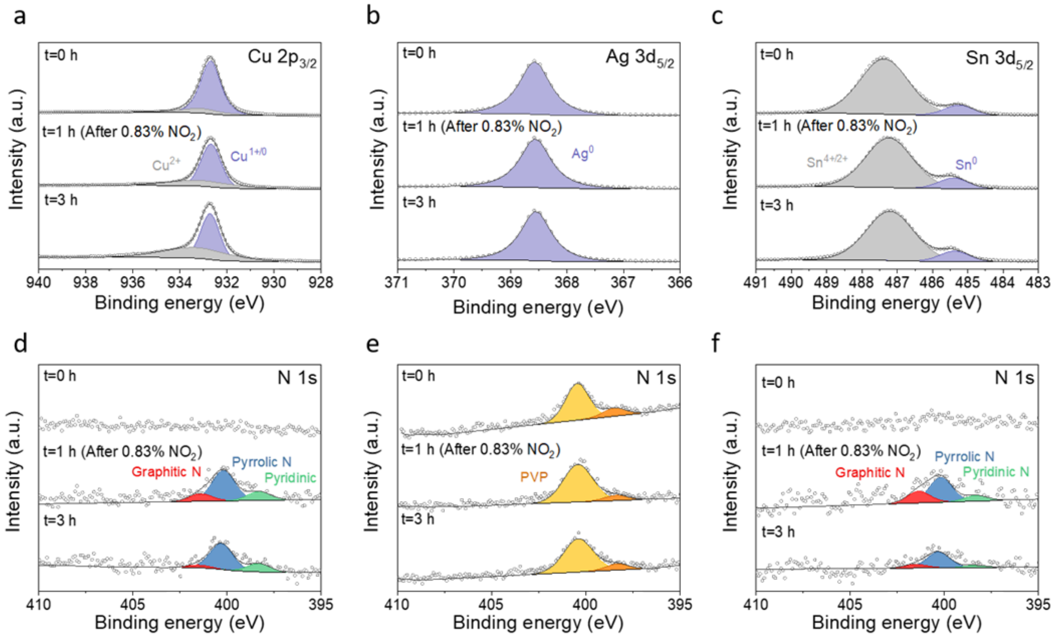

Figure R15 (New Figure S28). XPS measurements of (a) Cu, (b) Ag, and (c) Sn electrodes before exposure to 0.83% NO_2 (t=0 h), after exposure to 0.83% NO_2 (t=1 h), and after 3 h electrolysis (t=3 h) from a 100 mA cm^{-2} constant current CO_2RR experiment with the introduction of 0.83% NO_2. Corresponding N 1s XPS measurements of (d) Cu, (e) Ag, and (f) Sn electrodes. Incorporation of N into GDL is observed on Cu and Sn electrodes. In the case of Ag electrode, the investigation of N incorporation was limited due to the presence of PVP surfactant. Corresponding details are provided in Table S13.

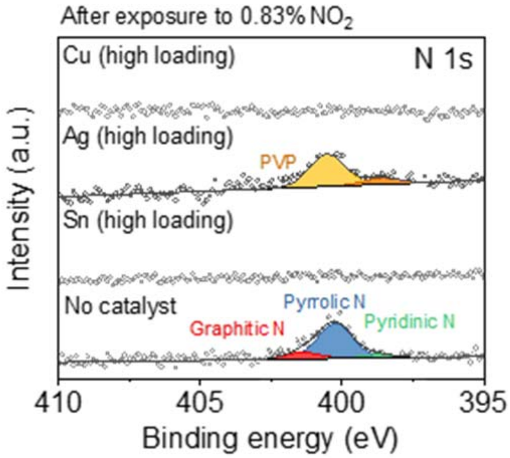

Figure R16 (New Figure S30b). XPS measurements of electrodes with high catalyst loadings of 2.0 mg cm^{-2} Cu, Ag, and Sn, and no catalyst at t=1 h after exposure to (a) 0.83% NO, (b) 0.83% NO_2, and (c) 0.83% N_2O for 0.5 h. The results confirm that N is incorporated in GDL rather than metal catalysts.

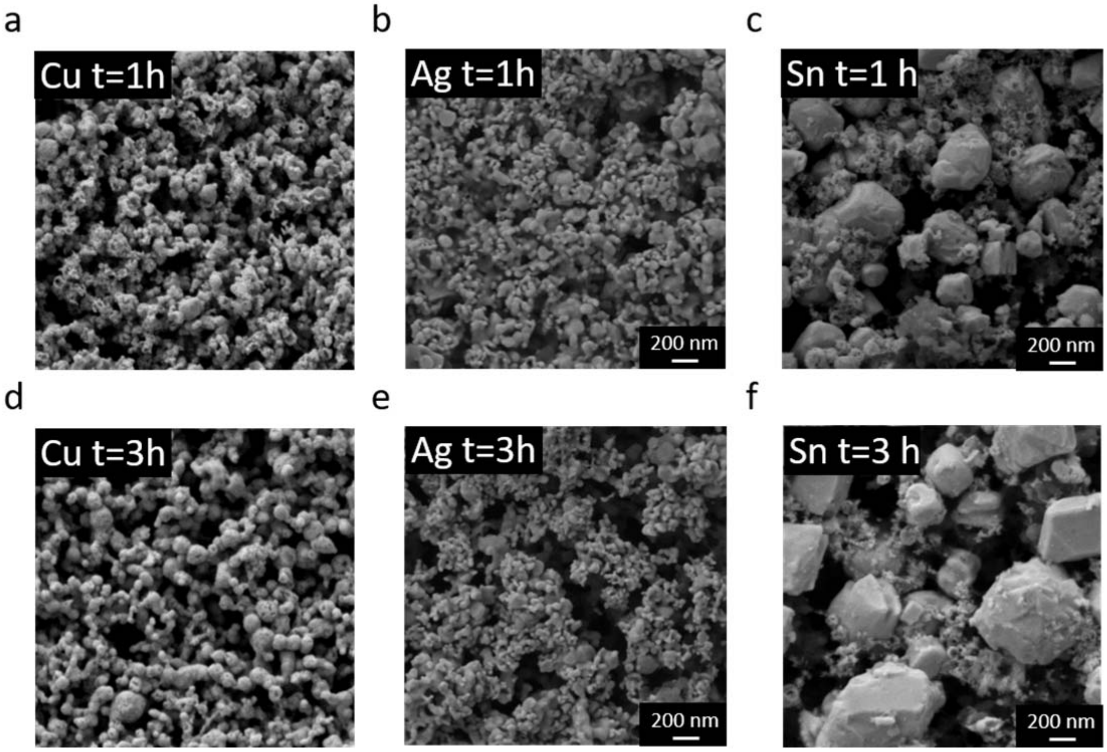

Figure R17 (New Figure S34b and e, S35b and e, and S36b and e). SEM images of Cu electrodes obtained at (a) t=1 h and (d) t=3 h, Ag electrodes obtained at (b) t=1 h and (e) t=3 h, and Sn electrodes obtained at (c) t=1 h and (f) t=3 h after exposure to 0.83% NO_2.

In the revised manuscript, we have replaced all NO_2 data with 0.83% NO_2 except for the XAS results. We have specifically stated that the XAS experiment was conducted with 0.23% NO_2 and added the following explanation in the figure caption **(Fig. S32)**: "Although 0.23% was used instead of 0.83% due to the availability of the gas at the time of the experiment, insight on the effect of the presence of NO_2 during CO_2RR on the catalyst oxidation state is still obtained."

c. In all of the cases where FEMS was used, the authors have justified their use of 1% NO in Ar rather than in CO2. It appears that the total current density applied during these tests was the same as that applied in the experiments with 1% NO in CO2. It would seem that the potential required in order to achieve this current would be greater in the case of 99% inert gas than it would be in the case of fully reactive gas. What is the cathode potential during these tests without CO2? If the cathode has to be held at a higher potential in order to achieve the same current density, then is not the NOR facing a different overpotential? How then are the results in the presence of 99% Ar translatable to a system with 99% CO2, when the catalytic system used to interrogate CO2 reduction response to NOx and the one used to interrogate NOx reduction products both contain different gases and are held at different potentials?

Reply:

We agree with the reviewer that the operating potentials to achieve 100 mA cm^{-2} for 0.83% NO in Ar are different from those of 0.83% NO in CO_2, and it would be more appropriate to conduct the FEMS experiment at the same potentials. Therefore, we have conducted the

FEMS experiment at constant potentials at -0.90, -1.00, and -1.05 V vs. RHE on Cu, Ag, and Sn catalysts, respectively. (**Fig. R18-20**) As suggested later in **Q8e from reviewer 3**, m/z=28 gives more direct evidence of the formation of N_2, so we now present m/z=28 instead of m/z=14 to show the production of N_2. We have also switched to using m/z=12 from m/z=22 to obtain the background coming from CO_2 in the electrolyte, since m/z=12 gives twice as greater signal of CO_2 than m/z=22. We observed the production of H_2 (m/z=2), NH_3 (m/z=17), N_2 (m/z=28), and N_2O (m/z=44) and the consumption of NO (m/z=30) on Cu and Ag catalysts. In contrast, the production of NH_3 was not observed on Sn, likely due to the extremely small amount of NH_3 produced. FEMS results are consistent with the results obtained from spectrophotometry and GC analysis, and the production of N_2O, which was difficult to measure via GC, was clearly observed in FEMS.

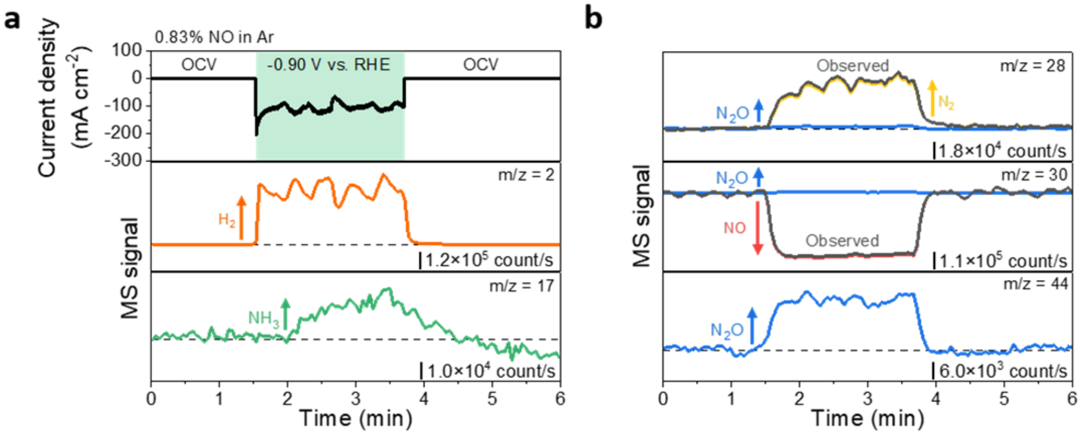

Figure R18 (New Figure 4c and d). (a) Measured current density vs. time, and deconvoluted MS signal vs. time for m/z=2, m/z=17, (b) m/z=28, m/z=30, and m/z=44 from FEMS on Cu catalyst in 1M $KHCO_3$ with 0.83% NO in Ar. -0.90 V vs. RHE was applied for approximately 2 minutes starting at t=1.5 min. NORR products have been deconvoluted using the mass spectra of individual products shown in Fig. S11. Additional information is provided in the methods section and Fig. S18 and 19.

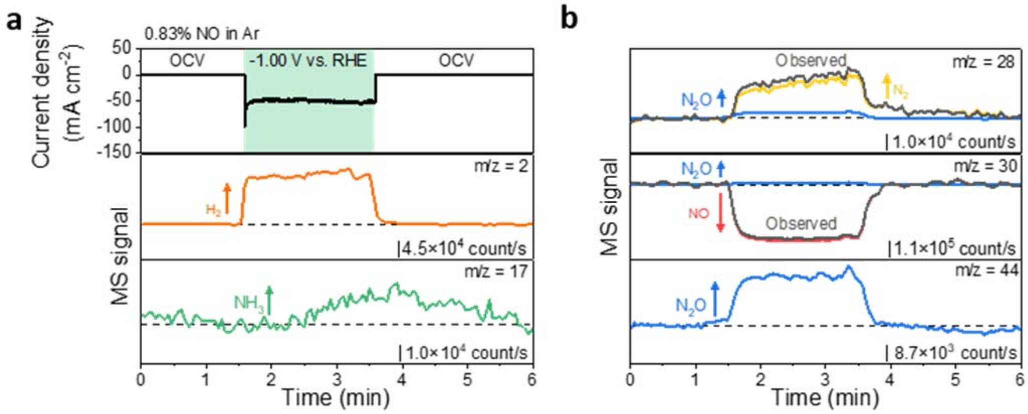

Figure R19 (New Figure S22). (a) Measured current density vs. time, and deconvoluted MS signal vs. time for m/z=2, m/z=17, (b) m/z=28, m/z=30, and m/z=44 from FEMS on Ag catalyst in 1M $KHCO_3$ with 0.83% NO in Ar. -1.00 V vs. RHE was applied for approximately 2 minutes starting at t=1.5 min. NORR products have been deconvoluted using the mass spectra of individual products shown in Fig. S11. Additional information is provided in the

methods section and Fig. S18 and 19.

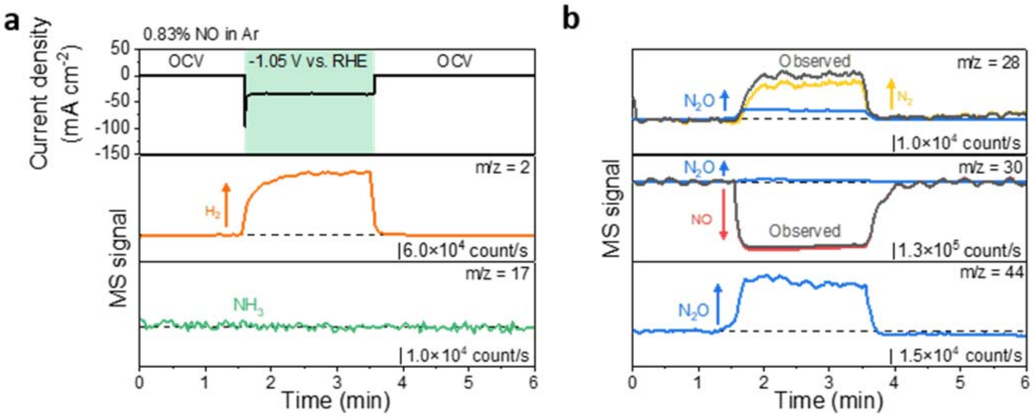

Figure R20 (New Figure S25). (a) Measured current density vs. time, and deconvoluted MS signal vs. time for m/z=2, m/z=17, (b) m/z=28, m/z=30, and m/z=44 from FEMS on Sn catalyst in 1M $KHCO_3$ with 0.83% NO in Ar. -1.05 V vs. RHE was applied for approximately 2 minutes starting at t=1.5 min. NORR products have been deconvoluted using the mass spectra of individual products shown in Fig. S11. Additional information is provided in the methods section and Fig. S21 and 22.

In the revised manuscript, we include the new FEMS data conducted at constant potential and the following discussion:

> "When a constant potential of -0.90 V vs. RHE was applied at t=1.5 for approximately 2 minutes, MS signals of NO (m/z=30) decreased while those of H_2 (m/z=2), NH_3 (m/z=17), N_2 (m/z=28) and N_2O (m/z=44) increased (Fig. 4c and d), indicating the consumption of NO and the formation of H_2, NH_3, N_2, and N_2O. The formation of NH_3 and N_2 detected by FEMS is in agreement with the results obtained from spectrophotometry and GC analysis, respectively. The production of N_2O, which was difficult to measure via GC, was clearly observed in FEMS, suggesting that N_2O is also one of the NORR products. NH_2OH was not detected in FEMS, because it is nonvolatile.[33] Similarly, FEMS results also suggest the formation of N_2 and N_2O on Ag and Sn catalysts (Figs. S20-25). However, the formation of NH_3 was observed only on Ag and not on Sn, likely due to the small amount of NH_3 produced on Sn. Collectively, NH_3, NH_2OH, N_2, and N_2O have been determined as the NORR products." (Page 8, Lines 221-231)

d. NO2 hydrolyzes in water to generate nitric acid and nitrous acid. To what extent does the gas dissolve into the electrolyte? Could this affect the catalyst? Is this effect dampened by the fact that the electrolyte is constantly replenished?

Reply:

We have measured the pH at the outlet of the electrolyzer at different time points (i.e., before, during, and after NO_x introduction during CO_2RR) (**Fig. R21**). The introduction of NO and N_2O had negligible effect on the pH, while NO_2 reduced the pH slightly by 0.03. Although NO_2 hydrolyzes to generate nitric acid and nitrous acid, the effect of NO_2 in pH was very small, possibly due to the low amount of NO_2 in the gas feed, rapid reaction of NO_2 at the catalyst surface, which prevents NO_2 from penetrating to the bulk electrolyte, and flowing electrolyte which is constantly replenished.

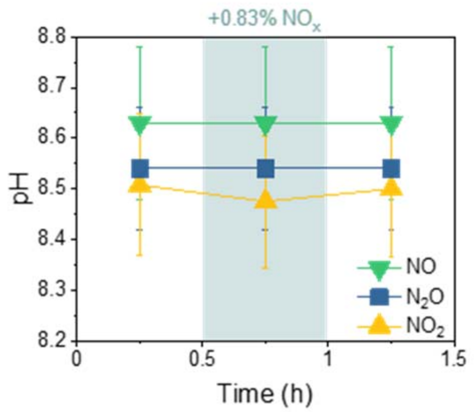

Figure R21 (New Figure S12). pH measured at the outlet of the electrolyzer from a constant 100-mA cm^{-2} CO_2RR experiment with the introduction of various NO_x. pH of 1M $KHCO_3$ before entering the electrolyzer was 7.8$\pm$0.1. Error bars represent the standard deviation of independent measurements from Cu, Ag, and Sn catalysts. The presence of NO and N_2O has negligible effect in pH, while the presence of NO_2 slightly decreases the pH by 0.03.

In the revised manuscript, we have included the discussion on the effect of NO_x in pH as follows:

> "Furthermore, pH was measured at the outlet of the electrolyzer at different time points (i.e., before, during, and after NO_x introduction) to investigate the effect of NO_x on the electrolyte pH (Fig. S12). The measured pH shows that NO and N_2O have negligible effect on the pH, while NO_2 slightly decreases the pH by 0.03. Although NO_2 hydrolyzes to produce nitric acid and nitrous acid,[26] the effect in pH is very small, possibly due to the low amount of NO_2 in the gas feed, rapid reaction of NO_2 at the catalyst surface which prevents NO_2 from penetrating to the bulk electrolyte, and flowing electrolyte which is constantly replenished." (Page 5, Lines 179-186)

4. N2O as a less-reactive species:
a. Line 133: the authors claim that CO2R on Sn catalyst is not affected by the presence of N2O because Sn is a bad N2O catalyst, then provide a citation. However, in the citation it appears that at -1.7 V vs. Ag/AgCl (a potential within the range of CVs in the present work's SI), Sn catalyzes N2O reduction to N2 with ~70% FE at a total current of 8 mA/cm2, and this was in a flooded electrode configuration, although at 100% N2O. Is the explanation that the current toward N2O reduction is small in comparison to the 100 mA/cm2 current being drawn by CO2 reduction? Could concurrent CO2 reduction actually diminish the amount of N2O reduction occurring? It would be nice if the authors could give their thoughts.

Reply:

In the provided reference, Sn requires much greater overpotential (~ 1 V) to achieve 8 mA cm^{-2} of N_2ORR compared to Cu and Ag, suggesting that Sn is less effective in N_2ORR than Cu and Ag. We have performed CV measurements with different concentrations of N_2O in Ar, in the absence of $CO_{2,}$ on all three catalysts (**Fig. R12**). We observed that the CV measurement on Sn catalyst did not change even when 10% N_2O was introduced, whereas clear shifts were observed on Cu and Ag catalysts. Therefore, the concurrent CO_2RR is unlikely responsible for the low activity of N_2ORR on the Sn catalyst.

b. Lines 143-145 suggest that Cu is a good NOx reduction catalyst and point to this as a direction for future study. It is worth pointing out that citation 23 mentioned above does actually include Cu as one of the best-performing N2O reduction catalysts, achieving high FEs for N2O to N2 at relatively low overpotentials.

Reply:

We thank the reviewer to point it out. In the revised manuscript, we have added the following discussion:

> "Indeed, Cu has been demonstrated as one of the more active metals for the electroreduction of NO[27] and N_2O,[25] in which Cu achieved high FE in N_2ORR to N_2 at relatively low overpotentials." (Page 6, Lines 174-176)

5. Catalyst oxidation state:
It seems that the factors of oxidation by NOx and reduction by potential are competing here. The authors have shown that pre-treated catalysts will revert back to metal under reductive potential, but this does not necessarily mean that this is the case even when the catalyst is being actively exposed to NOx. It seems that in-operando experiments would be most useful here to definitively show the oxidation state of the catalyst. However, lacking sure evidence, I would tend to form a hypothesis in agreement with the authors' conclusions – it would not surprise me if under the strongly reductive potentials required for CO2R, the catalysts were fully metallic.

Reply:

We agree that our XAS results do not provide information on the oxidation state of Cu catalysts when the catalysts are actively exposed to NOx. Instead, our results show that Cu catalyst is fully metallic under reaction conditions after the catalyst has been exposed to NOx and NOx has been removed from the CO_2 stream.

In the revised manuscript, we have clarified this point with the following discussion:

> "XAS measurements on Cu samples exposed to NO_2 and N_2O also exhibited similar behaviors as the NO-treated Cu sample (Figs. S32 and 33), confirming that the Cu catalyst remains or revert to fully metallic under reaction conditions after NO_x is removed from the CO_2 stream. " (Page 10, Lines 286-289)

6. FE closure:
Can the authors explain why their FE closure is sub-90% in all cases (and sub-70% in some) even with a pure CO2 feed? At such high currents there should be little issue detecting products. Can the balance be attributed to product crossover to the anolyte?
Reply:

We apologize for the confusion. The total FE of sub-70% was due to the unmeasured H_2 FE for NO_2 experiments (Please see our response to **Q3 from Reviewer 1**). In the revision, we have measured H_2 FE and the total FE is above 85%. We have observed that <3%, <2%, and 5-10% of formate FE are detected from the anode side on Cu, Ag, and Sn catalysts, respectively. Because formate crossover was substantial on Sn catalyst, all anolyte has been analyzed for and included in the Sn formate FE. This has been stated in **Tables S2-4 and 7-12**. Other missing FE may be attributed to volatile liquid products exiting with the gas stream and/or gas products exiting with the liquid electrolyte stream.

7. Transient selectivity changes:

The authors claim that increases in HER (and if I may add from my own interpretation, formate – which tends to be an “outer-sphere” CO2R product) over time are due to GDE flooding, based on a previous paper of theirs. Do we know in this instance whether this holds true? For instance, what is the 3-hour CO2R baseline (no NO added)? Could transient changes (e.g. increase in HER FE) be attributed to metal plating? This is not a huge priority, since transient changes are not on a scale that derails the central argument of the paper.

Reply:

We have obtained 3-hour CO_2RR baseline on all three catalysts (**Fig. R3**). H_2 FE increases over time, and we also observed an increase in the formate FE on Cu and Ag, while the formate FE decreases on Sn. Therefore, the introduction of NOx is unlikely the cause of the transient changes in product selectivity.

8. General comments on methods and SI:

a. Could the authors provide some supporting data or at least a comprehensive reference on their Nessler’s method calibration curves, exact procedure, etc.?

Reply:

We have observed that Nessler’s method is unreliable for the quantification of ammonia in the presence of hydroxylamine (NH_2OH). We have use indophenol blue method instead to quantify ammonia, and this will be discussed further in **Q8g from Reviewer 3**.

b. The choice to detect NOR products via FEMS is interesting. It seems that the drawback mentioned by the authors in the SI is significant: simultaneous NOR and CO2R are difficult to study. Is it not possible to use in-line gas chromatography to detect N2 and N2O, as was done during N2O reduction?

Reply:

We have conducted GC analysis of the gas products from CO_2RR in the presence of 0.83% NO and detected a substantial amount of N_2 (**Fig. R5**). However, the concentration of N_2O was below the detection limit of GC, in which the detection limit corresponded to approximately 2% N_2O FE in NORR. Therefore, a more sensitive FEMS was required to determine the formation of N_2O. The production of N_2O, which was difficult to measure via GC, was clearly observed in FEMS, suggesting that N_2O is also one of NORR products.

In the revised manuscript, we have included the following sentences on the use of GC to quantify N_2 and N_2O:

> “… and N_2 was detected via GC (Fig. S15). We note that the concentration of N_2O in the gas product stream was below the detection limit of GC, suggesting that N_2O FE was below 2% FE on all three catalysts. “(Page 6, Lines 191-193)

c. Could the authors provide a more thorough SI explanation of the MS processing techniques used? It is currently a bit difficult to follow the logic behind these steps. After reading for quite some time I eventually ascertained that, for example, Figure S9 represents the raw data for NOR on Cu; Figure S10 shows how background contributions from water and CO2 are subtracted from the m/z = 17 and 44 peaks respectively; Figure 4c shows the background-subtracted data for these two m/z and additionally m/z = 30 which contains both NO and N2O, but which can be distinguished by accounting for the N2O area based off the m/z = 44 peak as well as the ratio of peaks in the pure N2O spectrum; and Figure 4d combines all of this information to show how the m/z = 14 peak can be broken down into

contributions from NO consumed, NH3 produced, and N2O produced. A description to this end would be useful.

Reply:

The interpretation of the reviewer is correct, and we apologize for the confusion. As mentioned previously in our response to **Q3c from Reviewer 3**, because m/z=28 gives more direct evidence of the production of N_2 and N_2O, we now present m/z=28 instead of m/z=14.

In the revised manuscript, we have provided detailed explanation on the deconvolution procedure in the methods section.

> "For the deconvolution of m/z=17 signal, m/z=17 signal from water was first determined using m/z=18 signal. Next, the contribution from water to m/z=17 signal was subtracted from the observed m/z=17 signal to obtain the signal from ammonia. For the deconvolution of m/z=28 and 44 signals, m/z=28 and 44 signals from CO_2 in the electrolyte was first determined using m/z=12 signal. m/z=12 signal was smoothed using the Savitzsky-Golay method with a window of 30 data points to reduce the oscillations in the signal prior to deconvolution. Next, the contributions from CO_2 to m/z=28 and 44 were subtracted from the observed m/z=28 and 44 signals to obtain the signals from NORR products. m/z=44 signal corresponded to the signal from N_2O, and this was used to calculate the contribution of N_2O to m/z=28 and 30. Lastly, the contributions of N_2O to m/z=28 and 30 were subtracted from the m/z=28 and 30 signals from NORR products, respectively, to yield N_2 and NO signals, respectively. All deconvolution was conducted using MATLAB. Mass spectra of NH_4OH, NO, N_2O, N_2, H_2O, and CO_2 used for the deconvolution were obtained using the same MS equipment (Fig. S17)." (Page 13, Lines 404-417)

d. The authors should provide a sample MS of CO2 in Figure S11 if they're going to be using the CO2 spectrum to process data.

Reply:

In the revised manuscript, we have provided a sample MS of H_2O and CO_2 (**Fig. R22**), which were used for the deconvolution.

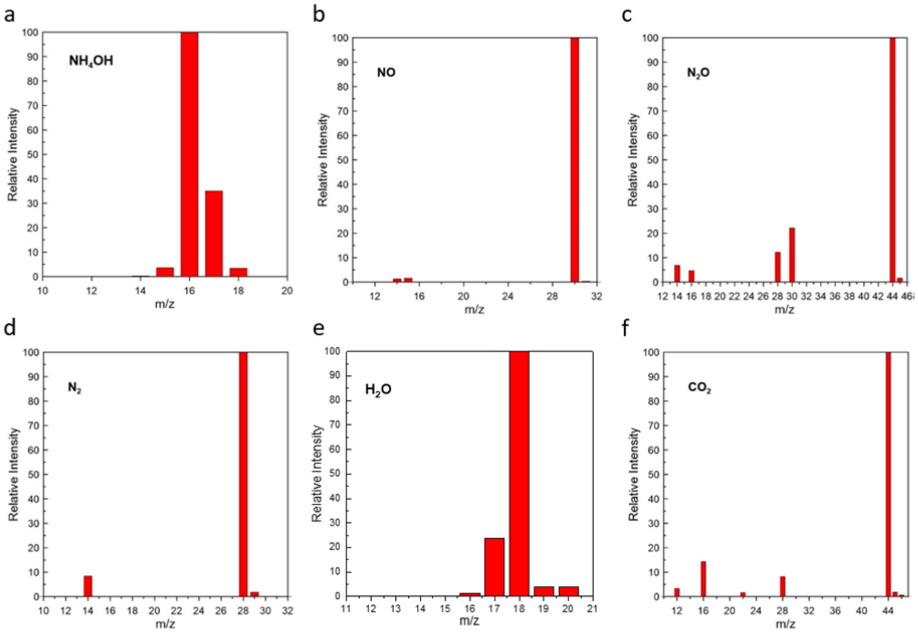

Figure R22 (New Figure S17). Mass spectra of (a) NH_4OH, (b) NO, (c) N_2O, (d) N_2, (e) H_2O, and (f) CO_2. MS signals were deconvoluted using the following mass spectra.

e. The authors argue that the residual of the m/z = 14 peak being near-zero is sufficient to say that no N2 is produced. While I agree this should be decently good evidence for that, could we not tell this more directly (i.e. with fewer contributions to subtract out) by examining the m/z = 28 peak, which only appears to contain contributions from N2 and N2O? Are results obtained in this manner consistent with results obtained using the authors' approach?

Reply:

We agree that m/z=28 signal gives more direct evidence about the formation of N_2 and N_2O, and we now present m/z=28 signal data (**Fig. R18-20**). Analysis of the m/z=28 signal shows that N_2 is produced during NORR, although previous analysis on m/z=14 signal showed that N_2 is not produced. Because m/z=28 signal is more reliable than m/z=14 and N_2 is also detected in GC analysis, we conclude that N_2 is one of the NORR products on Cu, Ag, and Sn catalysts.

f. Can the authors detail the procedure for XPS and XAS preparation? Were the catalysts exposed to air in the process of transferring from electrolysis cell to XPS for analysis? If so, how long did this take? Similarly, how long before XAS were the catalysts exposed to NOx? In what environment were they kept in the interim?

Reply:

We have added more details for the XPS and XAS experiments. Please see our response to **Q7 from reviewer 1**.

g. Nessler's reagent is prone to significant errors in quantification and the indophenol method should be used instead to confirm results

Reply:

We have observed substantial interference from hydroxylamine (NH_2OH) in Nessler's method, making Nessler's method unreliable for the quantification of ammonia when NH_2OH is present in the solution (**Fig. R23**). Instead, we have used indophenol blue method,[1] which is more reliable, to quantify NH_4OH (**Fig. R24**). We have confirmed that NH_2OH does not show significant absorbance in the indophenol blue method. After noticing the possibility of the production of NH_2OH during NORR, we have also quantified NH_2OH using the procedure modified from the method reported by Afkhami et al (**Fig. R25**).[2] We have confirmed that NH_4OH does not show significant absorbance in this method. The newly measured NORR products are shown in **Fig. R26**. NH_3 substantially decreased compared to the previously reported values based on Nessler's method, and considerable amount of NH_2OH was quantified on Ag and Sn catalysts. In fact, NH_2OH has also been shown to be the main NORR products.[3] Overall, we observed that NH_3, NH_2OH, N_2, and N_2O are the main NORR products, in which the product selectivity varied among different catalysts.

References

1. Andersen, S. Z. *et al.* A rigorous electrochemical ammonia synthesis protocol with quantitative isotope measurements. *Nature* **570**, 504–508 (2019).

2. Afkhami, A., Madrakian, T. & Maleki, A. Indirect kinetic spectrophotometric determination of hydroxylamine based on its reaction with iodate. *Anal. Sci.* **22**, 329–331 (2006).

3. Rosca, V., Duca, M., DeGroot, M. T. & Koper, M. T. M. Nitrogen Cycle Electrocatalysis. *Chem. Rev.* **109**, 2209–2244 (2009).

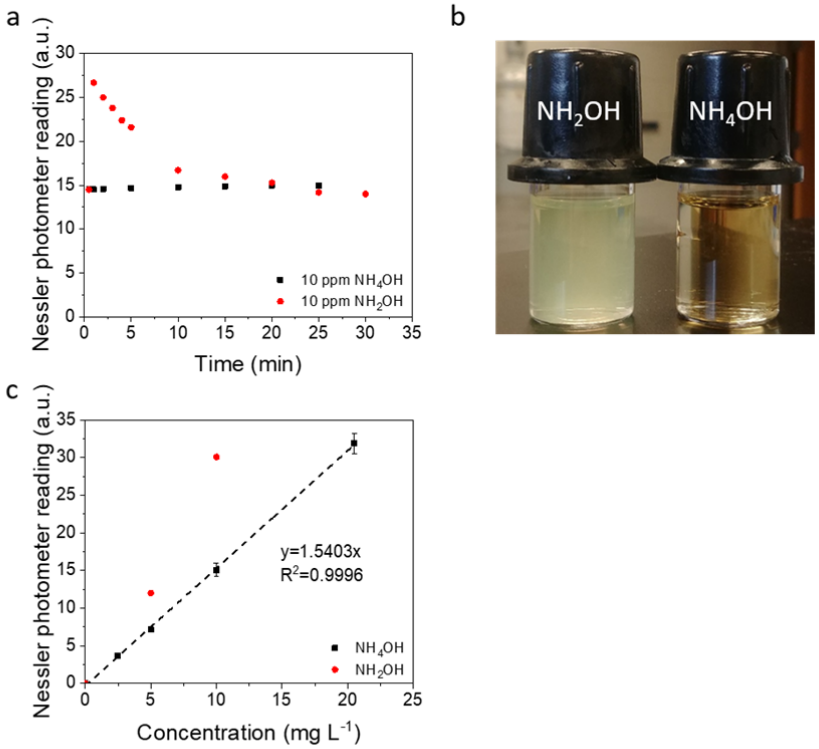

Figure R23. Ammonia quantification using Nessler's method. (a) Nessler photometer reading vs. time of 10 mg L^{-1} NH_4OH and 10 mg L^{-1} NH_2OH. Reaction of NH_4OH is complete almost immediately. (b) Photograph of 10 mg L^{-1} NH_2OH (left) and 10 mg L^{-1} NH_4OH (right) at 5

minutes. (c) Calibration curve for NH_4OH. Absorbance was measured at 1 minute. NH_2OH have large interference, invalidating the quantification of NH_4OH in the presence of NH_2OH. All solutions were prepared in 0.25M $KHCO_3$ to match the condition of the liquid products in the electrolyte.

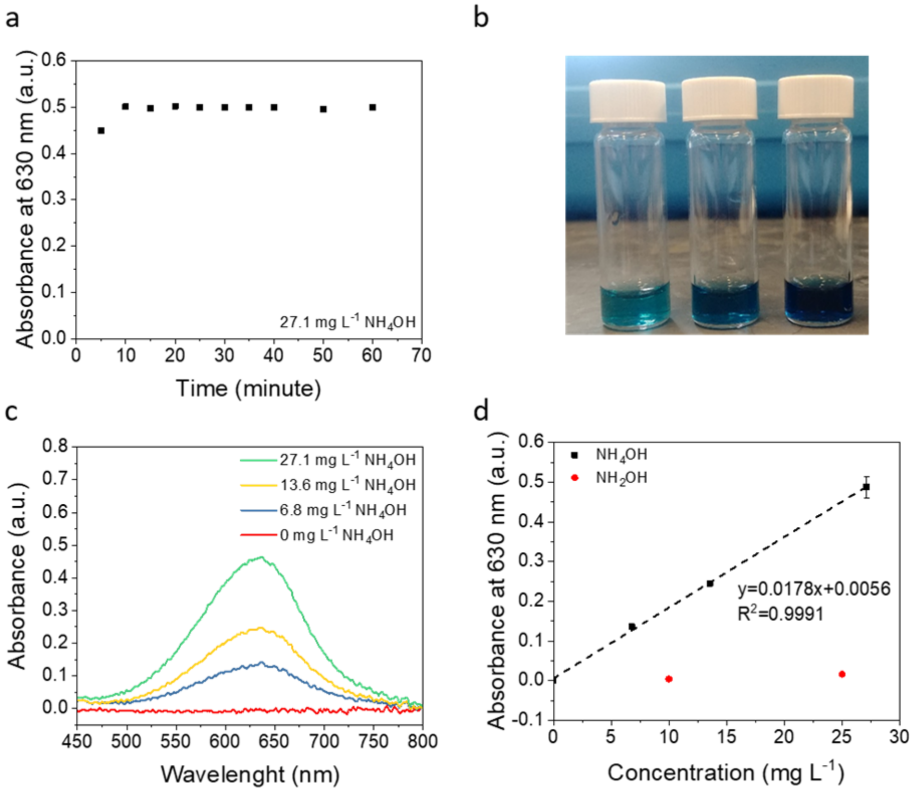

Figure R24 (New Figure S13). Ammonia quantification using indophenol blue method. (a) Absorbance vs. time of 27.1 mg L^{-1} NH_4OH. Reaction is complete after 10 minutes. (b) Photograph of 6.8, 13.6, and 27.1 mg L^{-1} NH_4OH (from left to right) at 20 minutes. (c) Absorption spectra for different concentrations of NH_4OH measured at 20 minutes. (d) Calibration curve for NH_4OH. Absorbance was measured at 20 minutes. NH_2OH has negligible interference. All solutions were prepared in 0.25M $KHCO_3$ to match the condition of the liquid products in the electrolyte.

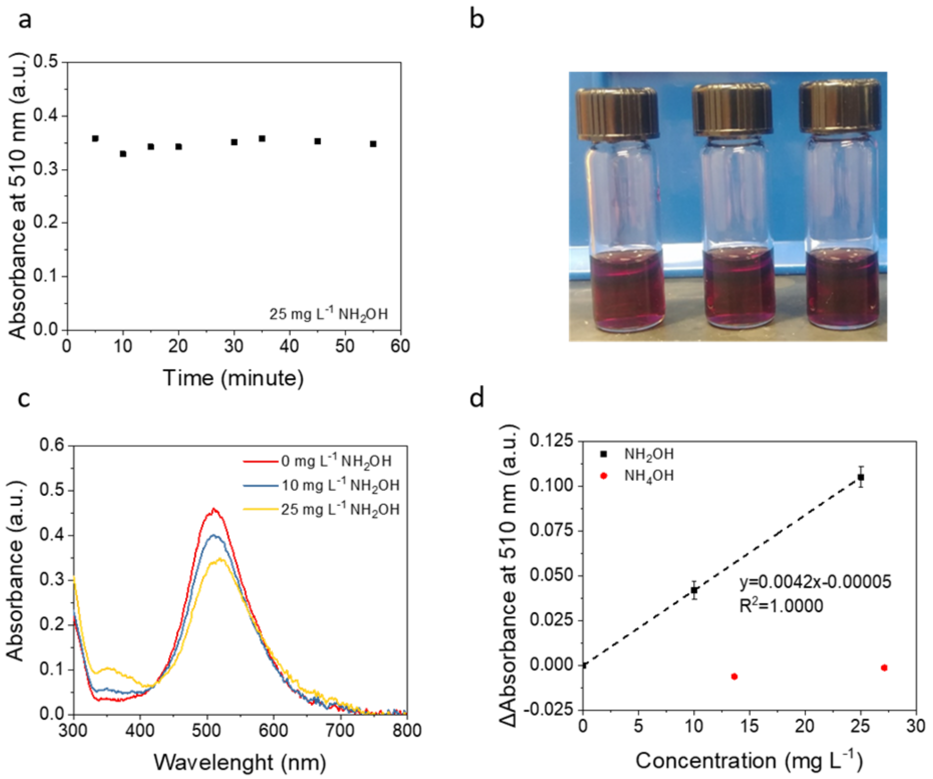

Figure R25 (New Figure S14). Hydroxylamine quantification. (a) Absorbance vs. time of 25 mg L^{-1} NH_2OH. Reaction is complete after 10 minutes. (b) Photograph of 0, 10, and 25 mg L^{-1} NH_2OH (from left to right) at 20 minutes. (c) Absorption spectra for different concentrations of NH_2OH measured at 20 minutes. (d) Calibration curve for NH_2OH. Absorbance was measured at 20 minutes. Absorbance was subtracted from that of 0 mg L^{-1} NH_2OH. NH_4OH has negligible interference. All solutions were prepared in 0.25M $KHCO_3$ to match the condition of the liquid products in the electrolyte.

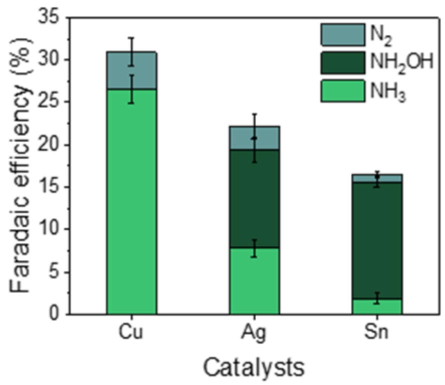

Figure R26 (New Figure 4a). Faradaic efficiency of NO electroreduction products produced during electrolysis with 83.3% CO_2+0.83% NO in Ar on Cu, Ag, and Sn catalysts at a constant current density of 100 mA cm^{-2} in 1 M $KHCO_3$ for 3 h. Error bars represent the standard deviation of three independent measurements.

In the revised manuscript, we have included the details of the methods to determine NH_4OH and NH_2OH, included references, updated the NORR products, and revised the discussion on

the NORR products as shown below:

"NH_3 was quantified using indophenol blue method[41] with UV-vis spectroscopy (Nanodrop 2000, Thermo Scientific). 100 µL of the sample was mixed with 500 µL of alkaline hypochlorite solution (A1727, Sigma-Aldrich) and 500 µL of phenol nitroprusside solution (P6994, Sigma-Aldrich). The solution was incubated in the dark at room temperature for 20 minutes. 2 µL of the solution was pipetted onto the pedestal, and the absorbance was measured by UV-vis spectroscopy from 190 nm to 840 nm. The absorbance of the sample was measured at 630 nm, and the background was subtracted with the absorbance measured at 830 nm. The calibration curves were obtained using different concentrations of ammounium hydroxide (NH_4OH; 28.0-30.0%, Sigma Aldrich) in 0.25 M $KHCO_3$ (Fig. S13)." (Page 12, Lines 370-378)

"NH_2OH was quantified using a procedure modified from a previous report by Afkhami et al.[42] with UV-vis spectroscopy (Nanodrop 2000, Thermo Scientific). Neutral red solution was prepared by dissolving 200 mg of neutral red (Sigma-Aldrich) in 100 mL DI. Iodate solution was prepared by dissolving 1.000 g of potassium iodate (KIO_3, 99.995%, Sigma-Aldrich) in 100 mL DI. 500 µL of sample was mixed with 250 µL of 3.0 M sulfuric acid (Fisher Scientific) and 250 µL of iodate solution. After 5 minutes at room temperature, 500 µL of neutral red solution was added to the solution. The solution was incubated at room temperature for 20 minutes. 2 µL of the solution was pipetted onto the pedestal, and the absorbance was measured by UV-vis spectroscopy from 190 nm to 840 nm. The absorbance of the sample was measured at 510 nm, and the background was subtracted with the absorbance measured at 800 nm. The change in absorbance was determined by subtracting the absorbance of the sample solution from the absorbance of the solution with 0 mg L^{-1} NH_2OH. The calibration curves were obtained using different concentrations of hydroxylamine (50 wt % in H_2O, Sigma Aldrich) in 0.25 M $KHCO_3$ (Fig. S14)." (Page 12, Lines 379-392)

"The primary products of NO_x electroreduction include N_2O, N_2, NH_2OH, and NH_3." (Page 1, Lines 19-20)

"… in which the major products are ammonia (NH_3), hydroxylamine (NH_2OH), N_2, and N_2O." (Page 2, Lines 76-77)

"As shown in Fig. 4a, NORR product selectivity varied among different catalysts. Cu primarily produced NH_3 and N_2, with no NH_2OH, Ag produced a mixture of NORR products, and Sn primarily produced NH_2OH. These observations are consistent with previous reports, in which Cu has been demonstrated as an effective catalyst for NORR to NH_3,[27] and Sn has been used as a dopant in Pt to shift the selectivity from NH_3 to NH_2OH in nitrate reduction.[33]" (Page 6 and 7, Lines 193-198)

"Collectively, NH_3, NH_2OH, N_2, and N_2O have been determined as the NORR products." (Page 8, Lines 231)

"The major NORR products are NH_3, NH_2OH, N_2, and N_2O, in which the selectivity varies among different catalysts …" (Page 10, Lines 305-306)

9. Small corrections and typos:

a. I would recommend including Engelbrecht et al. 2017

(https://doi.org/10.1016/j.electacta.2016.12.059) in the literature review – the specific goal of this paper was to oxidize the Cu catalyst by co-feeding O¬2 during CO2 reduction (second effect depicted in your Fig. 1A).

Reply:

We have added the reference to the revised manuscript as reference 21.

b. Two tables are listed as "Table S1" in the supporting information

Reply:

Table numbers have been corrected.

c. Line 188: it's probably best to remind the reader in this spot what the loss of CO2R FE was during these tests so they don't have to go back and look to check that this is a true statement.

Reply:

In the revised manuscript, the following sentence has been added:

> "While the losses of CO_2RR FE were 11.4%, 10.2%, and 1.4% on Cu, Ag, and Sn catalysts, respectively, the amounts of N_2 detected were 8.2%, 7.3%, and 0.5% of the total FE, respectively, accounting for the majority of the loss in the CO_2RR FE." (Page 8, Lines 233-235)

d. Table S11 caption: could the authors clarify what they mean by "N content has been calculated relative to metal content"? I would assume this means that the percentage column is equal to 100% times the ratio N/(N plus the nominal metal), but want to clarify.

Reply:

The caption for **Tables S15-17** has been changed to:

> "N content has been calculated using the following equation: N content = $\frac{\Sigma N\ (wt\ \%)}{\Sigma N\ (wt\ \%)+metal\ (wt\ \%)} \times 100$, where ΣN (wt %) = graphitic N (wt %) + pyrollic N (wt %) + pyridinic N (wt%) and metal = Cu or Ag or Sn."

e. Methods line 266: I'm guessing "Sn (0.1 m, Alfa Aesar)" really means 0.1 um rather than 0.1 m. Those are some big particles if not! :)

Reply:

Sorry for the typo. We have corrected the unit to 0.1 µm.

f. Line 305-306: SEM here refers to secondary electron multiplier – I might suggest using terminology which corresponds to the acronym so that it's not confusing.

Reply:

We have removed "SEM" and used "secondary electron detection."

This is an important contribution to the literature - if the revisions above are made, I would advocate for accepting the article.

Reviewer #1 (Remarks to the Author):

I appreciate the comprehensive and clear revision. This paper is ready for publication and I will cite it.

Reviewer #2 (Remarks to the Author):

To the best of the reviewer's judgement, Jiao and co-workers have thoroughly addressed all comments from the first-round referees. The work is scientifically robust.

However, the authors' fully justified response to the initial comment on the NOx removal and additional clarification on the typical NOx levels in CO2 further convinces the present reviewer that this work is too specialised for Nature Communications.

Reviewer #3 (Remarks to the Author):

The authors have addressed in great detail my comments regarding site blocking, transport, gas compositions, consistent conditions, oxidation state of catalyst, and FE closure. The work is now suitable for publication.
